# NET-02: a randomised, non-comparative, phase II trial of nal-IRI/5-FU or docetaxel as second-line therapy in patients with progressive poorly differentiated extra-pulmonary neuroendocrine carcinoma

**DOI:** 10.1016/j.eclinm.2023.102015

**Published:** 2023-06-02

**Authors:** Mairéad G. McNamara, Jayne Swain, Zoe Craig, Rohini Sharma, Olusola Faluyi, Jonathan Wadsley, Carys Morgan, Lucy R. Wall, Ian Chau, Nick Reed, Debashis Sarker, Jane Margetts, Daniel Krell, Judith Cave, Sharmila Sothi, Alan Anthoney, Christopher Bell, Alkesh Patel, Jamie B. Oughton, David A. Cairns, Wasat Mansoor, Angela Lamarca, Richard A. Hubner, Juan W. Valle

**Affiliations:** a Division of Cancer Sciences, University of Manchester, Manchester, UK; b The Christie NHS Foundation Trust, Manchester, UK; c Leeds Cancer Research UK Clinical Trials Unit, University of Leeds, Leeds, UK; d Imperial College, London, UK; e Clatterbridge Cancer Centre, Liverpool, UK; f Weston Park Hospital, Sheffield, UK; g Velindre Cancer Centre, Cardiff, UK; h Western General Hospital, Edinburgh, UK; i The Royal Marsden NHS Foundation Trust, London, UK; j Beatson Oncology Centre, Glasgow, UK; k King's College Hospital, London, UK; l Newcastle upon Tyne Hospitals NHS Foundation Trust, Newcastle upon Tyne, UK; m Royal Free London, London, UK; n Southampton University Hospitals NHS Trust, Southampton, UK; o University Hospital Coventry, Coventry, UK; p Leeds Teaching Hospitals NHS Trust, Leeds, UK

**Keywords:** Neuroendocrine carcinoma, Second-line treatment, Liposomal irinotecan, Docetaxel, Quality of life

## Abstract

**Background:**

The prognosis for patients with poorly-differentiated extra-pulmonary neuroendocrine carcinoma (PD-EP-NEC) is poor. A recognised first-line (1L) treatment for advanced disease is etoposide/platinum-based chemotherapy with no standard second-line (2L) treatment.

**Methods:**

Patients with histologically-confirmed PD-EP-NEC (Ki-67 > 20%; Grade 3) received IV liposomal irinotecan (nal-IRI) (70 mg/m^2^ free base)/5-FU (2400 mg/m^2^)/folinic acid, Q14 days (ARM A), or IV docetaxel (75 mg/m^2^), Q21 days (ARM B), as 2L therapy. Primary endpoint was 6-month progression-free survival (PFS) rate (80% power to demonstrate one-sided 95% lower confidence interval excluded 15% (target level of efficacy: 30%)). Secondary endpoints: objective response rate (ORR), median PFS, overall survival (OS), toxicity and patient-reported quality-of-life (QoL) (ClinicalTrials.gov: NCT03837977).

**Findings:**

Of 58 patients (29 each arm); 57% male, 90% ECOG PS 0/1, 10% PS 2, 89.7% Ki-67 ≥ 55%, primary site: 70.7%-gastrointestinal, 18.9%-other, 10.3%-unknown, 91.4%/6.9%/1.7% were resistant/sensitive/intolerant to 1L platinum-based treatment, respectively. The primary end-point of 6-month PFS rate was met by ARM A: 29.6% (lower 95% Confidence-Limit (CL) 15.7), but not by ARM B: 13.8% (lower 95%CL:4.9). ORR, median PFS and OS were 11.1% (95%CI:2.4–29.2) and 10.3% (95%CI:2.2–27.4%); 3 months (95%CI:2–6) and 2 months (95%CI:2-2); and 6 months (95%CI:3–10) and 6 months (95%CI:3–9) in ARMS A and B, respectively. Adverse events ≥ grade 3 occurred in 51.7% and 55.2% (1 and 6 discontinuations due to toxicity in ARMS A and B), respectively. QoL was maintained in ARM A, but not ARM B.

**Interpretation:**

nal-IRI/5-FU/folinic acid, but not docetaxel, met the primary endpoint, with manageable toxicity and maintained QoL, with no difference in OS. ORR and median PFS were similar in both arms. This study provides prospective efficacy, toxicity and QoL data in the 2L setting in a disease group of unmet need, and represents some of the strongest evidence available to recommend systemic treatment to these patients.

**Funding:**

10.13039/501100011725Servier.

**Research in context**

**Evidence before this study**

The prognosis for patients with advanced poorly-differentiated extra-pulmonary neuroendocrine carcinoma (PD-EP-NEC) is poor, and usually less than 1 year. A recognised first-line treatment option in this setting is etoposide/platinum-based chemotherapy, with no standard second-line treatment. A systematic review and meta-analysis of studies was previously conducted, and included patients with PD-EP-NEC receiving treatment in the second-line advanced setting. Electronic databases were reviewed (MEDLINE [host: OVID] and EMBASE [host: OVID]) from 1996 to October 31, 2018 and this was supplemented by a manual search of the American Society of Clinical Oncology abstracts 2016 to 2018 and European Society for Medical Oncology abstracts 2016 to 2018. Search terms included "second line", "neuroendocrine carcinoma∗", "neuro-endocrine carcinoma∗", "platinum". This review and meta-analysis included 19 eligible studies (N = 582) (predominantly retrospective and single-arm prospective studies (there were no randomised prospective studies)) and reported a pooled median objective response rate (ORR), median progression-free survival (PFS) and overall survival (OS) of 18%, 2.5 and 7.6 months respectively.

**Added value of this study**

Liposomal irinotecan/5-fluorouracil/folinic acid, but not docetaxel, met the primary end-point of 6-month PFS rate, with the one-sided 95% confidence limit excluding 15% and reaching the threshold for efficacy, with manageable toxicity and maintained Quality of life. ORR, median PFS and OS were similar in both arms. The NET-02 trial provides randomised prospective efficacy, toxicity and importantly, quality-of-life data, for liposomal irinotecan/5-fluorouracil/folinic acid, and docetaxel in the second-line advanced setting in a disease group of unmet need (PD-EP-NEC), and represents some of the strongest evidence available to recommend systemic treatment to these patients.

**Implications of all the available evidence**

Patients with PD-EP-NEC are a challenging population to treat, and there is no standard of care in the second-line advanced setting. There has been a paucity of randomised prospective studies and research to date in this disease group, and setting, and this study provides efficacy data that may be able to guide future study design, including quality-of-life data, and reinforces the urgent need for further research in this disease group to develop more effective therapeutic strategies in a population with a poor prognosis. These results also highlight the challenges in improving survival for these patients and that conduct of randomised trials in this disease group is possible and safe.

## Introduction

The incidence of neuroendocrine neoplasms (NENs) has increased 6.4-fold over a 39-year period, up to 2012, according to data from the Surveillance, Epidemiology, and End Results (SEER) programme,^1^ with increases also being observed in the United Kingdom.^2^ Neuroendocrine neoplasms constitute approximately 2% of all gastrointestinal (GI) malignancies by incidence,^3^ and encompass well-differentiated neuroendocrine tumours (WD-NETs) and poorly-differentiated neuroendocrine carcinomas (PD-NECs), which are characterised by a more aggressive histology with a Ki-67, by definition, greater than 20%.^4^, ^5^, ^6^ Poorly-differentiated NECs represent only 10–20% of all NENs, and the majority of PD-NECs arise from the lung, with approximately 9% being extra-pulmonary in origin; of these, 37.4% have a GI primary site, 34.4% have other as their primary site (example: breast, head and neck, or genitourinary) and 28.2% have unknown primaries.^7^

The majority of patients with poorly-differentiated extra-pulmonary NEC (PD-EP-NEC) present with advanced disease, and have a poor prognosis of up to approximately 12 months.^6^^,^^8^ The management of these patients, to date, has been extrapolated from lung NEC, with platinum-based regimens recommended in the first-line advanced setting, and treatment has remained unchanged for over 30 years.^9^ There is no standard treatment for these patients beyond first-line, with a variety of regimens used, with limited efficacy, and this is an area of unmet need. A systematic review and meta-analysis of studies including patients with PD-EP-NEC receiving treatment in the second-line advanced setting, included 19 eligible studies (N = 582) and reported a pooled median response rate, progression-free survival (PFS) and overall survival (OS) of 18%, 2.5 and 7.6 months respectively.^10^

Irinotecan has been used in the first-line advanced setting for patients with PD-NEC; in a Japan Clinical Oncology group phase III randomised study, irinotecan plus cisplatin in advanced NEC of the digestive system was not inferior to etoposide/cisplatin in terms of OS,^8^ with comparable results reported in a randomised phase II study, using the same regimens and in a similar setting.^11^ At the time of protocol development, there was no standard second-line treatment option in patients with PD-EP-NEC. The combination regimen 5-fluorouracil (5-FU)/irinotecan is a second-line therapy option in patients with PD-EP-NEC, used historically without trial evidence,^6^^,^^12^ until the results of the more recent French PRODIGE 41-BEVANEC randomised phase II study evaluating bevacizumab in combination with 5-FU/irinotecan (FOLFIRI) (experimental arm) versus FOLFIRI alone after the failure of platinum-etoposide in patients with advanced PD-EP-NEC trial.^13^^,^^14^

In one of the largest retrospective studies of patients with advanced gastrointestinal NEC (NORDIC NEC study), 33% of patients (N = 100) received second-line treatment, with 20 of those receiving docetaxel.^15^ In the previously mentioned systematic review and meta-analysis of studies including patients with PD-EP-NEC receiving treatment in the second-line advanced setting, 15 different regimens were used, with no optimal choice.^10^ As treatment for PD-EP-NEC in the first-line advanced setting is similar to that of small cell lung cancer, and as a second-line therapy option for patients with high grade NEC of the lung is docetaxel, as per the National Comprehensive Cancer Network Clinical Practice Guidelines in Oncology (NCCN Guidelines®) (Small cell lung cancer),^16^ this regimen was chosen as an alternative arm in NET-02 when the protocol was being developed, acknowledging that there are no prospective studies of docetaxel, or many other chemotherapeutic options, in patients with PD-EP-NEC.

Liposomal irinotecan (nal-IRI) (ONIVYDE®, Servier) comprises irinotecan free base, encapsulated in liposome nanoparticles, and is reported to keep irinotecan in the circulation longer than non-encapsulated irinotecan, thus has the potential to increase and prolong the intratumoural levels of both irinotecan and SN-38 (the active metabolite), compared with free irinotecan,^17^ and may have clinical benefit in patients with PD-EP-NEC, acknowledging that its superior efficacy over irinotecan has never been proven in the clinical setting.

Therefore, the aim of the NET-02 trial was to assess the efficacy of nal-IRI/5-FU/folinic acid or docetaxel, separately, as second-line therapy options in patients with progressive PD-EP-NEC, with selection criteria applied to establish which treatment to take forward to a phase III trial.

## Methods

### Study patients

The NET-02 trial was a United Kingdom (UK), multi-centre, randomised (1:1), parallel group, open-label, phase II, single-stage selection trial of nal-IRI/5-FU/folinic acid or docetaxel as second-line therapy in patients with progressive PD-EP-NEC (Carcinoma of unknown primary was allowed if lung primary had been excluded following multi-disciplinary meeting review of radiological investigations and immunohistochemical profile). It was conducted under the auspices of the National Institute for Health Research Clinical Research Network in the UK. Eligible patients had histologically-confirmed advanced PD-EP-NEC grade 3 (G3) as per the World Health Organisation 2019 (version updated from 2010 to 2017 in protocol amendments) with a Ki-67 > 20%^5^ (Mixed Neuroendocrine-non Neuroendocrine Neoplasm was not eligible). Regional pathology review was performed (no central review), which was predominantly in European Neuroendocrine Tumour Society (ENETS) Centres of Excellence (11 of 14 sites, accounting for 88% patients included). Patients must have received prior treatment with first-line platinum-based chemotherapy. Patients required radiological evidence of disease progression as per Response Evaluation Criteria In Solid Tumours (RECIST) 1.1,^18^ or discontinuation of first-line platinum-based chemotherapy due to intolerance. Patients had to be ≥ 18 years old and have a life expectancy >3 months with an Eastern Cooperative Oncology Group performance status (ECOG PS) ≤2. Patients were excluded if they had received previous treatment (for NEC) with any of the components of combination chemotherapy regimens detailed in this study (nal-IRI, 5-FU, irinotecan, other topoisomerase inhibitors or taxane-based therapy), had incomplete recovery from previous therapy, including ongoing peripheral neuropathy of Common Terminology Criteria for Adverse Events (CTCAE) v5.0 >G2 from previous platinum-based therapy, and patients must not have had a history of other malignant diseases (within the previous 3 years, and there must be no evidence of recurrence). Complete eligibility criteria are provided in the protocol (online only) and trial in progress manuscript.^19^ This study had ethical approval from the Greater Manchester Central Research Ethics Committee (Reference No. 18/NW/0031) and clinical trial authorisation from the Medicine and Healthcare Products Regulatory Agency, and was conducted in accordance with the Declaration of Helsinki and Good Clinical Practice guidelines. Written informed consent was obtained from all patients. The trial was registered on ClinicalTrials.gov (NCT03837977).

### Randomisation and masking

Patients were enrolled and treated by the investigators at participating study centres on an outpatient basis. Eligible patients were randomised on a 1:1 basis to receive either liposomal irinotecan in combination with 5-FU/folinic acid or docetaxel. Randomisation was performed by the Clinical Trials Research Unit (CTRU), Leeds, using either the CTRU automated 24-h telephone randomisation system or through the CTRU web-based system. Stratification factors used for randomisation are described in the statistical analyses section. There was no masking of study treatment.

### Study procedures

Patients were randomised (1:1) to receive nal-IRI 70 mg/m^2^ free base (updated in amendment from original dose 80 mg/m^2^ (expressed in salt base, which is equivalent to the 70 mg/m^2^ free base), due to product change from 5 mg/ml irinotecan hydrochloride trihydrate to 4.3 mg/ml irinotecan anhydrous free-base; the amount of active ingredient was not changed), 5-FU (2400 mg/m^2^) and folinic acid intravenously (IV) every 14 days or docetaxel 75 mg/m^2^ IV every 21 days with granulocyte colony stimulating factor (G-CSF; mandated by Data Safety Monitoring Board (DSMB) after 11 patients had been recruited to the docetaxel arm due to the proportion of ≥grade 3 neutropenia occurrences). Treatment continued until disease progression, intolerable toxicity, or patient/physician decision to withdraw. Computed tomography imaging was performed every 8 weeks and reported as per RECIST 1.1. Peripheral blood neuron specific enolase (NSE) (μg/L) was measured at baseline, every 6 weeks and at disease progression. Baseline variables for the GI-NEC prognostic score were recorded; presence of liver metastases, alkaline phosphatase, lactate dehydrogenase, ECOG PS, and Ki-67 (The GI-NEC score splits patients into a good (score 0–2) or bad prognosis (score 3–6) group).^20^ Adverse events were recorded throughout (CTCAE v5.0).

Quality of life was assessed using the European Organisation for Research and Treatment of Cancer (EORTC) Core Quality-of-Life questionnaire (QLQ-C30) and the disease-specific QLQ-GINET21, which was previously devised to supplement the QLQ-C30, including quality-of-life issues important to patients with NETs (there is no specific questionnaire available for patients with PD-EP-NEC) (assessed at baseline and every 6 weeks thereafter).^21^ All of the QLQ-C30 scales and single-item measures range from 0 to 100. A high score for a functional scale represents a high/healthy level of functioning and a high score for global health status represents a high quality-of-life. A high score for a symptom scale/item, represents a high level of symptomatology/problems. Bloods for translational analysis were drawn at baseline, 6 weeks and on progression (results to be reported separately).

### Statistical analysis

The primary end-point was 6-month PFS rate, defined as a binary outcome (progression-free or not) within the timeframe of treatment start date until 6 months after randomisation (6-month PFS rate was chosen as the primary end-point as a potential surrogate for OS and for efficiency in relation to study duration, looking for a potential signal of efficacy). The primary analysis population was defined as those who received at least one dose of the protocol treatment and was not a major protocol violator. Major protocol violators were those not diagnosed with PD-EP-NEC. An adaptation of a one-stage trial design proposed by Simon et al.^22^ was used where the A'Hern design^23^ was first implemented to assess the efficacy of each treatment separately, to ensure a prespecified minimum level of activity prior to selection. Allowing for a 5% drop out rate, with the aim to recruit 51 patients to each arm, this would provide 80% power to demonstrate that the one-sided 95% confidence interval (CI) for the 6-month PFS rate excluded 15%, if the true rate was at least 30%, where 30% was the target level of efficacy and a rate of 15% or less would give grounds for rejection.^19^ The values of 15% and 30% were chosen based on review of the existing literature where 6-month PFS rate was reported and ranged from 15 to 25%.^10^ The intention was to show that the regimens were sufficiently active, but not to assess superiority of one regimen over the other. It was planned that a treatment arm would be considered for further evaluation if at least 12 of 48 evaluable patients were progression free at 6 months. If both treatments exceeded the predefined criteria of having one-sided 95% confidence limits >15%, the design proposes that the treatment with the higher PFS rate at 6 months would be selected.

Secondary end-points included objective response rate (ORR) (defined as the proportion of patients achieving complete or partial response within 6 months after randomisation), median PFS (defined as the time from randomisation to progression or death from any cause), OS (defined as the time from randomisation to death from any cause), toxicity, and quality-of-life. Individuals were to be censored if they were lost to follow-up or still alive and progression-free at the time of analysis.

Stratification factors used for randomisation included: hospital site, Ki-67 (<55% vs ≥ 55%), ECOG PS (0/1 vs 2), Presence of liver metastases (Yes/No), response to platinum-based chemotherapy (resistant: progression during platinum treatment or progression ≤6 months from platinum completion, sensitive: progression >6 months from platinum completion, or intolerant, acknowledging that there is no standard definition and a lack of quality evidence to support this description in PD-EP-NEC, and thus this wording has been adapted from accepted ovarian cancer practice).^24^ Summary statistics and Kaplan–Meier survival curves for PFS and OS are presented. A logistic regression model was used to assess association between baseline NSE and PFS status at 6 months post randomisation (Baseline NSE was entered into the model as a continuous variable. A smoothed scatter plot contrasting NSE with the progression outcome on the logit scale showed a reasonably linearly trend and hence was considered a reasonable modelling decision). A log-rank test was used to assess association of the GI-NEC score with OS. An exploratory multivariable Cox regression analysis was performed to assess the effect of treatment on PFS and OS, adjusted for the stratification factors, excluding centre. Quality-of-life was summarised using mean scores for each subscale. Mean change from baseline was estimated and results were presented where there were ≥4 responses per timepoint. Scores were considered stable if it did not change by ≥ 10 points from baseline.^25^

Data collection, monitoring and statistical analysis was conducted by members of the Leeds Cancer Research UK Clinical Trials unit. All analyses were performed based on study database download on September 1, 2022, using SAS (v9.4). No formal interim analysis was planned, but the DSMB received full trial reports annually and safety reports, at least 6-monthly, to monitor patient safety and trial progress, with the option to prematurely terminate the trial, if necessary.

## Role of funding

The funder of this study had no role in the study design, data collection, data analysis, data interpretation, or writing of the manuscript.

## Results

The first and last patients were recruited in November 2018 and November 2021 respectively. The DSMB met in December 2021 due to slow recruitment driven by the Coronavirus-19 pandemic (somewhat related to reduced staffing in trial units secondary to re-deployment, which thus resulted in stalling of clinical trial accrual), and recommended performing an analysis to formally predict the probability of a positive trial result if the trial was to continue to the maximum planned sample size for each treatment group, using Bayesian predictive probabilities.^26^ An unfavourable 6-month PFS rate was set at 15% (null hypothesis), a favourable rate was set at 30% (alternative hypothesis), with treatment efficacy defined, using predictive probability, as 95%, and futility set at 10%. For the docetaxel arm, the estimated predictive probability of trial success at 48 patients, given the results observed with the first 26 evaluable patients was 7.9% (4/26 progression-free at 6 months) and for the nal-IRI arm for the first 27 evaluable patients was 96.5% (9/27 progression-free at 6 months). Therefore, it was concluded that the docetaxel arm could be considered futile and that the nal-IRI arm should be considered likely to provide convincing phase II evidence. The DSMB subsequently recommended closure of the trial and release of the results. This was accepted by the independent Trial Steering Committee. The challenges with recruitment remained due to the Coronavirus-19 pandemic and in addition to the case for futility of the docetaxel arm and potential success of the nal-IRI arm, the nal-IRI arm also demonstrated a positive result with respect to the original decision rule, suggesting reasonable evidence for the success of that arm, and its potential confirmation in a future phase III trial.

### Patients and tumour characteristics

**Fig. 1:**
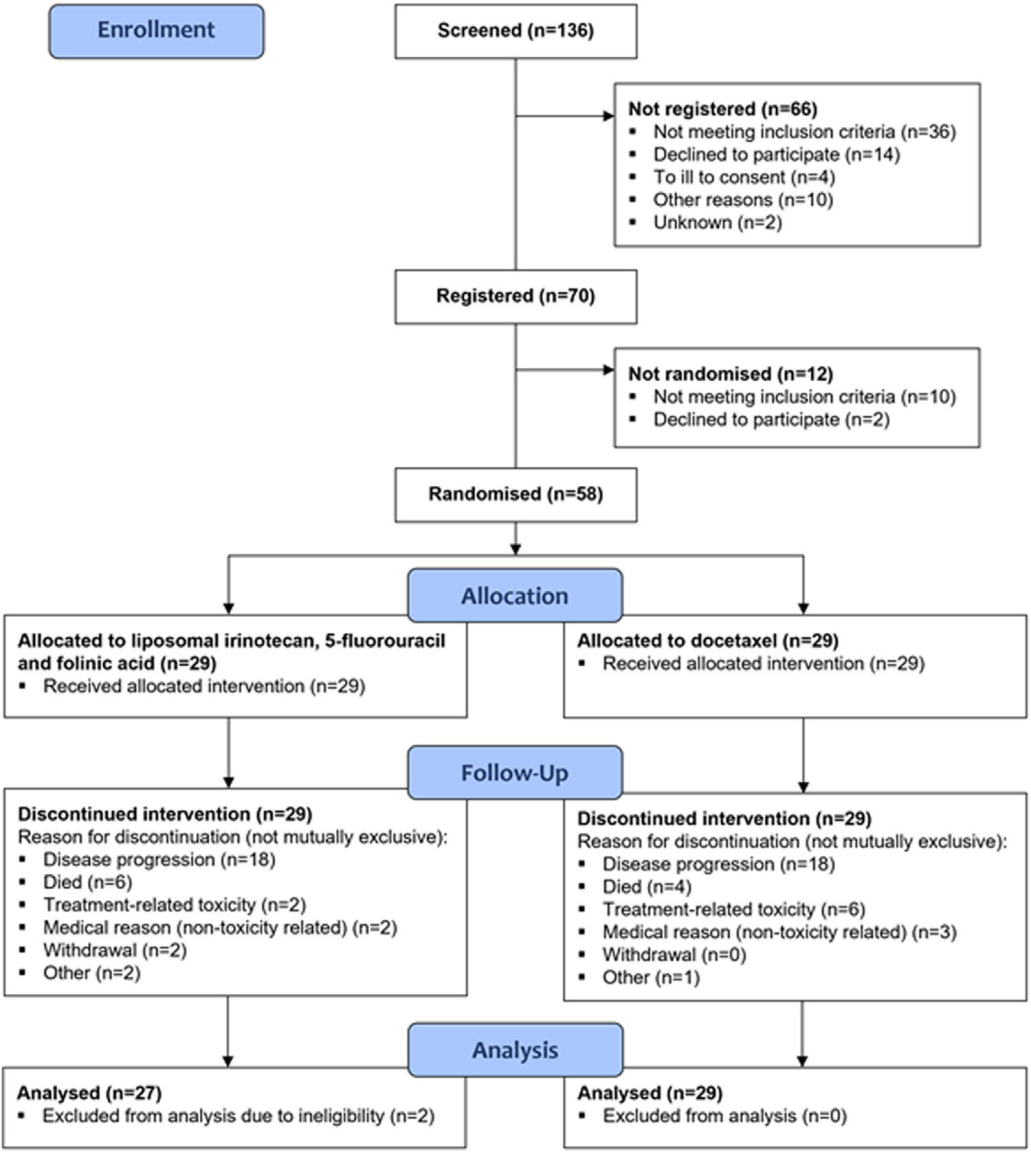
NET-02: Consort diagram.

Fifty-eight patients were recruited (29 in each arm) in 14 UK sites (Fig. 1). Two patients were excluded from the efficacy analysis in the nal-IRI/5-FU/folinic acid group following final data download: one patient had a well-differentiated G3 neuroendocrine tumour and another had a goblet cell adenocarcinoma. All patients were included in the safety analysis. Baseline patient and tumour characteristics for all patients are presented in Table 1. The median age was 63.5 years. Fifty-seven percent were male and the majority had an ECOG PS of 0/1 (90%). There were more patients with a GI primary (70.7%) and morphology was relatively evenly distributed. Eighty-nine point seven percent had a Ki-67 ≥ 55%, 60.3% had liver metastases. Only 8.6% had previous surgery, with 2 patients having received adjuvant platinum-based treatment. The majority (91.4%) were resistant to previous platinum-based treatment in the first-line advanced setting. The median time from end of first-line treatment in the advanced setting and randomisation was 3.3 months (range 1–15) in the liposomal irinotecan arm and 2.8 months (range 1–14) in the docetaxel arm. Previous platinum-based treatment in the entire cohort was Carboplatin (86%), cisplatin (10.5%) and oxaliplatin (3.5%); all combined with etoposide except for two patients. In the nal-IRI arm, 82% received previous carboplatin, 14% cisplatin and 4% oxaliplatin. In the docetaxel arm, 90% received previous carboplatin, 7% cisplatin and 3% oxaliplatin. Eight patients in both arms (27.6% each) received subsequent chemotherapy following NET-02.

**Table 1:** NET-02: Patient and tumour baseline characteristics.

	Liposomal irinotecan/5-FU/folinic acid (N = 29)	Docetaxel (N = 29)	Total (N = 58)
Median age (range)	61.0 (47–85)	66.0 (22–81)	63.5 (22–85)
Sex: Male, N (%)	18 (62)	15 (52)	33 (57)
Ethnicity, N (%):			
White (Caucasian)	26 (89.7)	27 (93.1)	53 (91.4)
Black (African)	1 (3.4)	1 (3.4)	2 (3.4)
Other black background	0 (0.0)	1 (3.4)	1 (1.7)
Not stated	2 (6.9)	0 (0.0)	2 (3.4)
ECOG PS, N (%):			
0/1	26 (90)	26 (90)	52 (90)
2	3 (10)	3 (10)	6 (10)
Primary tumour site, N (%):			
Gastrointestinal	20 (68.9)	21 (72.4)	41 (70.7)
*Upper gastrointestinal*	*8 (27.6)*	*10 (34.5)*	*18 (31.0)*
*Hepatobiliary*	*5 (17.2)*	*8 (27.6)*	*13 (22.4)*
*Lower gastrointestinal*	*7 (24.1)*	*3 (10.3)*	*10 (17.2)*
aOther	5 (17.2)	6 (20.6)	11 (18.9)
Unknown	4 (13.8)	2 (6.9)	6 (10.3)
Morphology, N (%):			
Small cell	10 (34.5)	10 (34.5)	20 (34.5)
Large cell	9 (31)	13 (44.8)	22 (37.9)
bUnknown	10 (34.5)	6 (20.7)	16 (27.6)
Ki-67, N (%):			
<55%	3 (10.3)	3 (10.3)	6 (10.3)
≥55%	26 (89.7)	26 (89.7)	52 (89.7)
Liver metastases, N (%):			
Yes	19 (65.5)	16 (55.2)	35 (60.3)
No	10 (34.5)	13 (44.8)	23 (39.7)
Previous surgery, N (%):	3 (10.3)	2 (6.9)	5 (8.6)
Adjuvant platinum-based treatment	1 (3.4)	1 (3.4)	2 (6.9)
Response to platinum, N (%):			
Resistant	26 (89.7)	27 (93.1)	53 (91.4)
Sensitive	2 (6.9)	2 (6.9)	4 (6.9)
Intolerant	1 (3.4)	0 (0)	1 (1.7)

Due to rounding, percentages may not add up to 100%.

**ECOG PS**: Eastern Cooperative Oncology Group Performance Status, **Platinum resistant**: progression during platinum treatment or ≤6 months from platinum completion, **Platinum sensitive**: progression >6 months from platinum completion.

a **Other primary site:** Breast, genitourinary or head and neck.

b Neuroendocrine carcinoma not otherwise specified.

### Efficacy

**Fig. 2:**
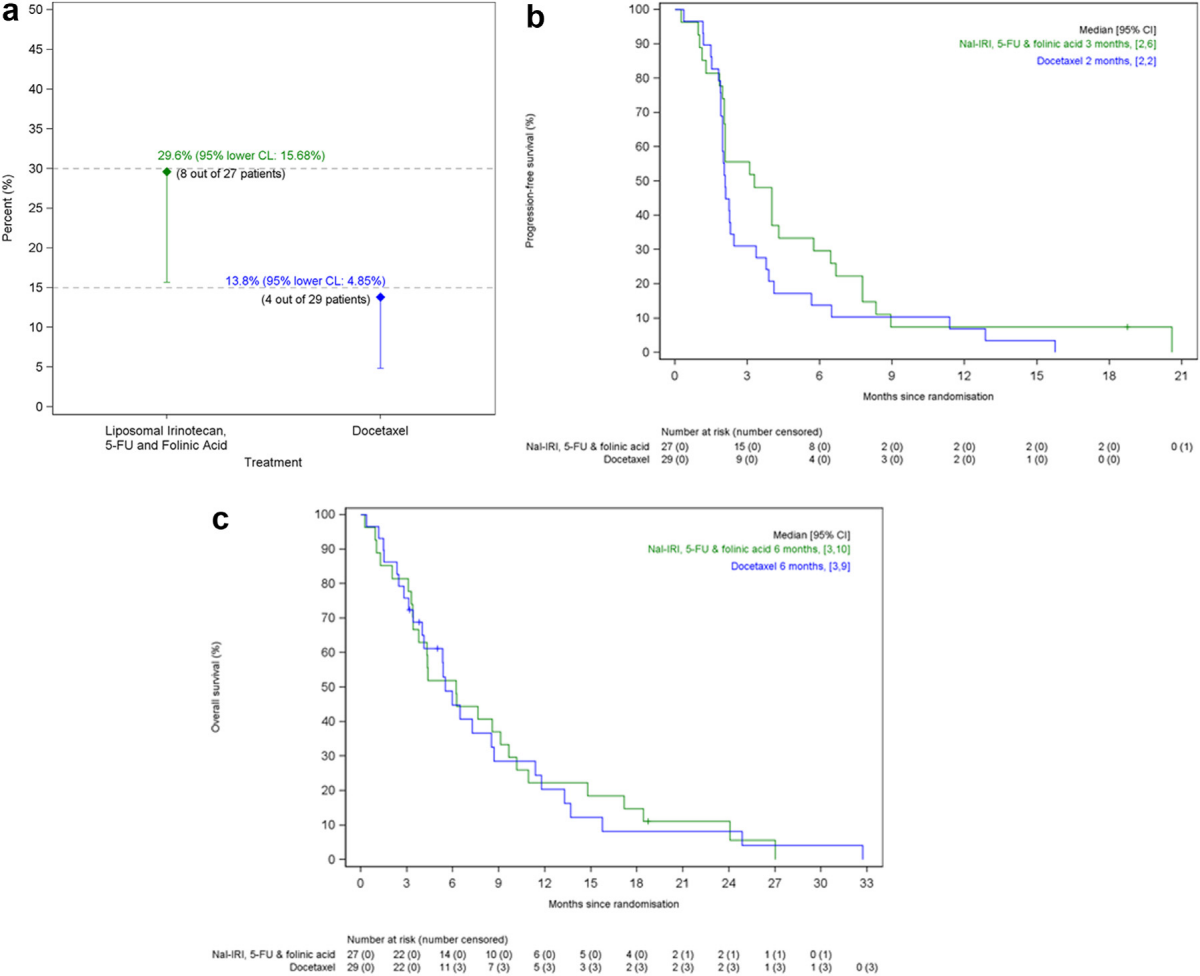
(a) NET-02 primary end-point: 6 month progression-free survival rate. CL: Confidence Limit, 5-FU: 5-fluorouracil. (b) NET-02: Progression-free survival (PFS). Median PFS was 3 months (95% confidence interval (CI) 2–6) in the liposomal irinotecan arm (represented by green line) and 2 months (95% CI 2-2) in the docetaxel arm (represented by blue line). Nal-IRI: liposomal irinotecan, 5-FU: 5-fluorouracil. (c) NET-02: Overall survival (OS) Median OS was 6 months (95% confidence interval (CI) 3–10) in the liposomal irinotecan arm (represented by green line) and 6 months (95% CI 3–9) in the docetaxel arm (represented by blue line). Nal-IRI: liposomal irinotecan, 5-FU: 5-fluorouracil.

No patient was censored due to loss of follow-up whilst on study. No patients were censored for any reason in the period up to the 6-month assessment of the primary endpoint. A single patient was censored in the nal-IRI arm following the 18-month PFS assessment. The primary end-point of 6-month PFS rate was met by nal-IRI/5-FU/folinic acid (29.6% (95% lower confidence limit (CL): 15.7%)), but not docetaxel (13.8% (95% lower CL 4.9%)) (Fig. 2a). In the liposomal irinotecan arm, those that met the 6-month PFS rate had the following primaries: upper GI (N = 3), lower GI and head and neck (N = 2 each), unknown (N = 1); all 8 patients had a Ki-67 ≥ 55%. In the docetaxel arm, the primaries were upper GI (N = 2) and hepato-pancreatico-biliary (HPB) and genitourinary (N = 1 each); 3 of these 4 patients had a Ki-67 ≥ 55%.

The ORR was similar in both arms (within 6 months of randomisation): 11.1% (95% CI 2.4–29.2) (nal-IRI/5-FU/folinic acid) and 10.3% (95% CI 2.2–27.4) (docetaxel) (partial responses: N = 3 both arms; all Ki-67 ≥ 55%). The primaries were unknown (N = 2) and upper GI (N = 1) (2 of 3 patients were progression-free at 6 months (1 of each primary)) in the liposomal irinotecan arm and upper GI (N = 2) and genitourinary (N = 1) (all were progression-free at 6 months) in the docetaxel arm. Following 6 months post randomisation, 1 patient in each arm had a complete response (at 18.7 months in the nal-IRI/5-FU/folinic acid arm and at 9.2 months in the docetaxel arm).

Fifty-three patients (91%) have died. The median follow-up in the 5 patients alive at the end of follow-up was 5.0 months (interquartile range: 3.8–18.7 months). In the liposomal irinotecan vs docetaxel arms, the median PFS and OS were 3 months (95% CI 2–6) vs 2 months (95% CI 2-2) and 6 months (95% CI 3–10) vs 6 months (95% CI 3–9) respectively (Fig. 2b and c).

In all patients, there was no association between baseline NSE and PFS status at 6 months post randomisation (odds ratio 1.0 95% CI 1.0–1.0). The median baseline NSE (range), where measured, was 11.8 μg/L (7.4–198.9) in patients who were progression-free at 6 months post randomisation (N = 11) and 25.6 μg/L (7.5–125.3) in those who were not (N = 35).

The GI-NEC score was available for all 56 patients included in the efficacy analysis. A log rank test found that OS split by GI-NEC score group was significantly different in all patients (Good prognostic group (N = 41): median OS: 6 months (95% CI 5–9) versus poor prognostic group (N = 15): 4 months (95% CI 1–4), P = 0.02) and the nal-IRI/5-FU/folinic acid arm (Good prognostic group (N = 20): median OS: 8 months (95% CI 3–15) versus poor prognostic group (N = 7): 3 months (95% CI 0–4), P = 0.02), but not in the docetaxel group (Good prognostic group (N = 21): median OS 6 months (95% CI 3–9) versus poor prognostic group (N = 8) 4 months (95% CI 0–16), P = 0.39).

In an exploratory multivariable Cox regression analysis of PFS in the primary analysis population, there was no effect of treatment group (nal-IRI/5-FU/folinic acid versus docetaxel) (Hazard Ratio (HR) 0.67, 95% CI 0.4–1.2, P = 0.15), Ki-67 (≥55% versus <55%) (HR 1.06, 95% CI 0.4–3.2, P = 0.92), ECOG PS (2 versus 0/1) (HR 1.05, 95% CI 0.3–3.2, P = 0.94), liver metastases (yes versus no) (HR 1.36, 95% CI 0.8–2.5, P = 0.30), or response to previous platinum-based chemotherapy (sensitive/intolerant versus resistant) (HR 0.45, 95% CI 0.2–1.4, P = 0.16) adjusting for the other variables.

In an exploratory multivariable Cox regression analysis of OS in the primary analysis population, there was no detectable statistically significant effect of a number of variables including, treatment group (nal-IRI/5-FU/folinic acid versus docetaxel) (HR 0.80, 95% CI 0.5–1.4, P = 0.46), Ki-67 (≥55% versus <55%) (HR 1.16, 95% CI 0.3–4.5, P = 0.83), ECOG PS (2 versus 0/1) (HR 2.07, 95% CI 0.6–6.8, P = 0.23), or response to previous platinum-based chemotherapy (sensitive/intolerant versus resistant) (HR 0.42, 95% CI 0.1–1.4, P = 0.15). The presence of liver metastases (yes versus no) resulted in a worse OS (HR 2.31, 95% CI 1.2–4.5, P = 0.01).

### Treatment exposure, summary adverse events and reasons for stopping treatment

The treatment exposure, summary adverse events and reasons for stopping treatment are presented in Table 2. The median treatment cycles received was similar in both arms, but the upper range of cycles received in the liposomal irinotecan arm was greater at 32. Dose reductions for treatment-related toxicity were 31% and 28% in the liposomal irinotecan and docetaxel arms, respectively. The predominant reason for patients stopping treatment was due to progression (62.1%), and there was 1 treatment-related death due to neutropenic sepsis in the liposomal irinotecan arm.

**Table 2:** NET-02: Treatment exposure, summary adverse events and reasons for stopping treatment (not mutually exclusive).

	Liposomal irinotecan/5-FU/folinic acid (N = 29)	Docetaxel (N = 29)
Median treatment cycles received (range)	4 (1–32)	3 (1–7)
Dose reduction (treatment-related toxicity), N (%)	9 (31)	8 (28)
Any AE, N (%)	27 (93.1)	28 (96.6)
Any AR, N (%)	24 (82.8)	27 (93.1)
Any ≥ grade 3 AE, N (%)	15 (51.7)	16 (55.2)
Any ≥ grade 3 AR, N (%)	9 (31)	13 (44.8)
Any serious AE, N (%)	10 (34.5)	7 (24.1)
Any serious AR, N (%)	11 (37.9)	12 (41.4)
**Reason for stopping treatment:**		
Progression	18 (62.1)	18 (62.1)
Treatment-related toxicity	2 (6.9)	6 (20.7)
Medical reason (non-toxicity related)	2 (6.9)	3 (10.3)
Withdrawal (participant/clinician driven)	2 (6.9)	0 (0)
Death	6 (20.7)	4 (13.8)
aOther	2 (6.9)	1 (3.4)
bDeath (treatment-related)	1 (3.4)	0 (0)

Toxicity assessed as per Common Terminology Criteria for Adverse Events (CTCAE) version 5.0, **5-FU**: 5-fluorouracil, **AE**: Adverse event, **AR**: Adverse reaction.

a Other: N = 2: Impact on quality of life and >28 day treatment delay, N = 1: Decreased performance status.

b Neutropenic sepsis.

**Fig. 3:**
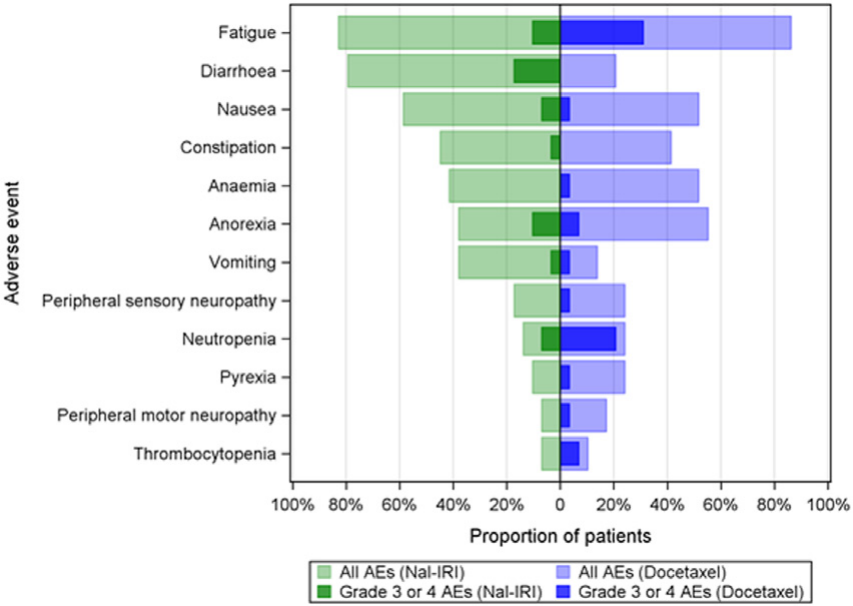
Most common any grade adverse events and ≥grade 3 events (per patient; worst grade anytime). Toxicity assessed as per Common Terminology Criteria for Adverse Events (CTCAE) version 5.0, AE: Adverse event, Nal-IRI: Liposomal irinotecan.

The most common any grade adverse events and ≥ G3 events (per patient; worst grade any time) are presented in Fig. 3. The most common any grade adverse events were fatigue (82.8%), diarrhoea (79.3%), nausea (58.6%), constipation (44.8%) and anaemia (41.4%) in the liposomal irinotecan arm, and fatigue (86.2%), anorexia (55.2%), nausea (51.7%) and anaemia (51.7%) in the docetaxel arm. The most common ≥ G3 events were diarrhoea (17.2%) and fatigue (10.3%) in the liposomal irinotecan arm and fatigue (31%) and neutropenia (20.7%) in the docetaxel arm.

### Quality-of-life

**Supplementary Figure S1:**
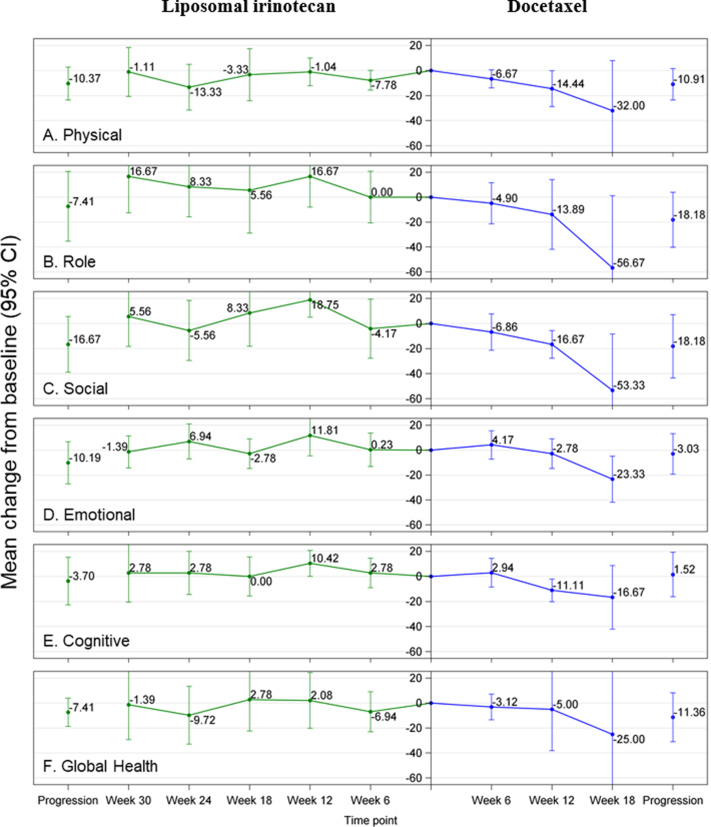


The quality-of-life questionnaires (EORTC QLQ-C30 and GINET21) were completed at baseline by 53 patients, 29 completed ≥1 post-baseline questionnaire and 21 patients completed the questionnaires at progression. In the liposomal irinotecan arm, role functioning showed sustained improvement from baseline and all other EORTC QLQ-C30 functional scales and global health status remained stable pre-progression (Figure S1). In the docetaxel arm, all EORTC QLQ-C30 functional scales and global health status worsened between baseline and week 18 (Figure S1). The patterns of change in the EORTC QLQ-C30 symptom items and GINET21 items from baseline are presented in Table S1. From baseline, there was worsening of C30 symptoms of constipation and diarrhoea in the liposomal irinotecan arm and worsening of fatigue and nausea/vomiting in the docetaxel arm. In the liposomal irinotecan arm, there was improvement in the following C30 items: financial problems, insomnia and pain. In the GINET21 items, there was improvement in body image, disease-related worries, information provided on disease and treatment and social functioning in the liposomal irinotecan arm, and improvement in social functioning in the docetaxel arm. There was worsening of treatment-related symptoms in the docetaxel arm (Table S1).

## Discussion

In NET-02, nal-IRI/5-FU/folinic acid, but not docetaxel, met the primary end-point of 6-month PFS rate, with the one-sided 95% CL excluding 15% and reaching the threshold for efficacy. Objective response rate, median PFS and OS were similar in both arms. Toxicity was as expected, with no new safety signals.^17^^,^^27^ Health-related quality-of-life was maintained with nal-IRI/5-FU/folinic acid, but not docetaxel, in patients with PD-EP-NEC, acknowledging the limitations associated with numbers of patients included in this trial and completing questionnaires.

Recently, another randomised trial in the second-line advanced setting in this disease group has been published; the French PRODIGE 41-BEVANEC randomised phase II trial evaluated bevacizumab in combination with 5-FU/irinotecan (FOLFIRI) (experimental arm) versus FOLFIRI alone after the failure of platinum-etoposide in patients with advanced PD-EP-NEC. It met its primary end-point (6 month OS rate of ≥50% in the experimental arm).^13^^,^^14^ The 6 month OS rate was 52.5% in the FOLFIRI/bevacizumab arm and 59.7% in the FOLFIRI arm. The median PFS and OS were 3.7 months and 6.6 months in the FOLFIRI/bevacizumab arm and 3.5 and 8.9 months in the FOLFIRI arm, respectively. The above study provides further evidence to support the therapeutic value of topoisomerase inhibitors in this disease group and setting, and reinforces the need for further research in this disease group to develop more effective therapeutic strategies. Cross trial comparisons are difficult to make, and it is unlikely that there will be head-to-head comparative trials in this disease group or others. However, the importance of reporting the findings of prospective trials, and particularly randomised, in this disease group cannot be over emphasised, in a subject area which has been poorly researched, to date. The global community can thus interpret and decide on therapy choice based on best available evidence.

There is a phase I/II single-arm first-line trial currently registered (not yet recruiting) on Clinical Trials.gov (NCT05385861) of liposomal irinotecan combined with carboplatin in patients with advanced metastatic gastro-entero-pancreatic PD-NEC, with the primary end-point being maximum tolerated dose and recommended phase II dose in the phase I cohort, and tumour response rate in the phase II cohort. The results of this study will add further to the available literature in this disease group.

In the phase II basket trial of dual anti-cytotoxic T-lymphocyte–associated antigen 4 (CTLA4) and anti-programmed cell death protein 1 (PD-1) blockade (ipilimumab and nivolumab) in rare tumours (DART), 19 patients with high-grade NENs were included (18 with extra-pulmonary primaries). These patients had one median line of prior therapy (range 0–3), and had a median Ki-67 of 80%. The 6-month PFS rate reported for that study was 32%, with a median PFS and OS of 2 months and 8.7 months, respectively.^28^ These results are not dissimilar to that of the liposomal irinotecan arm of NET-02, and the option of the combined regimen of ipilimumab and nivolumab (based on the results of the DART trial) has been included as a potential post first-line therapy option for patients with advanced PD-EP-NEC within the NCCN Guidelines®.^29^ However, another study which investigated nivolumab ± ipilimumab in 185 pre-treated patients with advanced, refractory pulmonary or gastro-entero-pancreatic poorly differentiated NEC was stopped early for futility after 50% accrual, reporting an objective response rate of 14.9%, median PFS of 1.9 months and a median OS of 5.8 months with nivolumab and ipilimumab,^30^ acknowledging that this study included patients with lung NEC, which may have influenced results.

The reason why a difference in the primary end-point of 6-month PFS rate in NET-02 is not translated into OS gain is unclear and may be related to the lack of effective therapeutic options post second-line, or that 6-month PFS rate is not a good surrogate marker of OS in patients with PD-EP-NEC. No patients discontinued treatment due to Coronavirus-19 and the impact of this on the outcome measures was considered negligible.

However, the OS in the liposomal irinotecan arm of NET-02 was similar to that reported (6.2 months) in a recent national retrospective multicentre study (France) of 121 patients receiving second-line treatment for advanced poorly differentiated NEC of lung, gastro-entero-pancreatic, unknown or “other” primary site origin.^31^ The patients recruited into NET-02 predominantly had tumours with a Ki-67 ≥ 55% and were platinum-resistant, reflecting an aggressive histology and is likely representative of patients seen in daily clinical practice. These are a challenging population to treat, and so the results of NET-02 are encouraging in this poor prognosis group, and do provide some efficacy, safety and quality-of-life data for future trial development in this setting, but also highlight the difficulties in improving survival in this disease group.

Approximately one half of patients did not complete a quality-of-life questionnaire post baseline in NET-02. This is a recognised occurrence in clinical trials,^32^ and reflects issues with compliance and a poor prognosis disease where patients can deteriorate rapidly and are not able, or decline, completion of subsequent questionnaires. It also highlights the limitations of the available tools for assessing quality-of-life in oncology clinical trials.

Randomised controlled trials that report early have been suggested to overestimate the effect size.^33^ However, when 50% of the required information has been accrued,^33^ reporting early has been suggested to have a negligible effect on estimated effect sizes.^34^ This study was recommended for closure by the DSMB when 26 and 27 evaluable patients had reached 6 months, equating to 51% and 53% of information accrued. This suggests that the effect size is reasonable to report as phase II evidence, but these findings need to be interpreted with caution.

There are other limitations associated with NET-02, including that patients were recruited from the UK alone, but the efficacy results are not dissimilar to previously reported retrospective studies (multiple locations)^10^ and the prospective French PRODIGE 41-BEVANEC trial.^14^ Pathology review was carried out regionally (not centrally), acknowledging the challenges in morphological classification in particular, and with review carried out on biopsy rather than resection samples, with the potential for misdiagnosis, but centres recruiting to the trial were predominantly ENETS Centres of Excellence (79%) following defined standard pathological guidelines.^35^ There are no standard definitions of platinum sensitivity/resistance in patients with PD-EP-NEC and so re-challenge with previous platinum-based treatment may be appropriate in select patients, and may even be reasonable after 3 months, but prospective data is lacking.^31^ There are also limitations in interpretation of the quality-of-life data due to non-completion of questionnaires post baseline, and so the overall level of quality-of-life reported may not be completely reflective of the entire population recruited to the NET-02 trial. However, despite this challenge, there is a paucity, if not non-existence, of quality-of-life data from prospective randomised studies in this setting and so these data are valuable and add to the available limited literature.

Randomised trials in this disease group and setting are extremely rare, and the results of this trial will be informative for future trial design and highlights that conduct of randomised studies in this disease group of unmet need is possible and safe. The worse 6-month PFS rate and more unfavourable quality-of-life in the docetaxel arm would dissuade one from further use in this indication, unless translational findings identify a select population that may derive benefit. There was no association between baseline NSE and PFS status at 6 months post randomisation in the NET-02 trial, but this may be a reflection of the patient numbers included in this study, and larger prospective studies may be required to confirm these findings. In NET-02, the GI-NEC score identified a better and poor prognostic group in all patients and in the nal-IRI/5-FU/folinic acid arm, and could be used as a stratification factor in future trials using this therapeutic combination. This was also concordant with the findings from the French PRODIGE 41-BEVANEC randomised phase II study where those with a poor prognostic GI-NEC score had worse OS.^14^ The tolerable safety profile of nal-IRI/5-FU/folinic acid may also lend itself to future therapeutic combinations, acting as a chemotherapeutic back bone, potentially in the first-line advanced setting and potentially combined with immunotherapy, with OS as the primary end-point. Translational analysis may also provide the foundations for future potential targeted exploitation, as to date the observed genomic differences in patients with PD-EP-NEC have not been meaningfully used in prospective randomised or non-randomised trials and treatment in the standard clinical setting remains chemotherapy, unless perhaps in the rare circumstances where tumour agnostic therapies are approved (e.g. targeting neurotrophic tyrosine receptor kinase, microsatellite instability, BRAF V600E).^36^^,^^37^ Future research may reveal variable outcomes in response to one-size-fits-all chemotherapy based on genomic differences across NEC subgroups.

In conclusion, the NET-02 trial provides prospective efficacy, toxicity and quality-of-life data in the second-line setting in a disease group of unmet need, and represents some of the strongest evidence available to recommend systemic treatment to these patients. These results also highlight the challenges in improving survival for these patients and that conduct of randomised trials in this disease group is possible and safe.

## Contributors

**Conception and design**: Mairéad G McNamara and Juan W Valle, with statistical input from Zoe Craig and David Cairns.

**Provision of study materials or patients**: All authors.

**Collections and assembly of data**: All authors.

**Data analysis and interpretation**: Statistical analysis was performed by Zoe Craig and David Cairns and data was interpreted by all authors.

**Manuscript writing**: All authors.

**Final approval of manuscript**: All authors.

**Accountable for all aspects of the work**: All authors. MMN, ZC, JS, and DC accessed and verified all the data. All authors had full access to the data in the study and had final responsibility for the decision to submit the manuscript. All authors approved the manuscript for submission and vouch for the accuracy and completeness of the data and for fidelity of the trial to the protocol. All authors are from academic teams.

## Data sharing statement

Deidentified patient data may be requested from Leeds Cancer Research UK Clinical Trials Unit if data are not publicly available. Requests to access data should be made to CTRU-DataAccess@leeds.ac.uk in the first instance. A formal review process includes verifying the availability of data, conducting a review of any existing agreements that may have implications for the project, and ensuring any transfer is in compliance with the ethics approval. The relevant investigator will be required to sign a data release form prior to transfer.

## Declaration of interests

Mairéad G McNamara: has received research grant support from Servier, Ipsen, NuCana and Astra Zeneca. She has received travel and accommodation support from Advanced Accelerator Applications (UK and Ireland) Ltd, and Ipsen, and speaker honoraria from Advanced Accelerator Applications (UK and Ireland) Ltd.. She has served on advisory boards for Incyte and Astra Zeneca.

Jayne Swain: reports grants and non-financial support from Servier, during the conduct of the study.

Zoe Craig: reports grants and non-financial support from Servier, during the conduct of the study.

Rohini Sharma: has received grant support from AAA, Incyte, Boston Scientific, Bayer and Terumo. She has received speaker honoraria from Roche and Esai.

Olusola Faluyi: no conflicts of interest to declare.

Jonathan Wadsley: reports grants and personal fees from AstraZeneca, grants and personal fees from Sanofi-Genzyme, speaker and advisory board honoraria from Astrazeneca, Celgene, Novartis, Ipsen, Roche, Bayer, Esteve, Lilly and Eisai.

Carys Morgan: no conflicts of interest to declare.

Lucy R Wall: no conflicts of interest to declare.

Ian Chau: reports advisory role for Eli-Lilly, Bristol Meyers Squibb, MSD, Bayer, Roche, Merck-Serono, Five Prime Therapeutics, AstraZeneca, Oncologie International, Pierre Fabre; research funding from Eli-Lilly, Janssen-Cilag, Sanofi Oncology, Merck-Serono; honorarium from Eli-Lilly.

Nick Reed: no conflicts of interest to declare.

Debashis Sarker: reports personal fees from MSD, personal fees and non-financial support from EISAI, personal fees and non-financial support from Ipsen, personal fees from Bayer, non-financial support from Mina Therapeutics, personal fees from Pfizer, personal fees from Novartis.

Jane Margetts: no conflicts of interest to declare.

Daniel Krell: no conflicts of interest to declare.

Judith Cave: reports educational support from Amgen.

Sharmila Sothi: no conflicts of interest to declare.

Alan Anthoney: no conflicts of interest to declare.

Chris Bell: no conflicts of interest to declare.

Alkesh Patel: no conflicts of interest to declare.

Jamie B Oughton: reports grants and/or non-financial support from Servier, Roche, AstraZeneca, Pfizer and Bayer.

David Cairns: reports grants and non-financial support from Servier, during the conduct of the study.

Wasat Mansoor: no conflicts of interest to declare.

Angela Lamarca: has received travel and educational support from Ipsen, Pfizer, Bayer, AAA, SirtEx, Novartis, Mylan and Delcath; speaker honoraria from Merck, Pfizer, Ipsen, Incyte, AAA, QED and Servier; advisory honoraria from EISAI, Nutricia Ipsen, QED, Roche and Servier; she is a member of the Knowledge Network and NET Connect Initiatives funded by Ipsen.

Richard A Hubner: has served on the advisory boards for Roche, BMS, Eisai, Celgene, Beigene, Ipsen and BTG. He has received speaker fees from Eisai, Ipsen, Mylan, PrimeOncology and has received travel and educational support from Bayer, BMS and Roche.

Juan W Valle: has had consulting or advisory roles for Agios, AstraZeneca, Delcath Systems, Keocyt, Genoscience Pharma, Incyte, Ipsen, Merck, Mundipharma EDO, Novartis, PCI Biotech, Pfizer, Pieris Pharmaceuticals, QED and Wren Laboratories. He is on speakers’ bureaus for Imaging Equipment Limited, Ipsen, Novartis and Nucana; and has received travel grants from Celgene and Nucana.

## Appendix A Supplementary data

Supplementary Table S1

2b_NET-02_Protocol_v6.0_200603_clean

Captions for supplementary material
