# Supplementary material for: NET-02: a randomised, non-comparative, phase II trial of nal-IRI/5-FU or docetaxel as second-line therapy in patients with progressive poorly differentiated extra-pulmonary neuroendocrine carcinoma
Source: eClinicalMedicine. 2023 Jun 2;60:102015. doi: 10.1016/j.eclinm.2023.102015 (PMC10242623; doi:10.1016/j.eclinm.2023.102015)
Supplement: Supplementary Table S1 [file mmc1.docx]

**Table S1**: Patterns of change in C30 symptom items and GINET21 items from baseline

| **C30 symptoms** | **Nal-Iri** | **Docetaxel** | **GINET21** | **Nal-Iri** | **Docetaxel** |
| --- | --- | --- | --- | --- | --- |
| Appetite loss | − | − | Body image | ↑ | ↑^*^ |
| Constipation | ↓ | ↓^*^ | Disease-related worries | ↑ | ↑^*^ |
| Diarrhoea | ↓ | ↓^*^ | Endocrine symptoms | ↓^*^ | ↓^*^ |
| Dyspnoea | − | ↑^*^ | GI symptoms | − | − |
| Fatigue | − | ↓ | Information | ↑ | ↑^*^ |
| Financial problems | ↑ | ↓^*^ | Muscle/Bone pain | ↑^*^, ↓^*^ | ↑^*^ |
| Insomnia | ↑ | ↓^*^ | Sexual function | ↑^*^ | ↑^*^ |
| Nausea/Vomiting | ↓^*^ | ↓ | Social function | ↑ | ↑ |
| Pain | ↑ | − | Treatment-related symptoms | ↑^*^ | ↓ |

**Nal-Iri**: liposomal irinotecan, “Information”: Have you had problems receiving adequate information about your disease and treatment; ↑ improvement (>10 point change from baseline at ≥2 time points); ↓ worsening (>10 point change from baseline at ≥2 time points); * indicates improvement/worsening for one time point only; − stable (change from baseline <10 points across all time points). Nal-IRI change from baseline to Week 30. Docetaxel change from baseline to Week 18.
