# Supplementary material for: NET-02: a randomised, non-comparative, phase II trial of nal-IRI/5-FU or docetaxel as second-line therapy in patients with progressive poorly differentiated extra-pulmonary neuroendocrine carcinoma
Source: eClinicalMedicine. 2023 Jun 2;60:102015. doi: 10.1016/j.eclinm.2023.102015 (PMC10242623; doi:10.1016/j.eclinm.2023.102015)
Supplement: 2b_NET-02_Protocol_v6.0_200603_clean [file mmc2.pdf]

NET-02

Second-line therapy for patients with progressive poorly differentiated extra-pulmonary neuroendocrine carcinoma

A multi-centre, randomised, parallel group, open-label, phase II, single-stage selection trial of liposomal irinotecan (nal-IRI) and 5-fluorouracil (5-FU)/folinic acid or docetaxel as second-line therapy in patients with progressive poorly differentiated extra-pulmonary neuroendocrine carcinoma (NEC))

Version 6.0, 3rd June 2020

EudraCT Number: 2017-002453-11
ISRCTN Number: 10996604
REC Reference: 18/NW/0031
IRAS: 230387
Sponsor Reference: CFTSp116

Sponsor: The Christie NHS Foundation Trust

Funded by: Servier (Grant Ref: 016-34263)

Chief Investigator: Dr Mairéad G McNamara
Senior Lecturer/Honorary Consultant in Medical Oncology
Division of Cancer Sciences, The University of Manchester & The Christie NHS Foundation Trust, Wilmslow Road, Manchester, M20 4BX

Under the auspices of the neuroendocrine tumour subgroup of the National Cancer Research Institute (NCRI) Upper Gastrointestinal (GI) Clinical Studies Group

The Sponsor and Clinical Trials Research Unit (CTRU) accept no responsibility for the accuracy of content reproduced from this protocol and incorporated into additional documentation developed by collaborating or third party organisations.

Key Contacts

**General Enquiries:**	Email: net-02@leeds.ac.uk
**Registration/Randomisation:**	Phone : 0113 343 2290<br>Web : https://lictr.leeds.ac.uk/webrand/
**Pharmacovigilance:**	Fax: 0113 343 0686<br>Email: net-02@leeds.ac.uk

Role in trial	Name and position	Contact details
**Chief investigator (CI)**	**Dr Mairéad G McNamara**<br>Senior Lecturer/Honorary Consultant in Medical Oncology	Division of Cancer Sciences, The University of Manchester & The Christie NHS Foundation Trust, Wilmslow Road, Manchester, M20 4BX<br>**Telephone |** 0161 446 8106<br>**Fax |** 0161 446 3468<br>**Email |** Mairead.McNamara@christie.nhs.uk
**Co-investigator (Co-I)**	**Professor Juan W Valle**<br>Consultant/Honorary Professor in Medical Oncology	Division of Cancer Sciences, The University of Manchester & The Christie NHS Foundation Trust, Wilmslow Road, Manchester, M20 4BX<br>**Telephone |** 0161 446 8106<br>**Fax |** 0161 446 3468<br>**Email |** Juan.Valle@christie.nhs.uk
**Sponsor's representative**	**Mr Wes Dale**<br>Managing Director of Research, Education and Innovation	The Christie NHS Foundation Trust, The Oglesby Cancer Research Building, 555 Wilmslow Road, Manchester, M20 4GJ<br>**Telephone |** 0161 918 7902<br>**Email |** wes.dale@christie.nhs.uk
**CTRU Delivery Lead**	**Helen Howard**<br>Head of Trial Management - Solid Tumours	Clinical Trials Research Unit (CTRU), University of Leeds<br>**Telephone |**0113 343 4242<br>**Fax |** 0113 343 0686<br>**Email |** h.howard@leeds.ac.uk
**Trial and Data Management**	**Jayne Swain**<br>Senior Trial Manager<br><br>**Emma Batman**<br>Trial Coordinator	Clinical Trials Research Unit (CTRU), University of Leeds<br>**Telephone | 0113 343 4108**<br>**Fax |** 0113 343 0686<br>**Email |** j.swain@leeds.ac.uk<br><br>**Telephone |** 0113 343 3090<br>**Fax |** 0113 343 0686<br>**Email |**e.e.batman@leeds.ac.uk
**Statistics**	**Zoe Craig**<br>Medical Statistician<br><br>**David Cairns**	Clinical Trials Research Unit (CTRU), University of Leeds **Telephone |** 0113 343 8367<br>**Email |** z.craig@leeds.ac.uk<br><br>**Telephone |** 0113 343 1712

	Associate Professor of Statistics	**Email |** D.A.Cairns@leeds.ac.uk
**Lead pharmacist**	**Alkesh Patel**<br>Leadclinical trials pharmacist	The Christie NHS Foundation Trust, Wilmslow Road, Manchester, M20 4BX<br>**Telephone |** 0161 918 7651<br>**Email |** alkesh.patel@christie.nhs.uk
**Clinical Advisors**	**Dr Olusola Faluyi**<br>Consultant Medical Oncologist<br><br>**Professor Tim Meyer**<br>Consultant Medical Oncologist<br><br>**Dr Debashis Sarker**<br>Consultant Medical Oncologist	The Clatterbridge Cancer Centre, Liverpool<br><br><br>UCL Cancer Institute, London<br><br><br>Kings College London
**Patient Advisor**	Carole Beckett	c/o net-02@leeds.ac.uk

Disclaimer

This document describes the NET-02 trial and provides information about procedures for entering patients into it. The protocol should not be used as a guide for the treatment of patients outside the trial. Every care was taken in drafting this protocol, but corrections or amendments may be necessary. These will be circulated to all Investigators participating in the trial. Centres entering patients for the first time are advised to contact CTRU to confirm that they have the most recent and approved version of this protocol.

Acknowledgements

Thanks to Dr Alison Backen (Project Manager, The University of Manchester & The Christie NHS Foundation Trust) for her contribution to protocol development.

Contents

Key Contacts 2
2. Trial Summary 8
3. Trial Schema 12
4. Abbreviations 13
5. Background 15
5.1 Neuroendocrine neoplasms 15
5.2 Current second-line treatment options for patients with neuroendocrine carcinomas 15
5.3 Study summary 15
5.4 Liposomal irinotecan (Nal-IRI) 17
5.4.1 Nal-IRI mechanism of action 17
5.4.2 Nal-IRI pre-clinical experience 17
5.4.3 Nal-IRI pre-clinical pharmacokinetics 17
5.4.4 Clinical experience with nal-IRI 18
5.4.5 Nal-IRI pharmacokinetics in humans 21
5.4.6 Nal-IRI safety in humans 21
5.4.7 Interaction with other medicinal products 22
5.5 Rationale for the Use of nal-IRI in combination with 5-FU and folinic acid 23
5.5.1 Efficacy of irinotecan in combination with 5-FU and folinic acid 24
5.5.2 Efficacy of nal-IRI in combination with 5-FU and folinic acid 25
5.5.3 Safety data for the combination of Nal-IRI, 5-FU and folinic acid 25
5.6 Rationale for the use of docetaxel 27
5.7 Study setting 27
6. Aims and Objectives 27
6.1 Aim 27
6.2 Trial objectives 27
6.2.1 Primary objective 27
6.2.2 Secondary objectives 28
6.2.3 Samples for research 28
7. Design 28
8. Eligibility 29
8.1 Inclusion criteria 29
8.2 Exclusion criteria 30
9. Recruitment Process 31
9.1 Recruitment Setting 31
9.2 Eligibility Screening 31
9.3 Informed Consent and Eligibility 31
9.3.1 Initial Information and Initial Approach 32
9.3.2 Consent Process 32
9.4 Registration and randomisation procedures 33

9.6.1 Registration ... 33
9.6.2 Post-registration actions ... 34
9.6.3 Randomisation ... 34
9.6.4 Treatment Allocation ... 35
9.6.5 Post-randomisation actions ... 36
10. Trial Medicinal Product Management ... 36
10.1 Liposomal Irinotecan Hydrochloride (nal-IRI) ... 36
10.1.1 Composition of nal-IRI ... 36
10.1.2 Supply, distribution and handling of nal-IRI ... 37
10.2 5-Flurouracil (5-FU) and folinic acid ... 38
10.2.1 Composition of 5-FU and folinic acid ... 38
10.2.2 Supply, distribution, and handling of 5-FU and folinic acid ... 38
10.3 Docetaxel ... 38
10.3.1 Composition of docetaxel ... 38
10.3.2 Supply, storage and handling of docetaxel ... 38
10.4 Granulocyte-Colony Stimulating Factor (G-CSF) ... 39
10.4.1 Composition of G-CSF ... 39
10.4.2 Supply, storage and handling of G-CSF ... 39
11. Treatment Details ... 39
11.1 Pre-treatment investigations and tests required ... 39
11.2 Nal-IRI/5-FU/folinic acid Treatment ... 39
11.2.1 Nal-IRI doses and administration ... 39
11.2.2 Nal-IRI premedication ... 40
11.2.3 5-FU and folinic acid doses and administration ... 40
11.3 Docetaxel treatment ... 40
11.3.1 Docetaxel doses and administration ... 41
11.4 Treatment toxicities ... 41
11.4.1 Nal-IRI toxicity ... 41
11.4.2 Nal-IRI, 5-FU and folinic acid combination therapy – potential toxicity ... 44
11.4.3 Docetaxel toxicity ... 44
11.5 Concomitant Therapies ... 45
11.5.1 Interactions affecting the use of nal-IRI ... 45
11.5.2 Interactions of nal-IRI affecting the use of other drugs ... 46
11.5.3 Interactions affecting the use of 5-FU and/or folinic acid ... 46
11.5.4 Interactions affecting the use of docetaxel ... 46
11.6 Dose modification requirements ... 47
11.6.1 Nal-IRI dose modifications ... 47
11.6.2 5-FU and folinic acid dose modifications ... 49
11.6.3 Docetaxel dose modifications ... 51
11.6.5 Management of infusion reactions ... 52

11.7 Withdrawals ... 53
12. Assessments, Samples and Data Collection ... 53
12.1 Schedule of Events ... 54
12.2 Eligibility and baseline assessments ... 57
12.3 Treatment cycle assessments ... 58
12.3.2 Assessments at cycle 1 ... 59
12.3.2 Assessments at cycle 2 and onwards ... 59
12.3.3 Assessments at 6-weekly timepoints ... 59
12.3.4 Assessments at 8 weekly timepoints ... 60
12.4 Assessments at disease progression ... 60
12.5 Assessment following end of the trial interventional phase ... 60
12.6 End of Trial Treatment ... 61
12.7 Disease progression ... 61
12.8 Quality of Life ... 62
12.9 Blood sample analysis ... 62
12.10 Adverse Events and Serious Adverse Events ... 63
12.11 Pregnancies ... 63
12.12 Deaths ... 64
12.13 End of trial ... 64
12.14 Trial data and documentation held at sites ... 64
12.15 Protocol deviations and violations ... 65
13. Safety Monitoring Plan ... 65
14. Pharmacovigilance ... 65
14.1 General Definitions ... 65
14.2 Reporting Requirements ... 66
14.3 Responsibilities ... 69
15. Participant Questionnaires ... 70
15. Samples for Research ... 71
16. Endpoints ... 71
16.1 Primary endpoint ... 71
16.2 Secondary endpoints ... 71
16.3 Samples for research ... 72
17. Statistical Considerations ... 72
17.1 Sample size calculation ... 72
17.2 Efficacy ... 72
17.3 Treatment Selection ... 73
17.4 The recruitment rate ... 73
18. Statistical Analysis ... 73
18.1 General considerations ... 73
18.2 Frequency of analyses ... 74

18.3 Primary endpoint analysis 74
18.4 Secondary endpoint analysis 74
19. Trial Monitoring 75
19.1 Data Monitoring and Ethics Committee (DMEC) 75
19.2 Data monitoring 75
19.3 Clinical governance issues 75
20. Quality Assurance and ethical considerations 75
20.1 Quality Assurance 75
20.2 Serious breaches 76
20.3 Ethical and regulatory considerations 76
21. Confidentiality 76
21.1 Trial record retention and archiving 77
22. Sponsorship and indemnity 77
23. Study Organisational Structure 78
23.1 Individuals and Individual Organisations 78
24. Publication Policy 79
25. References 80
26. Appendices 83
Appendix 1 - Response Evaluation Criteria In Solid Tumours (RECIST) 83
Appendix 2 - Eastern cooperative oncology group (ECOG) performance status (PS) 84
Appendix 3 - Cockcroft-Gault Equation 85
Appendix 4 – Management of nal-IRI/5-FU/folinic acid toxicities (as per local standard practice) 86
Appendix 5 – Management of docetaxel toxicities (as per local standard practice) 88
Appendix 6 - Proposed algorithm for diarrhoea management, but local standard practice may be used as an alternative 90
Appendix 7 - Management of infusion reactions 91
Appendix 8 – Inhibitors and Inducers of CYP3A 93
Appendix 9 – Safety Monitoring Plan 95

2. Trial Summary

**Title**	A multi-centre, randomised, parallel group, open-label, phase II, single-stage selection trial of liposomal irinotecan (nal-IRI) and 5-fluorouracil (5-FU)/folinic acid or docetaxel as second-line therapy in patients with progressive poorly differentiated extra-pulmonary neuroendocrine carcinoma (NEC)
**Acronym**	NET-02
**Background**	As there is no standard treatment beyond first-line etoposide/platinum-based chemotherapy in patients with progressive poorly differentiated extra-pulmonary neuroendocrine carcinoma (NEC), this is an area of unmet need in this disease group.<br><br>Combination regimens such as irinotecan/5-FU are a second-line treatment option currently used in Europe and world-wide, without trial evidence, for this subset of patients and is more often recommended for those patients with a NEC with a Ki-67≥55%, with temozolomide based-combinations being preferred for those with Ki-67<55%.<br><br>In devising treatment strategies for extra-pulmonary NEC, many refer to the extensive literature on high-grade NEC of the lung of which docetaxel is a second-line therapy option.
**Design**	A multi-centre, randomised, parallel group, open-label, phase II, single-stage selection trial of liposomal irinotecan (nal-IRI)/5-fluorouracil/folinic acid or docetaxel as second-line therapy in patients with progressive poorly differentiated extra-pulmonary neuroendocrine carcinoma, with the overall aim of selecting a treatment for continuation to a phase III trial.
**Objective**	To assess the efficacy of nal-IRI/5- fluorouracil /folinic acid or docetaxel separately, as second-line therapy in patients with progressive poorly differentiated extra-pulmonary NEC, with selection criteria applied to establish which treatment to take forward into a phase III trial.

**Endpoints**	**Primary endpoint**<br>• 6-month progression-free survival (PFS) rate<br><br>**Secondary endpoints**<br>• Overall survival (OS)<br>• Objective response rate (ORR) using Response Evaluation Criteria in Solid Tumours (RECIST) version 1.1[1]<br>• Toxicity<br>• Quality of life (QoL) using European Organisation for Research and Treatment of Cancer (EORTC) quality of life (QLQ) validated questionnaires C30 and NET21<br>• Serum concentration of neuron-specific enolase (NSE)<br><br>If both treatment arms exceed the required level of efficacy to warrant further evaluation in a phase III trial, the treatment with the higher PFS rate at 6 months will be selected. However if the difference in 6 month PFS rates between the treatment arms is less than 5%, alternative selection criteria such as toxicity rates or quality of life may be considered in addition to PFS rate.<br><br>**Samples for research**<br><br>• Quantification of circulating tumour cells at baseline, 6 weeks and on progression, to identify any correlation with disease-related outcomes.<br>• Quantification of circulating tumour deoxyribonucleic acid (DNA) at baseline, 6 weeks and on progression, to identify any correlation with disease-related outcomes.<br>• Molecular profiling of circulating tumour cells, circulating tumour DNA and tumour tissue (further immunohistochemistry on tumour tissue may also be required) to identify any correlation with disease-related outcomes.<br>• Potential generation of mouse models of neuroendocrine carcinoma.
**Recruitment**	This study plans to recruit patients from approximately 16 UK centres over a 37 month duration.
**Population (Please refer to section 8 for full eligibility criteria)**	**Key Inclusion criteria**<br>• Diagnosed with poorly differentiated (as defined by the World Health Organisation in 2019, Ki 67 >20%) extra-pulmonary neuroendocrine carcinoma (NEC grade 3, confirmed by histology).<br>• Prior treatment with first-line platinum-based chemotherapy and ≥28 days from Day 1 of the previous treatment cycle.<br>• Documented radiological evidence of disease progression <u>OR</u> discontinuation of first-line platinum-based chemotherapy due to intolerance.

	• Measurable disease according to RECIST 1.1[1].<br>• Eastern Co-operative Oncology Group (ECOG) performance status (PS) of ≤2. This is defined in Appendix 2.<br>• Adequate renal, haematological and liver function.<br>• Age ≥18 years and have a life expectancy >3 months.<br>• Patients must have given written informed consent.<br><br>**Key Exclusion criteria**<br>• Known or suspected allergy or hypersensitivity reaction to any of the components of study treatment or their excipients.<br>• Previous treatment (for neuroendocrine carcinoma) with any of the components of combination chemotherapy regimens detailed in this study (nal-IRI or 5-FU or irinotecan or topoisomerase inhibitors or taxane-based therapy).<br>• Use (including self-medication) within one week of randomisation and for the duration of the study of any of the following: St. John's wort, grapefruit, Seville oranges, medicines known to inhibit UGT1A1 and agents and medicines known to inhibit or induce either CYP3A4 or CYP3A5 (see Appendix 8 for list).<br>• Any evidence of severe or uncontrolled systemic diseases which, in the view of the treating clinician, makes it undesirable for the patient to participate in the trial.<br>• Have a medical or psychiatric condition that impairs the patient's ability to give informed consent.
**Dose**	This study will recruit 102 patients in total, randomised on a 1:1 basis to receive either;<br><br>**Nal-IRI/5-FU/folinic acid |** nal-IRI (70mg/m^2 intravenously [IV] over 90 minutes (± 10 minutes)) prior to 5-FU (5-FU 2400 mg /m^2 BSA infusor over 46 hours) and racemic folinic acid (as per local standard practice) every 14 days.<br><br>**Docetaxel |** Docetaxel (75mg/m^2 IV over 60 minutes) and G-CSF (as per local standard practice) every 21 days.
**Duration**	Participants will be treated for a minimum of 6 months. Trial treatment will continue until progressive disease, intolerable toxicity, delay of treatment for more than 28 days, development of any condition or occurrence of any event, which, in the opinion of the local investigator, justifies discontinuation of treatment, patient request or until 6 months after the last participant is randomised, whichever comes first.
**Evaluation of outcome measures**	• Patients will undergo tumour evaluation by CT scan at baseline, and then every 8 weeks (± 7 days) until either progression, death or until 6 months after the last participant is randomised, whichever comes first.<br>• Patient-reported quality of life questionnaires (QoL) will be assessed using the EORTC QLQ-C30 in combination with the QLQ-GINET-21. QoL will be completed at the point of

	randomisation and then at the start of every second treatment cycle (docetaxel)/third treatment cycle (nal-IRI) from treatment start date until either progression, death or until 6 months after the last participant is randomised, whichever comes first.<br>• Adverse events or toxicities will be collected at every treatment cycle from randomisation until 30 days post cessation of trial treatment.<br>• Blood samples will be taken at baseline, treatment start, 6-weekly thereafter and at disease progression to measure neuron-specific enolase for assessment as a potential biomarker of response to treatment.<br>• Blood samples will be taken at baseline, 6 weeks after treatment start and on disease progression for quantification and molecular characterisation of circulating tumour cells and tumour DNA to identify any correlation with disease-related outcomes.
**Registration /Randomisation**	Registration of all potential participants prior to conducting trial-specific investigations to confirm eligibility, followed by randomisation for eligible patients on a 1:1 basis to receive either nal-IRI (in combination with 5-FU/folinic acid) or docetaxel. Randomisation to be performed by the Clinical Trials Research Unit (CTRU), Leeds.

3. Trial Schema

Figure 1 | Trial schema

Population: adults with progressive poorly differentiated extra-pulmonary neuroendocrine carcinoma (NEC grade 3)

Key Inclusion criteria: Prior treatment with first-line platinum-based chemotherapy and ≥28 days from Day 1 of the previous treatment cycle, documented radiological evidence of disease progression OR discontinuation of first-line platinum-based chemotherapy due to intolerance, measurable disease according to RECIST, ECOG performance status ≤2, adequate renal, haematological and liver function, age ≥18 years, life expectancy >3 months, able to give written informed consent.

Key Exclusion criteria: known or suspected allergy or hypersensitivity reaction to any of the study treatments, previous treatment with nal-IRI, 5-FU, irinotecan, topoisomerase inhibitors or taxane-based therapy, use within 1 week and during study duration of CYP3A4 and CYP3A5 inhibitors or inducers and UGT1A1 inhibitors

↓

Written Informed Consent and REGISTRATION
(all patients consented MUST be registered)

↓

Formal eligibility and pre-randomisation assessment:
Pregnancy test for women of childbearing potential, Electrocardiogram (ECG)
Quality of Life (QoL): EORTC QLQ-C30, EORTC QLQ-GI.NET21

↓

RANDOMISATION (1:1)
Minimisation incorporating a random element, stratified by: hospital site, Ki-67 marker, ECOG performance status, presence of liver metases, response to first-line platinum-based chemotherapy
Post-randomisation assessments: blood samples and archival paraffin-embedded tissue for translational research
Baseline CT scan must be carried out ≤28 days prior to starting treatment

Nanoliposomal Irinotecan/5-Flourouracil (5-FU) and folinic acid (n=51)

nal-IRI (70mg/m² IV over 90 minutes) followed by folinic acid (as per local standard practice), followed by 5-FU (2400 mg /m² BSA intravenously over 46 hours) every 14 days (+3 days/-1 day) until progression, toxicity or ≥28 day delay

Full blood count, urea and electrolytes and liver function tests ≤3 days of day 1 of each cycle

CT or MRI scan every 8 weeks (±7 days) until disease progression

QoL: day 1 of cycles 3, 5, 7, 9, etc (every 6 weeks (±7 days) until disease progression)

Blood sample at start of treatment, 6 weekly thereafter and on progression for serum enolase measurement; at start of treatment, at 6 weeks and on progression for translational research

Docetaxel (n=51)

Docetaxel (75mg/m² IV over 60 minutes) and G-CSF (as per local standard practice) every 21 days (+3 days/-1 day) until progression, toxicity or ≥28 day delay

Full blood count, urea and electrolytes and liver function tests ≤3 days of day 1 of each cycle

CT or MRI scan every 8 weeks (±7 days) until disease progression

QoL: day 1 of cycles 2, 4, 6, 8, etc (every 6 weeks (±7 days) until disease progression)

Blood sample at start of treatment, 6 weekly thereafter and on progression for serum enolase measurement; at start of treatment, at 6 weeks and on progression for translational research

↓

Toxicity assessment: 28 days (+7 days) post end of treatment (telephone call or clinic visit as clinically appropriate)

4. Abbreviations

5-FU	5-fluorouracil
AE	Adverse event
ALP	Alkaline phosphatase
ALT	Alanine aminotransferase
AR	Adverse reaction
AST	Aspartate aminotransferase
BCT	Blood collection tube
BSA	Body surface area
CAIX	Carbonic anhydrase IX
CBC	Complete blood count
CI	Chief Investigator
Co-I	Co-investigator
CrCl	Creatinine clearance
CRF	Case report form
CT	Computerised tomography
CTC	Common toxicity criteria
CTCAE	Common terminology criteria for adverse events
CTRU	Clinical Trials Research Unit
DLT	Dose limiting toxicities
DNA	Deoxyribonucleic acid
dL	Decilitres
DMEC	Data monitoring and ethics committee
DSUR	Development safety update report
ECG	Electrocardiogram
ECOG	Eastern Cooperative Oncology Group
EMA	European Medicines Agency
EORTC	European Organisation for Research and Treatment of Cancer
ET	End of treatment
EudraCT	European clinical trials database
FBC	Full blood count
FOLFOX	Combination of 5-fluorouracil (5-FU) and oxaliplatin
mFOLFOX	Modified combination of 5-FU and oxaliplatin
G1, 2 or 3	Grade 1, 2 or 3
GCP	Good clinical practice
GCSF	Granulocyte colony stimulating factor
GFR	Glomerular filtration rate
GP	General Practitioner
Hb	Haemoglobin
HR	Hazard ratio
HZT	Heterozygous
IM	Intramuscular injection
IMP	Investigational Medicinal Product
ISF	Investigator Site File
IV	Intravenous
Kg	Kilogram

L	Litre
LDH	Lactate dehydrogenase
LV	Leucovorin/Folinic acid
m	metre
Mg	Milligrams
MHRA	Medicines and Healthcare products Regulatory Agency
ml	Millilitres
MRI	Magnetic Resonance Imaging
mRNA	Messenger ribonucleic acid
MTD	Maximum tolerated dose
nal-IRI	liposomal irinotecan
NCCN	National Comprehensive Cancer Network
NEC	Neuroendocrine carcinoma
NET	Neuroendocrine tumour
NSE	Neuron-specific enolase
NYHA	New York Heart Association
ORR	Objective Response Rate
OS	Overall survival
PD	Progressive disease
PFS	Progression-free survival
PI	Principal Investigator
PIN	Personal identification number
PK	Pharmacokinetics
PS	Performance Status
PT	Pro-thrombin time
QoL	Quality of life
QTc	Corrected QT interval
REC	Research Ethics Committee
RECISTv1.1	Response Evaluation Criteria In Solid Tumours version 1.1
RT	Radiotherapy
R&D	Research and development
SAR	Serious Adverse Reaction
SAE	Serious Adverse Event
SAP	Statistical analysis plan
SD	Stable disease
SmPC	Summary of product characteristics
SN-38	The 100-1000-fold more active metabolite of irinotecan (which is a pro-drug)
SSOP	Study site operating procedure
SUSAR	Suspected Unexpected Serious Adverse Reaction
TID	Three times daily
TMG	Trial Management Group
TSC	Trial Steering Committee
UDP	Uridine diphosphate
ULN	Upper Limits of Normal
WBC	White blood count
WHO	World Health Organisation
WT	Wild type

5. Background

5.1 Neuroendocrine neoplasms

The incidence of neuroendocrine tumours (NETs) in Europe ranges from 0.6 to 4.9 per 100,000.[2 3] Extra-pulmonary, poorly differentiated neuroendocrine carcinomas (NECs), previously defined as poorly differentiated endocrine carcinoma, are rare and represent approximately 5-10% of digestive neuroendocrine tumours.[4 5]

These tumours are characterised by aggressive histological features; high Ki-67 index (>20% by definition, but usually higher (>75%),[6] extensive necrosis, and nuclear atypia, and are classified as NEC grade 3 according to the World Health Organisation (WHO) 2019 classification.[7 8 9]

Gastrointestinal NECs mainly arise from the oesophagus, stomach, pancreas and colon[10-12] and encompass two histopathological entities: small cell NEC and large cell NEC.[13 14]

First-line treatment for extra-pulmonary NECs has largely remained unchanged since a study in the early 1990s reported that the etoposide-platinum combination produced anti-tumoural activity and high tumour response rates (41-67%).[15] Relapse invariably occurs in patients during or following completion of first-line therapy and there is no established second-line treatment for patients with poorly differentiated NECs.

5.2 Current second-line treatment options for patients with neuroendocrine carcinomas

A number of small retrospective series have published results of the outcomes of second-line chemotherapy after failure of the etoposide-platinum combination in patients with grade 3 NECs.[16-22]

In the NORDIC NEC study that reported predictive and prognostic factors for treatment and survival in 305 patients with advanced gastrointestinal NEC (WHO grade 3), second-line chemotherapy was administered to 100 patients. Of these, 35 received temozolomide-based chemotherapy and 20 received docetaxel-containing regimens.[16] The response rate after second-line chemotherapy for 84 evaluable patients was 18%.

In this study, 47% of patients had tumours that had a Ki-67<55% and 53% had a Ki-67≥55%. Those whose tumours had a Ki-67<55% had a lower response rate (15% versus 42%, p<0.001), but better survival than patients whose tumours cells had a Ki-67 ≥55% (14 versus 10 months, p<0.001). The median survival for all patients receiving chemotherapy was 11 months (95% confidence interval 9.4-12.6 months). The median survival from start of first-line chemotherapy in patients who received second-line chemotherapy was 19 months (95% confidence interval 16.6-21.4 months).[16]

5.3 Study summary

As there is no standard treatment beyond first-line etoposide/platinum-based chemotherapy in patients with progressive poorly differentiated extra-pulmonary neuroendocrine carcinoma, this is an area of unmet need in this disease group.

Combination regimens such as irinotecan/5-FU/folinic acid are a second-line treatment option currently used in Europe and world-wide, without trial evidence, for this subset of patients and are more often

recommended for those patients with a NEC with a Ki-67≥55%, with temozolomide based-combinations being preferred for those with Ki-67<55%.[19]

Expanded analysis of the NAPOLI-1 phase III trial of nal-IRI, with or without 5-FU and folinic acid (leucovorin), versus 5-FU and folinic acid, in the treatment of patients with metastatic pancreatic adenocarcinoma after gemcitabine-based therapy reported a statistically significant increase in overall survival (6.1 months for the combination of nal-IRI with 5-FU/folinic acid compared to 4.2 months in the 5-FU/folinic acid control arm, p=0.012, in the intent to treat population, and 8.9 months and 5.1 months respectively in the per protocol population, p=0.011).[23]

Liposomal irinotecan has improved pharmacokinetics and tumour bio-distribution in comparison to irinotecan, and may have clinical benefit in patients with a NEC diagnosis.[23]

In devising treatment strategies for extra-pulmonary NEC, many refer to the extensive literature on high-grade NEC of the lung of which docetaxel is a second-line therapy option.[16]

Table 1 | Summary of studies of second-line treatment in patients with NECs.

Study	Treatment	Patients	PFS	OS
**Olsen** et al 2014 [18]	Topotecan monotherapy (heavily pre-treated)	N=22	2.1 months	3.2 months
**Hadoux** et al 2013 [16]	Oxaliplatin-based chemotherapy (mostly 5-FU, folinic acid & oxaliplatin [FOLFOX])	N=21	4.3 months (Longer with lower Ki67 levels)	9.5 months (Longer with lower Ki67 levels)
**Hentic** et al 2012 [15]	5-FU, folinic acid & irinotecan (FOLFIRI)	N=19	4 months	18 months (Includes 1st & 2nd line treatment)
**Olsen** et al 2012 [20]	Temozolomide monotherapy	N=28	2.4 months	3.5 months
**Welin** et al 2011 [17]	Temozolomide monotherapy or in combination with capecitabine & bevacizumab	N=25	6 months	22 months

The National Comprehensive Cancer Network (NCCN) Clinical Practice guidelines in Oncology for the treatment of small cell lung cancer include docetaxel[24] as a second-line treatment option in patients who have progressed on primary etoposide-platinum combination therapy.

Therefore, NET-02 has been designed as a multi-centre, randomised, open-label, phase II, single-stage selection trial of nal-IRI/5-fluorouracil/folinic acid or docetaxel as second-line therapy in patients with progressive poorly differentiated extra-pulmonary neuroendocrine carcinoma, with the aim of establishing the optimum combination for inclusion in a phase III study. Based on the observed response rates, survival, impact on quality of life, and toxicity profile, the optimal dose of docetaxel in pre-treated patients with non-small cell lung cancer is 75mg/m^2 intravenously every 3 weeks[25] and this dose will be utilised in this study.

5.4 Liposomal irinotecan (Nal-IRI)

5.4.1 Nal-IRI mechanism of action

Liposomal irinotecan is irinotecan encapsulated in a liposome drug delivery system. The active ingredient of the nal-IRI injection, irinotecan, is a member of the topoisomerase I inhibitor class of drugs and is a semi-synthetic and water-soluble analogue of the naturally-occurring alkaloid, camptothecin. Topoisomerase I inhibitors work to arrest uncontrolled cell growth by preventing the unwinding of deoxyribonucleic acid (DNA), and therefore preventing replication. The pharmacology of irinotecan is complex, with extensive metabolic conversions involved in the activation, inactivation, and elimination of the drug.[26-28] Irinotecan is a pro-drug that is converted by nonspecific carboxylesterases into a 100-1000 fold more active metabolite, SN-38.[28] The metabolite SN-38 is cleared via glucuronidation (for which major pharmaco-genetic differences have been shown), and biliary excretion. These drug properties contribute to the marked differences in efficacy and toxicity observed in clinical studies with irinotecan.[29-31]

Drug carrier technologies represent a rational strategy to improve the pharmacokinetics and bio-distribution of irinotecan, while protecting it from premature metabolism. Liposomal irinotecan employs a novel intra-liposomal drug stabilisation technology for encapsulation of irinotecan into long-circulating liposome-based particles with high drug load and high *in vivo* stability. The stable liposome formulation of irinotecan has several attributes that may provide an improved therapeutic index. The controlled and sustained release should improve activity of this schedule-dependent drug by increasing duration of exposure of tumour tissue to drug, an attribute that allows it to be present in a higher proportion of cells during the more sensitive S-phase of the cell cycle. The improved pharmacokinetics, high intravascular drug retention in the liposomes, and enhanced permeability and retention effect may potentially result in site-specific drug delivery to solid tumours. Stromal targeting results from the subsequent depot effect, where liposomes accumulating in tumour-associated macrophages release the active drug and convert it locally to the substantially more cytotoxic SN-38. The preferentially local bio-activation should result in reduced exposure to potential sites of toxicity and increased exposure to neighbouring cancer cells within the tumour.

5.4.2 Nal-IRI pre-clinical experience

Liposomal irinotecan, in pre-clinical settings, has been shown to have a broad spectrum of activity in a wide range of solid tumours including colon, pancreatic, gastric, cervical, non-small cell lung, small cell lung, ovarian, thyroid, and breast cancers, as well as glioma, Ewing's sarcoma, and neuroblastoma, often with a high degree of anti-tumour activity against resistant or difficult-to-treat cancer models.[32-34] Liposomal irinotecan has also shown potent anti-tumour activity, including durable tumour regression, and was markedly superior to the equivalent dose of free drug in a bioluminescent-based orthotopic xenograft pancreatic model.[35]

5.4.3 Nal-IRI pre-clinical pharmacokinetics

The pharmacokinetic (PK) properties of nal-IRI were evaluated in a HT-29 colon cancer subcutaneous xenograft model.[34] Both irinotecan and SN-38 were cleared very rapidly (within 8 hours) from the plasma following free irinotecan administration. However, nal-IRI clearance was demonstrated to be considerably slower and remained in circulation for over 50 hours. The SN-38 plasma exposure was also greater, though C_{max} levels were reduced following nal-IRI administration, suggesting the advantage of the irinotecan liposomal formulation in prolonging exposure and half-life via the ability of the lipid bilayer to protect the conversion of the prodrug nal-IRI to SN-38. Further, both irinotecan and SN-38 accumulated in tissues for extended time (at least 1 week after nal-IRI administration), yet there were relatively higher

levels of prolonged accumulation in the tumour compared to normal tissue, where the metabolites are at very low levels after 48 hours (**Figure 2**).[36]

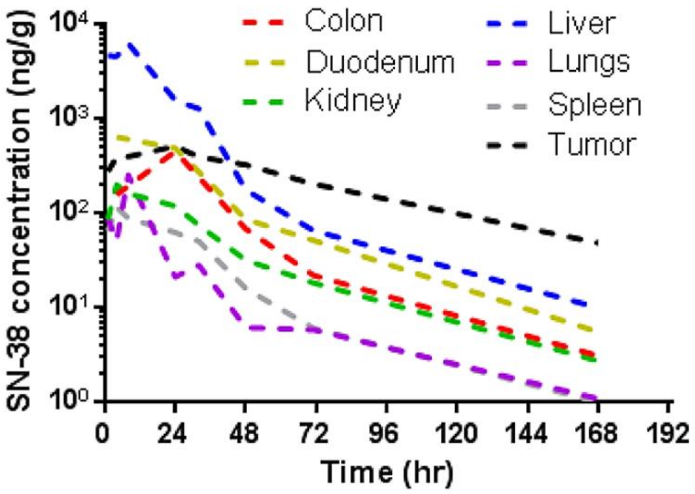

Figure 2 | Tissue distribution of nal-IRI in a HT-29 xenograft study in mice.

Levels of SN-38 in various tissues following a single nal-IRI (20 mg/kg) dose are shown. Prolonged accumulation of SN-38 (~168 h) seen in tumour compared to other organs (~48 h)

Activation of irinotecan to SN-38 by the liver is the primary path for SN-38 tumoural accumulation, when free irinotecan is administered. In contrast, these data suggest that accumulation of nal-IRI in the tumour and subsequent liposome breakdown and local conversion of irinotecan to SN-38 is responsible for the enhanced tumour exposure of SN-38, when nal-IRI is administered. These preclinical data demonstrating longer retention time in tumour lesions with nal-IRI administration compared to free irinotecan administration formed the basis for clinical development. For detailed information, see the current nal-IRI Summary of Product Characteristics (SmPC).

5.4.4 Clinical experience with nal-IRI

Liposomal irinotecan has been studied in patients with solid tumours, including cervical cancer, gastric cancer, pancreatic cancer, and colorectal cancer (**Table 2**). Disease areas currently being studied include glioma (intravenous and convection-enhanced local delivery), breast cancer and several paediatric solid tumours, including Ewing's sarcoma, rhabdomyosarcoma, neuroblastoma, and osteosarcoma. Nine clinical studies of nal-IRI have been completed with over 400 patients across multiple tumour types exposed to various dosing regimens, with additional studies actively recruiting patients across multiple tumour types.

Studies that have been completed with nal-IRI include:

- a multi-centre, open-label phase I dose-escalation study of MM-398 using a once-every-three-week dosing schedule in advanced solid tumour patients (PEP0201);
- a study of nal-IRI in combination with 5-FU/folinic acid in advanced solid tumour patients (PEP0203); a study of nal-IRI with or without cisplatin in cervical cancer (PEP0202), which was terminated early;
- a randomised phase II study of nal-IRI, irinotecan or docetaxel as a second-line therapy in patients with locally advanced or metastatic gastric or gastroesophageal junction adenocarcinoma (PEP0206);
- a randomised phase II study of nal-IRI in combination with 5-FU/folinic acid (FUPEP regimen) or FOLFIRI regimen in oxaliplatin pre-treated patients (PEPCOL);

- a phase II study of nal-IRI as a second-line therapy in patients with metastatic pancreatic cancer (PEP0208); and
- a randomised, open label phase III study of nal-IRI, with or without 5-FU and folinic acid, versus 5-FU and folinic acid, in patients with metastatic pancreatic cancer who have failed prior gemcitabine-based therapy (NAPOLI-1).[37]

Change in protocol v6.0 dated 3rd June 2020: the expression of strength of nal-IRI was changed in NET-02 protocol v6.0 due to a change in the product. The strength of nal-IRI was originally expressed as irinotecan hydrochloride trihydrate (5 mg/ml) but was changed to reflect irinotecan anhydrous free-base (4.3 mg/ml). As a consequence, the recommended starting dose was amended to 70 mg/m^2 of free-base (as opposed to 80 mg/m^2 salt-base). The nal-IRI doses quoted in this section remain as utilised in the original studies referenced and are therefore expressed as irinotecan hydrochloride trihydrate (5 mg/ml).

For detailed information see current nal-IRI SmPC.

Table 2 | Summary of some clinical studies with Nal-IRI

HZT; patients heterozygous for UGT1A, **WT**; patients wild type for UGT1A.

Study	Tumour type	Phase	Study design	Dosing frequency	Dose level (mg/m^2)	Combination	Combination dose	Key result
**PEP0201**	Solid tumours	1	Open label, dose escalation	Q3W	60 (n=1), 120 (n=6), 180 (n=4)	No	-	MTD 120 mg/m^2
**PEP0202**	Cervical	1	Open label, dose escalation	Q3W	60 (n=3), 80 (n=3)	Cisplatin	60mg	Terminated early; protocol violation
**PEP0203**	Solid tumours	1	Open label, dose escalation	Q3W	60 (n=3), 80 (n=6), 100 (n=5), 120 (n=3)	5-FU & LV	2000 & 500 mg/m^2	MTD 80 mg/m^2
**PEP0206**	Gastric	2	Open label, 3 arm (nal-IRI v docetaxel v irinotecan)	Q3W	120	No	-	Similar safety profile in all arms, 1° endpoint, 6 responses in nal-IRI arm
**PEP0208**	Pancreas	2	Open label, single arm	Q3W	120	No	-	Medial OS 5.2 months
**NAPOLI 1**	Pancreas	3	Randomised comparison of nal-IRI +/- 5-FU & LV	Q3W (mono)<br>Q2W (combo)	120 (mono), 80 (combo)	5-FU & LV	2000 & 200 mg/m^2	Combo arm median OS 6.1 months, 1.9 month improvement (HR=0.67, p=0.012)
**UCSF 8603**	Glioma	1	Open label, dose escalation	Q3W	HTZ: 60 (n=3), 90 (n=6), 120 (n=3), 150 (n=6)<br>WT: 120 (n=6), 180 (n=7), 240 (n=3)	No	-	Enrolment completed<br>MTD for HTZ 150mg/m^2<br>MTD for WT 120mg/m^2
**PIST CRC 01**	Colorectal	1	Open label, dose escalation	Q2W	80 (n=6), 90 (n=6), 100 (n=6)	No	-	Enrolment completed
**PEPCOL**	Colorectal	2	Nal-IRI & 5-FU & LV & Avastin v FOLFIRI & Avastin	Q2W	80	5-FU & LV & Avastin	2000 & 200 mg/m^2 & 5mg/kg	Enrolment completed
**MM 398 01 01 02**	Solid tumours	1	Open label, ferumoxytol MRI prior to first dose	Q2W	80	No	-	Enrolment ongoing
**UCSF 13 12025**	Glioma	1	Open label, dose escalation, convection-enhanced delivery for direct tumoural injection	Single dose	Dose / T volume:<br>20mg or 40mg / 1-4 cm^3<br>60mg / 2-5 cm^3<br>80mg / 2-5 cm^3	No	-	Enrolment ongoing
**SPOC 2012 001**	Paediatric solid tumours	1	Open label, dose escalation	Q3W	60 (n=3), 90 (n=3), 120 (n=3), 150, 180, 210 (to do)	Cyclo-phosphamide	250 mg/m^2	Enrolment ongoing

5.4.5 Nal-IRI pharmacokinetics in humans

The pharmacokinetics of nal-IRI was evaluated using sample-rich and sparse PK sampling across 6 studies (Study PEP0201, Study PEP0203, Study PEP0206, Study PIST-CRC-01, Study MM-398-01-01-02, and Study MM-398-07-03-01). Both non-compartmental analysis and population pharmacokinetic analysis were performed to evaluate the pharmacokinetic properties of nal-IRI. A summary of PK parameters from non-compartmental analysis is provided in **Table 3**. Please reference the SmPC for nal-IRI for additional information on PK.

Table 3 | Summary statistics of nal-IRI PK parameters across multiple PK studies (taken from the IB)
†t1/2 and AUC0-∞ were not calculated for a subset of patients due to insufficient number of samples in the terminal phase. NA = not available. Cmax are in µg/ml for total irinotecan and ng/ml for SN-38; AUC are in µg.h/ml for total irinotecan and ng.h/ml for SN-38.

PK Parameters	Dose, mg/m^2	Analytes					
		Total Irinotecan			SN-38		
		N	Median	%IQR	N	Median	%IQR
C_{max} [µg/ml or ng/ml]‡	80	25	38.0	36	25	4.7	89
	120	45	59.4	41	45	7.2	57
$t_{1/2}$ [h]	80	23†	26.8	110	13†	49.3	103
	120	45	15.6	198	40†	57.4	67
$AUC_{0-\infty}$ [h·µg/ml or h·ng/ml]‡	80	23†	1030	169	13†	587	69
	120	45	1258	192	40†	574	64
V_d [L/m^2]	80	23†	2.2	55	NA	NA	NA
	120	45	1.9	52	NA	NA	NA

5.4.6 Nal-IRI safety in humans

It has been shown in animal and human PK studies that once irinotecan is released from the nal-IRI liposomes, the conversion of irinotecan to SN-38 is similar to that of the un-encapsulated irinotecan. The safety of nal-IRI, therefore, may be indirectly compared with the safety of irinotecan, primarily based on a qualitative comparison of adverse reactions, as reported in the Camptosar United States and European Medicines Agency (EMA) label for irinotecan.[38 39] The comparison is qualitative, as both irinotecan and nal-IRI have been used in different doses and schedules as monotherapy and combination therapy with other chemotherapeutic agents; therefore, quantitative comparisons are difficult. The most common adverse reactions of irinotecan and nal-IRI are similar and are mainly gastrointestinal events and myelosuppression.

The common adverse reactions (>30%) observed in clinical studies with conventional irinotecan in combination with other agents are: nausea, vomiting, abdominal pain, diarrhoea, constipation, anorexia, mucositis, neutropenia, leukopenia (including lymphopenia), anaemia, thrombocytopenia, asthenia, pain, fever, infection, abnormal bilirubin, and alopecia. The common adverse reactions (>30%) observed in single agent irinotecan therapy in clinical studies are: nausea, vomiting, abdominal pain, diarrhoea, constipation, anorexia, neutropenia, leukopenia (including lymphopenia), anaemia, asthenia, fever, decrease in body weight, and alopecia.[38 39]

With respect to liposomal irinotecan, nal-IRI, when used in combination with 5-FU and folinic acid, the most common adverse reactions (≥20%) observed in clinical trials considered to be related are: diarrhoea, nausea, vomiting, decreased appetite, neutropenia, fatigue, anaemia, stomatitis and pyrexia. The overall safety profile of nal-IRI is presented in detail in the related SmPC. Additionally, **Table 4**

summarises ≥ Grade 3 safety data from the NAPOLI-1 trial comparing nal-IRI+5-FU/folinic acid (at a dose of 80 mg/m^2 given on a 2 week schedule), or nal-IRI monotherapy (at a dose of 120 mg/m^2 given on an every 3 week schedule), with 5-FU/folinic acid alone (given weekly for 4 weeks followed by 2 weeks of rest) in the same population of patients who had received prior gemcitabine therapy.

Table 4 | Summary of Grade 3 or higher adverse events in NAPOLI-1 study.[37]
[1] Per Common terminology criteria for adverse events (CTCAE) Version 5.0, [2] Includes only patients who had at least one post-baseline assessment.

		nal-IRI + 5-FU & LV (N=117)	nal-IRI (N=147)	5-FU & LV (N=134)
**% of patients with Grade ≥3 non-haematological AEs** (occurrence >5% is shown) [1]	Fatigue	14	6	4
	Diarrhoea	13	21	5
	Vomiting	11	14	3
	Nausea	8	5	3
	Asthenia	8	7	7
	Abdominal pain	7	8	6
	Decreased appetite	4	9	2
	Hypokalaemia	3	12	2
	Hypernatremia	3	6	2
**% of patients with Grade ≥3 haematological AEs** based on laboratory values [2]	Neutrophil count decreased	20	16	2
	Haemoglobin decreased	6	7	5
	Platelet count decreased	2	1	0

Please reference the nal-IRI SmPC and section 11.4 for additional information on undesirable effects, a summary of the safety profile and observed adverse reactions.

5.4.7 Interaction with other medicinal products

Information about drug interactions with nal-IRI is referenced from the published scientific literature for non-liposomal irinotecan. No dedicated drug interaction studies were conducted with nal-IRI.

Strong Cytochrome P4503A4 (CYP3A4) inducers

Patients receiving concomitant non-liposomal irinotecan and CYP3A4 enzyme-inducing anticonvulsants phenytoin, phenobarbital or carbamazepine have substantially reduced exposure to irinotecan (AUC reduction by 12% with St John's wort, 57%-79% with phenytoin, phenobarbital, or carbamazepine) and SN-38 (AUC reduction by 42% with St John's wort, 36%-92% with phenytoin phenobarbital, or carbamazepine).

Strong CYP3A4 inhibitors and Uridine diphosphate (UDP)-glucuronosyl transferase 1A1 (UGT1A1) inhibitors

Patients receiving concomitant non-liposomal irinotecan and ketoconazole, a CYP3A4 and UGT1A1 inhibitor, have increased SN-38 exposure by 109%. Therefore, co-administration of nal-IRI with other inhibitors of CYP3A4 (e.g. grapefruit juice, clarithromycin, indinavir, itraconazole, lopinavir, nefazodone,

nelfinavir, ritonavir, saquinavir, telaprevir, voriconazole) may increase systemic exposure of nal-IRI. Based on the drug interaction of non-liposomal irinotecan and ketoconazole, co-administration of nal-IRI with other inhibitors of UGT1A1 (e.g. atazanavir, gemfibrozil, indinavir) may also increase systemic exposure of nal-IRI.

Co-administration of nal-IRI with 5-fluorouracil/folinic acid does not alter the pharmacokinetics of nal-IRI based on the population pharmacokinetic analysis. No interaction of nal-IRI with other medicines is known.

5.5 Rationale for the Use of nal-IRI in combination with 5-FU and folinic acid

In addition to changing the chemosensitivity of tumour cells through modification of the tumour microenvironment, lowering hypoxia can indicate improved tumour vascularisation, and thereby delivery of small molecule therapies. Liposomal irinotecan treatment led to increased microvessel density 6 days after treatment, as measured by CD31 staining in a HT29 xenograft study. Furthermore, there is preliminary evidence that nal-IRI increases perfusion of Hoechst 33342 stain in primary pancreatic tumour models treated with a single dose of nal-IRI (**Figure 3**). In that experiment, Hoechst 33342 was administered to measure small molecule tumour penetration and not DNA staining. It was proposed, prior to NAPOLI-1, that if nal-IRI was as positive in humans, then a greater percentage of 5-FU/folinic acid would also be expected to be delivered to the tumour.[37]

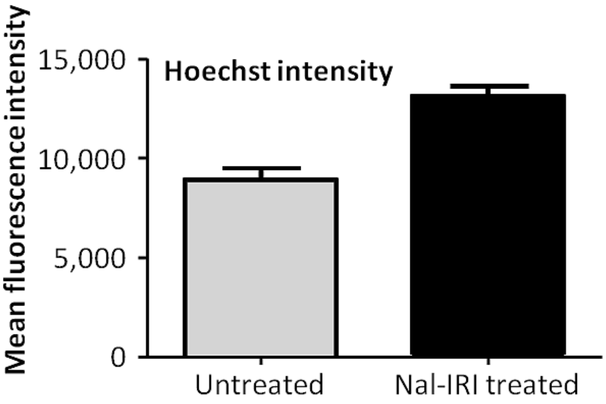

Figure 3 | Nal-IRI increases perfusion of small molecule Hoechst stain.

A primary pancreatic tumour was grown in non-obese diabetic - severe combined immunodeficiency (NOD-SCID) mice and given one dose of nal-IRI (20mg/kg). After 24 hours, Hoechst 33342 stain was administered 20 minutes prior to sacrificing the animal. Increase in stain intensity in treated mice was statistically significant, $p < 0.001$. Irinophore-C, another liposomal irinotecan formulation normalised tumour vascularisation in both subcutaneous and orthotopic mouse models.[40 41] In those studies, researchers demonstrated that multiple doses of liposomal irinotecan in the HT29 xenograft resulted in decreased hypoxia, as measured by CAIX (carbonic anhydrase IX) staining, and increased microvessel density, as measured by CD31 staining, similar to nal-IRI. More importantly, uptake of radiolabelled 5-FU increased by 50% following Irinophore-C treatment, consistent with the increase in Hoechst stain perfusion (**Figure 4**).[41]

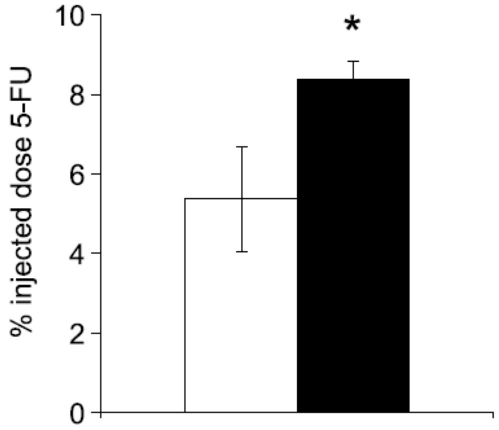

Figure 4 | Treatment with irinophore-C liposomal formulation of irinotecan increases uptake of radiolabelled 5-FU
Untreated = white bar, Irinophore-C treated = black bar.

Liposomal irinotecan is expected to have similar, if not more pronounced, tumour-modifying properties than Irinophore-C. A literature comparison of nal-IRI and Irinophore-C suggests nal-IRI is more efficacious in the HT29 model, and decreases CAIX to a greater extent.

In summary, pre-clinical evidence supports the hypothesis that nal-IRI modifies the tumour microenvironment in a manner that should make tumours more susceptible to 5-FU/folinic acid, through decreasing tumour hypoxia and increasing small molecule perfusion.

Preclinical studies have indicated that irinotecan has synergistic activity when it is administered before 5-fluorouracil and folinic acid.[42-45] Liposomal irinotecan has been shown to alter the hypoxic environment of pancreatic cancer in xenografts, promising concomitant improvement in activity of irinotecan and other chemotherapeutic agents such as 5-FU.

5.5.1 Efficacy of irinotecan in combination with 5-FU and folinic acid

The 5-FU combination with irinotecan has also been evaluated in patients who have failed 5-FU and other chemotherapeutic agents and for first-line therapy of colorectal cancer. There is also clinical evidence for the activity of the combination of irinotecan and 5-FU in pancreatic cancer. Chemotherapy-naive patients with histologically proven advanced pancreatic adenocarcinoma were treated with the FOLFIRI.3 regimen, consisting of irinotecan 90 mg/m^2 as a 60-minute infusion on day 1, folinic acid 400 mg/m^2 as a 2-hour infusion on day 1, followed by 5-fluorouracil 2000 mg/m^2 as a 46-hour infusion and irinotecan 90 mg/m^2, repeated on day 3, at the end of the 5-FU infusion, every 2 weeks.[46] Forty patients were enrolled, of whom 29 (73%) had metastatic disease. The confirmed response rate was 37.5%. Stable disease was observed in 27.5% of the patients. The median progression-free and overall survivals were 5.6 months and 12.1 months, respectively. The 1-year survival rate was 51%. Grade 3–4 neutropenia occurred in 35% of the patients, accompanied by fever in two cases. Other relevant grade 3–4 toxic effects were nausea, vomiting (27%) and diarrhoea (25%). Grade 2 alopecia occurred in 48% of the patients. There were no treatment-related deaths. These data indicated that the FOLFIRI.3 regimen seems to be active in advanced pancreatic cancer and to have a manageable toxicity profile.

The lack of cross-resistance between FOLFIRI.3 and gemcitabine-based regimens allows for effective second-line therapies. A prospective phase II study in 31 subjects showed a disease control rate of 23% and an overall survival of 3.8 months.[47] Two retrospective studies of 40 and 70 subjects respectively, showed disease control rates of 50% and 44.3%, and overall survivals of 6 and 5.5 months, with similar toxicity profiles.[48 49]

5.5.2 Efficacy of nal-IRI in combination with 5-FU and folinic acid

Given the relative absence of overlapping toxic effects among 5-fluorouracil, folinic acid, and nal-IRI, a regimen combining these agents was studied in a phase I trial of solid tumours in 16 subjects, of whom 5 were patients with pancreatic cancer. The objective tumour response rate, duration of response, and disease control rate were efficacy endpoints of the study. Among the 15 efficacy-evaluable patients, 2 (13.3%) had confirmed PR, 9 (60.0%) had SD, and 4 (26.7%) had PD. The overall disease control rate was 73.3%. Partial response was observed in one patient with gastric cancer (at 80 mg/m^2 dose level) and one patient with breast cancer (at 100 mg/m^2 dose level), with the duration of response of 142 and 76 days, respectively. Among the 6 patients who received the maximum tolerated dose (MTD) of 80 mg/m^2, there was 1 partial response (PR), 4 patients with stable disease (SD) and 1 with progressive disease (PD). The tumour response rate and disease control rate were 16.7% and 83.3%, respectively. The main DLTs (dose limiting toxicities) were grade 3 diarrhoea, leucopenia, neutropenia and febrile neutropenia. The MTD for nal-IRI was 80 mg/m^2.

The NAPOLI-1 trial compared nal-IRI+5-FU/folinic acid (at a dose of 80 mg/m^2 given on a 2 week schedule) and nal-IRI monotherapy (at a dose of 120 mg/m^2 given on an every 3 week schedule), with 5-FU/folinic acid alone (given weekly for 4 weeks followed by 2 weeks of rest) in 417 patients. This trial showed that nal-IRI+5-FU/folinic acid significantly improved the overall survival of patients with metastatic pancreatic ductal adenocarcinoma previously treated with gemcitabine therapy.[37]

5.5.3 Safety data for the combination of Nal-IRI, 5-FU and folinic acid

In the NAPOLI-1 randomised phase 3 trial, liposomal irinotecan in combination with 5-fluorouracil and folinic acid extended survival with a manageable safety profile in patients with metastatic pancreatic ductal adenocarcinoma who previously received gemcitabine-based therapy. The grade 3 or 4 adverse events that occurred most frequently in the 117 patients assigned liposomal irinotecan plus 5-fluorouracil and folinic acid were neutropenia (32 [27%]), diarrhoea (15 [13%]), vomiting (13 [11%]), and fatigue (16 [14%]).[37]

In the phase I dose-escalation study of nal-IRI in combination with 5-FU/folinic acid in advanced solid tumours (PEP0203), a total of 401 episodes of adverse events (AEs) were reported from the 16 treated subjects (safety population), of which 74 (18.4%) were of common terminology criteria (CTC) grade 3 or above. Among all AEs, 231 (57.6%) were considered by the investigators to be treatment-related. The most common treatment-related AEs included nausea (81.3%), diarrhoea (75.0%), vomiting (68.8%), fatigue (43.8%), mucositis (43.8%), leucopenia (37.5%), neutropenia (37.5%), weight loss (37.5%), anaemia (31.3%), and alopecia (31.3%). Acute cholinergic diarrhoea was rarely observed. **Table 5** (see below) describes the incidence of treatment-emergent adverse events by maximum CTC grade and by causality (incidence ≥20%), as seen in the PEP0203 study.

Table 5 | Incidence of treatment-emergent adverse events by maximum CTC grade and by causality (incidence ≥20%) in the PEP0203 study.

[1]Severity grading used the highest grading ever rated for each subject if the subject had such adverse event reported

[2] Defined as subject ever experienced AE related to the study drug in causality or not.

			Severity grade[1]				Causality[2]	
**System organ class**	**Preferred term**	**N = 16**	**I**	**II**	**III**	**IV**	**Yes**	**No**
**Blood and lymphatic system disorders**	Anaemia	7 (43.8%)	3	2	2	0	5	2
	Leucopenia	6 (37.5%)	0	3	2	1	6	0
	Neutropenia	6 (37.5%)	0	2	3	1	6	0
**Gastrointestinal disorders**	Abdominal pain	7 (43.8%)	3	2	2	0	3	4
	Constipation	6 (37.5%)	3	3	0	0	0	6
	Diarrhea	12 (75.0%)	3	4	5	0	12	0
	Nausea	13 (81.3%)	6	6	1	0	13	0
	Vomiting	12 (75.0%)	3	8	1	0	11	1
**General disorders and administration site conditions**	Fatigue	8 (50.0%)	4	3	1	0	7	1
	Mucosal inflammation	7 (43.8%)	4	3	0	0	7	0
	Pyrexia	7 (43.8%)	3	4	0	0	2	5
**Infections and infestations**	Infection	6 (37.5%)	0	3	3	0	2	4
**Investigations**	ALT increased	5 (31.3%)	3	2	0	0	4	1
	AST increased	4 (25.0%)	3	1	0	0	1	3
	Weight decreased	8 (50.0%)	4	4	0	0	6	2
**Metabolism and nutrition disorders**	Anorexia	4 (25.0%)	1	2	1	0	3	1
	Hypoalbuminemia	4 (25.0%)	0	3	1	0	0	4
	Hypocalcaemia	5 (31.3%)	1	4	0	0	0	5
	Hypokalemia	8 (50.0%)	2	0	5	1	2	6
	Hyponatremia	4 (25.0%)	2	0	0	2	0	4
**Nervous system disorders**	Dizziness	4 (25.0%)	4	0	0	0	1	3
**Psychiatric disorders**	Insomnia	4 (25.0%)	4	0	0	0	1	3
**Respiratory, thoracic and mediastinal disorders**	Cough	5 (31.3%)	3	1	1	0	0	5
**Skin and subcutaneous tissue disorders**	Alopecia	5 (31.3%)	5	0	0	0	5	0

5.6 Rationale for the use of docetaxel

The NCCN Clinical Practice guidelines in Oncology for the treatment of small cell lung cancer include docetaxel as a second-line treatment option in patients who have progressed on primary etoposide-platinum combination therapy.[25]

Based on the observed response rates, survival, impact on quality of life, and toxicity profile, the optimal dose of docetaxel in pre-treated patients with non-small cell lung cancer is 75mg/m^2 every 3 weeks,[26] and this dose will hence be utilised in the NET-02 trial.

5.7 Study setting

There is no standard treatment in the second-line setting for patients with poorly differentiated extra-pulmonary neuroendocrine carcinoma who have progressed following etoposide/platinum-based chemotherapy. The overall trial objective is to select a treatment for continuation to a Phase III trial. The NET-02 trial design directly addresses this overall objective and is an adaptation of a one-stage treatment design proposed by Simon, Wittes and Ellenberg[50], where the A'Hern design is first implemented to assess efficacy of each treatment separately to ensure a pre-specified minimum level of activity prior to selection. Should both treatments be deemed sufficiently efficacious, selection criteria will then be applied following the design of Simon, Wittes and Ellenberg, to establish which treatment to take forward into a Phase III trial.

6. Aims and Objectives

The NET-02 study is a randomised, parallel group, open-label, phase II, single-stage selection trial of nal-IRI and 5-FU/folinic acid or docetaxel as second-line therapy in patients with progressive poorly differentiated extra-pulmonary NEC.

6.1 Aim

The aim of this study is to assess the efficacy of nal-IRI/5-FU/folinic acid or docetaxel, separately, as second-line therapy in patients with progressive poorly differentiated extra-pulmonary NEC, with selection criteria applied to establish which treatment to take forward into a phase III trial.

6.2 Trial objectives

6.2.1 Primary objective

The primary objective of this study is to determine the 6 month progression-free survival rate (defined as a binary outcome (progression-free or not) within the timeframe of treatment start date until 6 months after randomisation) in patients with progressive poorly differentiated extra-pulmonary neuroendocrine carcinoma receiving second-line treatment with nal-IRI/5-fluorouracil/folinic acid or docetaxel.

6.2.2 Secondary objectives

The secondary objectives of this study are to determine the progression-free survival (defined as the time from randomisation to progression or death from any cause), overall survival, objective response rate using RECIST 1.1[1] measurements, toxicity, quality of life and whether neuron-specific enolase levels are predictive of treatment response in patients with progressive poorly differentiated extra-pulmonary neuroendocrine carcinoma receiving second-line treatment with nal-IRI/5-fluorouracil/folinic acid or docetaxel.

6.2.3 Samples for research

The exploratory objectives of this study include quantification of circulating tumour cells and circulating tumour DNA at baseline, 6 weeks and on progression, to identify any correlation with disease-related outcomes. The study will also carry out molecular profiling of circulating tumour cells, circulating tumour DNA and tumour tissue (further immunohistochemistry on tumour tissue may also be required) to identify any correlation with disease-related outcomes. The study may also generate mouse models of neuroendocrine carcinoma for further investigation into the disease.

7. Design

This is a multi-centre, randomised, parallel group, open-label, phase II, single-stage selection trial of nal-IRI/5-fluorouracil/folinic acid or docetaxel as second-line therapy in patients with progressive poorly differentiated extra-pulmonary neuroendocrine carcinoma, with the aim of selecting a treatment for continuation to a phase III trial.

The design is an adaptation of a one-stage treatment design proposed by Simon, Wittes and Ellenberg[50] where the A'Hern design is first implemented to assess efficacy of each treatment separately, to ensure a pre-specified minimum level of activity prior to selection. Selection criteria are then applied following the design of Simon, Wittes and Ellenberg, should both treatments be deemed sufficiently efficacious, to establish which treatment to take forward into a phase III trial. The intention of the trial is to show that the regimens are sufficiently active in this population of patients, but not to show that one regimen is significantly superior to the other.

The A'Hern method is advantageous over other single-stage designs, since it uses the exact binomial distribution as opposed to a normal approximation to the binomial distribution which can give a substantial margin of error in small trial sizes. Additionally, cut-off points are created which, if reached, could enable earlier planning for a phase III follow-on trial.

One hundred and two eligible participants will be randomised (1:1) to receive either nal-IRI, 5-FU and racemic folinic acid given every 14 days, or docetaxel given every 21 days.

Participants will be treated for a minimum of 6 months. Trial treatment will continue until progressive disease, intolerable toxicity, delay of treatment for more than 28 days, development of any condition or occurrence of any event, which, in the opinion of the local investigator, justifies discontinuation of treatment, patient request or until 6 months after the last participant is randomised, whichever comes first.

8. Eligibility

Eligibility waivers to the inclusion and exclusion criteria are **not** permitted. Queries in relation to the eligibility criteria must be addressed prior to randomisation. Patients are eligible for the trial if all the inclusion criteria are met and none of the exclusion criteria applies.

8.1 Inclusion criteria

1. Age ≥18 years and life expectancy >3 months.
2. Diagnosed with poorly differentiated (as defined by the World Health Organisation in 2019, Ki 67 >20%) extra-pulmonary neuroendocrine carcinoma (NEC grade 3, confirmed by histology). (Carcinoma of unknown primary is allowed if lung primary has been excluded following review by the multi-disciplinary team).
3. Prior treatment with first-line platinum-based chemotherapy for NEC in the advanced setting and ≥28 days from Day 1 of the previous treatment cycle.
4. Documented radiological evidence of disease progression OR discontinuation of first-line platinum-based chemotherapy due to intolerance.
5. Measurable disease according to RECIST 1.1 (Appendix 1).
6. Eastern Co-operative Oncology Group (ECOG) performance status ≤2 (see Appendix 2).
7. Adequate renal function with serum creatinine ≤1.5 times upper limit of normal (ULN) and creatinine clearance ≥30ml/min according to Cockroft-Gault or Wright formula (see Appendix 3). If the calculated creatinine clearance is less than 30 ml/min, glomerular filtration rate (GFR) may be assessed using either Cr51-EDTA or 99mTc-DTPA clearance method to confirm if GFR is ≥ 30 ml/min).
8. Adequate haematological function: Hb ≥90g/L, WBC ≥3.0 x 10^9/L, ANC ≥1.5 x 10^9/L, platelet count ≥100 x 10^9/L.
9. Adequate liver function: serum total bilirubin ≤1.5 x ULN (biliary drainage is allowed for biliary obstruction) and ALT and/or AST ≤2.5 x ULN in the absence of liver metastases, or ≤5 x ULN in the presence of liver metastases.
10. A negative pregnancy test is required at registration in women of childbearing potential[*].
11. Men[†] and women[*] of reproductive potential must agree to use a highly effective form of contraception[‡] during the study and for 6 months following the last dose of trial treatment. In addition, male participants should use a condom during study participation and for 6 months following the last dose of trial treatment.
12. Patients must be able to provide written informed consent.
13. Patients must be able and willing to comply with the terms of the protocol.

---

[*] Women of reproductive potential are defined as fertile, following menarche and until becoming post-menopausal unless permanently sterile. Permanent sterilisation methods include hysterectomy, bilateral salpingectomy and bilateral oophorectomy.

[†] Men of reproductive potential are defined as post-pubescent and not permanently sterile by vasectomy or bilateral orchidectomy.

[‡] Highly effective contraception is defined as one of the following: combined (oestrogen and progesterone-containing) hormonal contraception associated with inhibition of ovulation (oral, intravaginal or transdermal); progesterone-only hormonal contraception associated with inhibition of ovulation (oral, injectable, implantable); intrauterine device (IUD); intrauterine hormone-releasing system (IUS); bilateral tubal occlusion; vasectomised partner; practising true abstinence (when this is in line with the preferred and usual lifestyle of the subject).

8.2 Exclusion criteria

1. Known or suspected allergy or hypersensitivity reaction to any of the components of study treatment or their excipients.
2. Use (including self-medication) within one week of randomisation and for the duration of the study of any of the following: St. John's wort, grapefruit, Seville oranges, medicines known to inhibit UGT1A1 (e.g. atazanavir, gemfibrozil, indinavir) and medicines known to inhibit or induce either CYP3A4 or CYP3A5 (see Appendix 8 for list*).
3. Previous treatment (for neuroendocrine carcinoma) with any of the components of combination chemotherapy regimens detailed in this study (nal-IRI or 5-FU or irinotecan or topoisomerase inhibitors or taxane-based therapy).
4. Incomplete recovery from previous therapy in the opinion of the investigator (surgery/adjuvant therapy/radiotherapy/chemotherapy in advanced setting), including ongoing peripheral neuropathy of > Common Terminology Criteria for Adverse Events (CTCAE) grade 2 from previous platinum-based therapy.
5. Concurrent palliative radiotherapy involving target lesions used for this study (<28 days from discontinuation of radiotherapy). Radiotherapy for non-target lesions is allowed if other target lesions are available outside the involved field.
6. Patients must not have a history of other malignant diseases (within the previous 3 years, and there must be no evidence of recurrence), other than:
    - Extra-pulmonary neuroendocrine carcinoma.
    - Non-melanoma skin cancer where treatment consisted of resection only or radiotherapy.
    - Ductal carcinoma *in situ* (DCIS) where treatment consisted of resection only.
    - Cervical carcinoma *in situ* where treatment consisted of resection only.
    - Superficial bladder carcinoma where treatment consisted of resection only.
7. Documented brain metastases, unless adequately treated (surgery or radiotherapy only), with no evidence of progression and neurologically stable off anticonvulsants and steroids.
8. Clinically significant gastrointestinal disorder (in the opinion of the treating clinician) including hepatic disorders, bleeding, inflammation, obstruction, or diarrhoea > CTCAE grade 1 (at time of study entry).
9. Severe arterial thromboembolic events (myocardial infarction, unstable angina pectoris, stroke) less than 6 months before inclusion.
10. New York Heart Association (NYHA) Class III or IV congestive heart failure, ventricular arrhythmias or uncontrolled blood pressure†.
11. Severe bone marrow failure or bone marrow depression after radiotherapy or treatment with other antineoplastic agents (defined as haematological values of haemoglobin or white blood cells or neutrophils or platelets not meeting inclusion criteria).
12. Known active hepatitis B virus, hepatitis C virus or HIV infection. Infection status should be confirmed in cases of clinical suspicion.
13. Active chronic inflammatory bowel disease.
14. Breastfeeding women.
15. Evidence of severe or uncontrolled systemic diseases which, in the view of the treating clinician, makes it undesirable for the patient to participate in the trial.[3]
16. Evidence of significant clinical disorder or laboratory finding which, in the opinion of the treating

* For patients receiving any of these medications, use of an alternative agent is recommended.

† It is recommended that subjects should have a systolic blood pressure of either less than 150 mmHG, and/or a diastolic blood pressure of less than 100 mmHg at rest (average of 3 consecutive readings 3-5 minutes apart). Anti-hypertensive drugs may be used to achieve these values.

[3] Including active COVID-19.

clinician, makes it undesirable for the patient to participate in the trial.
17. Medical or psychiatric conditions that impair the ability to give informed consent.
18. Any other serious uncontrolled medical conditions (in the opinion of the treating clinician).
19. Use of warfarin or warfarin-type anti-coagulation therapies within one week of randomisation and for the duration of the study (the use of low molecular weight heparin is permitted as appropriate).

9. Recruitment Process

9.1 Recruitment Setting

Patients will be recruited from multiple trial sites throughout the UK. Research sites will be required to have obtained management approval and undertake a site initiation meeting with the CTRU prior to the start of recruitment into the trial.

The trial aims to recruit 102 participants over a 37-month period.

9.2 Eligibility Screening

All participating trial sites will be required to complete regular Screening Logs of all patients who do not go on to be registered. For screened patients who do not go on to be registered into the trial, anonymised data will be recorded for whether or not the patient was eligible for participation. For patients who were not eligible, the reason for ineligibility is recorded; for patients who were eligible, the reason for the patient not entering the study is recorded. However, the right of the patient to refuse consent without giving reasons is respected. Screening forms should be returned to the CTRU on a regular basis.

9.3 Informed Consent and Eligibility

The Principal Investigator (PI) retains overall responsibility for the informed consent of participants at their site and must ensure that any person delegated responsibility to participate in the informed consent process is duly authorised, trained and competent to participate according to the ethically approved protocol, principles of Good Clinical Practice (GCP) and Declaration of Helsinki 1996.

Assenting participants will be broadly assessed for eligibility during the screening process based on their medical history according to the inclusion and exclusion criteria (review of diagnosis, treatment history and key eligibility criteria).

The right of a patient to refuse participation without giving reasons will be respected. The participant must remain free to withdraw at any time from the study without giving reasons and without prejudicing his/her further treatment and will be provided with a contact point where he/she may obtain further information about the trial. Where a participant is required to re-consent or new information is required to be provided to a participant, it is the responsibility of the PI to ensure this is done in a timely manner and according to any timelines requested by the CTRU.

9.3.1 Initial Information and Initial Approach

Potentially-eligible participants will be identified at each site. The participants must have received first-line platinum-based chemotherapy and be attending follow-up clinics in Oncology Departments at each site. When progressive disease following first-line platinum-based chemotherapy is evidenced, the patients may be considered for this clinical trial.

Potential participants will be approached regarding trial participation during the standard clinic visit at which their progression following first-line chemotherapy is discussed. Potential participants will be provided with a verbal and written explanation of the trial. The PI, or, where delegated by the PI, other appropriate site staff who are trained in Good Clinical Practice (GCP), and are authorised on the trial delegation log, will provide a full explanation of the trial and all relevant treatment options to each patient. The current approved Patient Information Sheet (PIS) for the trial will be provided to the patient and will be discussed. A contact number will be given to the patient, should they wish to discuss any aspect of the trial with site staff.

9.3.2 Consent Process

After the patient has been given adequate time to consider the information provided (a minimum of 24 hours), all queries have been addressed and the clinical team is confident that the patient understands the trial and all requirements, patients will be consented onto the trial by signing a current approved consent form.

A record of the consent process detailing the date of consent and all those present will be kept in the participants' medical notes. The original consent form(s) will be filed in the Investigator Site File, a copy of the consent form(s) will be given to the participant, a copy will be filed in the hospital notes (as per local practice) and a copy of the consent form(s) will be returned to the Clinical Trials Research Unit (CTRU), at the University of Leeds.

An original OR copy of the patient consent form should be given to the patient (each site's local policy/preference may be followed regarding whether this is a second original copy of the consent form or a photocopy).

Where the patient is able to provide fully informed consent but is unable to sign or otherwise mark the consent form, provision for completion of the consent form by a witness will be made. This should be a carer, friend/family member, or a local member of the clinical team who is independent of the research team.

After written informed consent, participants will be registered on the trial and screening assessments which are not part of standard of care can be performed. When all eligibility criteria are confirmed, the participant can be randomised.

All participants are free to withdraw at any time from the protocol treatment without giving reasons and without prejudicing further treatment. Where a participant is required to re-consent or new information is required to be provided to a participant, it is the responsibility of the PI to ensure this is done in a timely manner and according to any timelines requested by the CTRU. Withdrawal of consent from trial participation must be explicitly documented in the source documents.

General Practitioners (GPs) will be informed of their patient's trial participation with the participant's consent.

Where valid informed consent is obtained from the participant and the participant subsequently becomes unable to provide ongoing informed consent by virtue of physical or mental incapacity, the consent previously given when capable remains legally valid. Participants who lose capacity after informed consent has been obtained will continue with protocol treatment, assessments and follow-up in consultation with the PI and participant's carer/family with the participant's best interests foremost in the decision-making process. Ongoing collection of safety and follow-up data will continue via the clinical care team for inclusion in the trial analysis in order to preserve the integrity of the trial's intention to treat analysis and fulfil regulatory requirements specifically for pharmacovigilance purposes. The PI will take responsibility for ensuring that all vulnerable subjects are protected and participate voluntarily in an environment free from coercion or undue influence.

9.4 Registration and randomisation procedures

Recruitment of participants to the NET-02 trial requires trial-specific investigations to confirm eligibility. Therefore, recruitment is a two-step process involving initial registration of all potential participants prior to conducting trial-specific investigations to confirm eligibility, followed by randomisation for those patients found to be eligible.

Participants should be registered into the trial before any trial-specific procedures are performed.

All patients who consent to trial-specific eligibility assessment should be registered into the trial.

Participants who consent but are ineligible may be re-consented and re-screened if the reason for their ineligibility was temporary. Participants who are re-screened will not be re-registered and will keep their original trial number.

9.6.1 Registration

Informed written consent for entry into the trial must be obtained prior to registration. As soon as possible following written informed consent, participants will be registered into the trial by an authorised member of staff at the trial research site. All participants who consent to the trial should be registered.

Registration will be performed centrally using either the CTRU automated 24-hour telephone registration system or through the CTRU web-based system. Authorisation Codes and Personal Identification Numbers (PINs), provided by the CTRU, will be required to access both registration systems. Please note codes and PINs should be kept confidential.

The following information will be required at registration:

- Site code
- Name of person making the registration
- Confirmation of written informed consent
- Participant details, including participant initials, gender and date of birth.

Register the participant using either:
• 24-hour telephone line 0113 343 2290
or
• Web registration
https://lictr.leeds.ac.uk/webrand/
Please complete Form 01 Registration prior to registering the participant

9.6.2 Post-registration actions

Following registration, participants will be allocated a unique trial number. The trial number will be used for the purpose of participant identification and data collection during the study.
An e-mail confirmation of registration will be sent to the caller and Principal Investigator by the CTRU. These notifications are generated and sent automatically from the CTRU and should be reviewed by the recipients and filed in the NET-02 Investigator Site File.

The following eligibility assessments must be carried out as detailed below and no more than 14 days prior to randomisation:

- Full blood count (Hb, WBC, ANC, Platelet count).
- Biochemistry; sodium, potassium, magnesium, urea, creatinine, albumin, adjusted calcium, serum bilirubin, alkaline phosphatase (ALP), AST and/or AST, lactate dehydrogenase (LDH).
- Estimation of renal function (using the Cockroft-Gault formula (as defined in Appendix 3, Wright formula may be used in place of Cockroft-Gault if this is usual local practice)). If the calculated creatinine clearance is less than 30 ml/min, glomerular filtration rate (GFR) may be assessed using either Cr51-EDTA or 99mTc-DTPA clearance method to confirm if GFR is ≥ 30 ml/min).
- An ECG is required for **all** trial participants. ECGs done as part of standard care may be used providing they were carried out during this timeframe (no more than 14 days prior to randomisation).
- A pregnancy test for women of childbearing potential.

Following confirmation of eligibility, the participant will be randomised into the trial.

If a participant does not meet the eligibility criteria, a Non-Randomisation Form should be completed and sent to the CTRU.

9.6.3 Randomisation

Randomisation will be performed centrally using either the CTRU automated 24-hour telephone randomisation system or through the CTRU web-based system. Authorisation codes and PINs, provided by the CTRU, will be required to access both randomisation systems. Please note codes and PINs should be kept confidential.

The following information will be required at randomisation:

- Name of caller.
- Site code.
- Participant details including trial number and date of birth.
- Confirmation of eligibility.
- Confirmation of completion of the QoL booklet.
- Ki-67 value.
- ECOG performance status.
- Presence of liver metastases.
- Platinum-resistant disease (progression ≤ 6 months of completion[*] of platinum-based therapy), sensitive disease (progression >6 months from completion of platinum-based therapy) or platinum intolerant.

Randomise the participant using either:
• 24-hour telephone line 0113 343 2290
or
• Web randomisation
https://lictr.leeds.ac.uk/webrand/
Please complete F02 Eligibility Checklist and F04 Randomisation before randomising the participant

9.6.4 Treatment Allocation

Patients will be randomised using minimisation with a random element on a 1:1 basis to receive either docetaxel or nal-IRI (in combination with 5-FU/folinic acid). Treatment groups will be well-balanced for the following clinical characteristics:

- Hospital site
- Ki-67 marker
  - <55%
  - ≥55%
- ECOG performance status
  - 0/1
  - 2
- Presence of liver metastases
  - Yes
  - No
- Response to first-line platinum-based chemotherapy
  - Resistant disease (progression ≤ 6 months of completion[†] of platinum-based therapy)
  - Sensitive disease (progression >6 months from completion[*] of platinum-based therapy)
  - Platinum intolerant

[*] Date of 'completion of platinum-base therapy' is defined as day 1 of the last cycle given.
[†] Date of 'completion of platinum-based therapy' is defined as day 1 of the last cycle given.

9.6.5 Post-randomisation actions

An e-mail confirmation of the participant randomised into the trial will be sent to the caller, Principal Investigator and pharmacist by the CTRU. These notifications are generated and sent automatically from the CTRU and once reviewed by the recipients, filed in the NET-02 Investigator Site File. The participant's details should be added to the Participant ID Log.

Upon randomisation, participants will be given a trial-specific participant card, which will have the trial title, participant ID number, contact details of the Principal Investigator and out of hours contact details in cases of emergency.

10. Trial Medicinal Product Management

Please refer to the NET-02 Pharmacy and Investigational Medicinal Product (IMP) Study Site Operating Procedure (SSOP) for full details of the trial IMP management requirements.

Within the trial the following are classed as IMPs:

- **Liposomal irinotecan anhydrous free base (nal-IRI)**
    - Brand name ONIVYDE, manufactured by Servier.
    - nal-IRI is not equivalent to non-liposomal irinotecan formulations and must not be interchanged.

- **5-Fluorouracil**
    - Generic product is acceptable for use, off-the-shelf supply.

- **Docetaxel**
    - Generic product is acceptable for use, off-the-shelf supply.

Within the trial the following are classed as a Non Investigational Medicinal Products (NIMPs):

- **Folinic acid**
    - Generic product is acceptable for use, off-the-shelf supply.

- **Granulocyte-Colony Stimulating Factor (G-CSF)**
    - Generic product is acceptable for use, off-the-shelf supply.

10.1 Liposomal Irinotecan Anhydrous Free-base (nal-IRI)

10.1.1 Composition of nal-IRI

Liposomal Irinotecan is a liposome drug delivery system encapsulating irinotecan. It is supplied as sterile, single-use vials containing 10 ml of nal-IRI at a concentration of 4.3 mg/ml. One 10 ml vial of concentrate contains the equivalent of 43 mg irinotecan anhydrous free-base (as irinotecan sucrosofate salt in a pegylated liposomal formulation).

Change in protocol v6.0 dated 3rd June 2020: the expression of strength of nal-IRI was changed due to a change in the product. The strength of nal-IRI was originally expressed as irinotecan hydrochloride trihydrate (5 mg/ml) but was changed to reflect irinotecan anhydrous free-base (4.3 mg/ml). As a consequence, the recommended starting dose was amended to 70 mg/m^2 of free-base (as opposed to 80 mg/m^2 salt-base). The amount of active ingredient in the vial was not changed.

10.1.2 Supply, distribution and handling of nal-IRI

Liposomal Irinotecan will be provided to sites free of charge for use in this clinical trial. It will be supplied by Servier, labelled for trial use, stored and distributed to sites by Clinigen. Clinigen (IDIS, now part of Clinigen, is the Manufacturing Authorisation holder) hold a Manufacturer's Authorisation for IMPs. Liposomal Irinotecan will be labelled with a trial-specific label in accordance with Directive 2001/20/EC and the Medicines for Human Use (Clinical Trials) Regulations 2004 (as amended).

Once received at site, all trial IMP stock must be documented as received in accordance with the NET-02 Pharmacy and IMP SSOP provided within the NET-02 Pharmacy Site File.

Liposomal Irinotecan must be refrigerated at 2 to 8°C and the vials are to be stored in the outer carton provided, to ensure that they are not stored in direct light. Light protection is not required during infusion. Liposomal Irinotecan **must not be frozen**. In the event that a temperature excursion occurs, the pharmacy should contact CTRU as soon as possible.

Responsible individuals should inspect vial contents for particulate matter before and after the drug product is drawn from a vial into a syringe. In the event of a problem with the IMP, CTRU must be contacted immediately.

Liposomal Irinotecan must be diluted prior to administration. The diluted solution is physically and chemically stable for 6 hours at room temperature (as per SmPC), but it is preferable that it is stored at refrigerated temperatures (2-8°C), and protected from light. The diluted solution must not be frozen.

Liposomal Irinotecan is a cytotoxic medicinal product. The use of glove, goggles and protective clothing when handling or administering nal-IRI is recommended. Pregnant staff should not handle nal-IRI.

The trial supply of nal-IRI must not be used for any purpose other than that outlined in this protocol and should be stored in a secure ring-fenced location within the site pharmacy. Receipt of nal-IRI at participating centres and trial stock dispensed to participants must be recorded on the NET-02 Accountability and Dispensing Logs. These completed logs will be returned to CTRU upon request, to facilitate central IMP reconciliation.

Unused vials of nal-IRI should be counted, recorded and the record returned to CTRU prior to local destruction. Destruction should take place according to standard operating practice and local regulatory and environmental requirements. A record of any such destruction must be filed in the Pharmacy Site File and a copy sent to the CTRU. Unused nal-IRI should only be destroyed following authorisation from CTRU.

10.2 5-Flurouracil (5-FU) and folinic acid

10.2.1 Composition of 5-FU and folinic acid

5-Fluorouracil is typically available as a fluorouracil 50mg/ml solution for injection.

Folinic acid is typically available as a 10mg/ml folinic acid solution, provided as calcium folinate.

10.2.2 Supply, distribution, and handling of 5-FU and folinic acid

5-Fluorouracil and folinic acid are commercially available and should be sourced locally as per standard practice at the investigator sites using products that have a UK or EU license. As individual sites may use different brands or manufacturers for these drugs, each site is responsible for placing the most recent SmPC for the brand being used at site in the local pharmacy folder, or a file note that makes reference to the electronic source. Trial-specific labels will not be used for these IMPs.

Descriptive information for 5-FU and folinic acid can be found in the package insert. Study treatment with 5-FU and folinic acid should be administered according to the institutional standards at each site. 5-fluorouracil and folinic acid will be used and stored as detailed on the product label and according to manufacturer's instructions. There will be no re-imbursement to sites for 5-FU and folinic acid used during trial treatment.

Accountability of 5-FU and folinic acid is as per local standard practice.

10.3 Docetaxel

10.3.1 Composition of docetaxel

Docetaxel is typically available as a 10mg/ml concentrate solution for infusion.

10.3.2 Supply, storage and handling of docetaxel

Docetaxel is commercially available and should be sourced locally as per standard practice at the investigator sites, using products that have a UK or EU license. As individual sites may use different brands or manufacturers for this drug, each site is responsible for placing the most recent SmPC for the brand being used at site in the local pharmacy folder or a file note that makes reference to the electronic source. A trial-specific label will not be used for docetaxel.

Descriptive information for Docetaxel can be found in the package insert. Study treatment with docetaxel should be administered according to the institutional standards at each site. Docetaxel will be used and stored as detailed on the product label and according to manufacturer's instructions. There will be no re-imbursement to sites for docetaxel used during trial treatment.

Accountability of docetaxel is as per local standard practice.

10.4 Granulocyte-Colony Stimulating Factor (G-CSF)

10.4.1 Composition of G-CSF

G-CSF is typically available as a solution for injection/infusion, with varying concentrations commercially available.

10.4.2 Supply, storage and handling of G-CSF

G-CSF is commercially available and should be sourced locally as per standard practice at the investigator sites, using products that have a UK or EU license. As individual sites may use different brands or manufacturers for this drug, each site is responsible for placing the most recent SmPC for the brand being used at site in the local pharmacy folder or a file note that makes reference to the electronic source. A trial-specific label will not be used for G-CSF.

Descriptive information for G-CSF can be found in the package insert. Study treatment with G-CSF should be administered according to the institutional standards at each site. G-CSF will be used and stored as detailed on the product label and according to manufacturer's instructions. There will be no re-imbursement to sites for G-CSF used during trial treatment.

Accountability of G-CSF is as per local standard practice.

11. Treatment Details

11.1 Pre-treatment investigations and tests required

See section 12 for full details of baseline and pre-treatment assessments required following written informed consent, and ongoing clinical review to proceed with each cycle of treatment.

11.2 Nal-IRI/5-FU/folinic acid Treatment

11.2.1 Nal-IRI doses and administration

- Liposomal irinotecan, folinic acid and 5-FU should be administered sequentially.
- The recommended dose and regimen of nal-IRI is 70 mg/m^2 intravenously over 90 minutes (±10 minutes), followed by folinic acid as per local standard practice (recommended dose is 350 mg fixed dose), followed by 5-fluorouracil 2400 mg /m^2 BSA intravenously over 46 hours.*
- Following cycle 1, subsequent doses should be administered every 14 days (+3 days / -1 day).
- Liposomal irinotecan must not be administered as a bolus injection or an undiluted solution. Prior to administration, the appropriate dose of nal-IRI must be diluted with 5% glucose solution for injection

* Sites are permitted to follow local practice guidelines for administration of folinic acid and 5-fluorouracil: dose banding of folinic acid and 5-fluorouracil may be permitted. However, sites should confirm this with the CTRU prior to commencing recruitment.

or sodium chloride 9 mg/ml (0.9%) solution for injection to prepare a solution of the appropriate dose of nal-IRI diluted to a final volume of 500 ml. Mix the diluted solution by gentle inversion. Care should be taken to ensure that any in-line filters used are >5µm and that no other dilutents are used.

- The actual dose of nal-IRI to be administered should be determined by calculating the patient's body surface area at the beginning of each cycle. A ±10% variance in the calculated total dose should be allowed for ease of dose administration.
- Since nal-IRI vials are single-use vials, site staff must not store any unused portion of a vial for future use and they must discard unused portions of the product in accordance with the site procedures for managing cytotoxic agents.

11.2.2 Nal-IRI premedication

It is recommended that patients receive pre-medication for nausea and vomiting prior to nal-IRI infusion with standard doses of dexamethasone (or an equivalent corticosteroid) together with a 5-HT3 antagonist (or other anti-emetic), unless contraindicated for the individual patient. Pre-medication should be given on the day of treatment, starting at least 30 minutes before administration of nal-IRI.

Atropine may be prescribed prophylactically for patients who experience acute cholinergic symptoms in previous cycles. Physicians should also consider providing patients with an antiemetic regimen for subsequent use, as well as loperamide (or equivalent) for treatment of late diarrhoea, if necessary.

11.2.3 5-FU and folinic acid doses and administration

- Folinic acid will be administered as an IV infusion as per local standard practice, every 14 days (+3 days/- 1 day). Folinic acid should be administered prior to the 5-FU infusion. Actual dose of folinic acid to be administered may be determined by the site's local standard practice. It is recommended that a fixed dose of 350 mg is given.
- 5-flourouracil will be administered at a dose of 2400 mg /m^2 BSA intravenously over 46-hours every 14 days (+3 days/-1 day).
- Following cycle 1, subsequent doses should be administered every 14 days (+3 days / -1 day).

Folinic acid and 5-fluorouracil should be reconstituted as per the instructions on the package inset or standard institutional guidelines.

Dose banding of 5-FU is permitted as per local practice. The CTRU should be notified about this prior to the study opening at site and a copy or reference of the dose banding table being used should be supplied to CTRU by site. Patients should be weighed at the start of each cycle. Actual dose of 5-FU (and folinic acid if standard practice at site) to be administered will be determined by calculating the patient's body surface area at Cycle 1. The dose only needs to be re-calculated if the patient's weight changes by +/- 10% from the weight on which the current dose is based.

11.3 Docetaxel treatment

11.3.1 Docetaxel doses and administration

- Docetaxel will be administered at a dose of 75mg/m^2 as an IV infusion over 60 minutes, or as per local standard practice.
- Following cycle 1, subsequent doses should be administered every 21 days (+3 days / -1 day).
- G-CSF will be administered no less than 24 hours following each cycle of docetaxel. The administration of G-CSF utilised should follow local standard practice.

Dose banding of docetaxel is permitted as per local practice. The CTRU should be notified about this prior to the study opening at site and a copy or reference of the dose banding table being used should be supplied to CTRU by site. Patients should be weighed at the start of each cycle. Actual dose of docetaxel to be administered will be determined by calculating the patient's body surface area at Cycle 1. The dose only needs to be re-calculated if the patient's weight changes by +/- 10% from the weight on which the current dose is based.

11.4 Treatment toxicities

Please refer to the relevant SmPC for full details of all treatment toxicities. A summary of the most common toxicities for each IMP is given below. Dose modification recommendations are provided in Section 11.6.

11.4.1 Nal-IRI toxicity

Data from nal-IRI studies do not show any unexpected toxicity when compared to the active ingredient, irinotecan, which has been studied extensively. The warnings and precautions for the use of irinotecan and the recommended treatment procedures for managing those toxicities are provided below. Certain known adverse reactions of irinotecan have not been observed with nal-IRI at the time of writing. This could be due to the limited cumulative patient exposure to date of nal-IRI, or the use of appropriate premedication and early recognition and treatment of expected adverse events. The adverse reactions not observed include anaphylaxis or anaphylactoid reaction, interstitial lung disease-like pulmonary toxicity and acute pancreatitis. There is insufficient evidence to know whether these known adverse reactions of irinotecan are also associated with nal-IRI. Nevertheless, nal-IRI should be discontinued in patients with a confirmed diagnosis of interstitial lung disease.

Please reference the nal-IRI SmPC for additional information on undesirable effects. For recommendations on the management of reactions see Appendix 4. For dose modifications refer to Section 11.6.1.

11.4.1.1 Diarrhoea

Irinotecan/5-fluorouracil can induce both early and late forms of diarrhoea that appear to be mediated by different mechanisms. Early diarrhoea (occurring during or shortly after infusion of irinotecan) is cholinergic in nature. It is usually transient and only infrequently severe. It may be accompanied by symptoms of rhinitis, increased salivation, miosis, lacrimation, diaphoresis, flushing, and intestinal hyper-peristalsis that can cause abdominal cramping. For patients who experienced early cholinergic symptoms during the previous cycle of nal-IRI, prophylactic administration of atropine will be given at the discretion of the investigator.

Late diarrhoea (generally occurring more than 24 hours after administration of irinotecan) can be life-threatening since it may be prolonged and may lead to dehydration, electrolyte imbalance, or sepsis. Late

diarrhoea should be treated promptly with loperamide, and octreotide should be considered if diarrhoea persists after loperamide, as described in Appendix 6 (Algorithm for Diarrhoea Management). Local standard of care practices for chemotherapy-induced diarrhoea can also be used. Loss of fluids and electrolytes associated with persistent or severe diarrhoea can result in life-threatening dehydration, renal insufficiency, and electrolyte imbalances, and may contribute to cardiovascular morbidity. The risk of infectious complications is increased, which can lead to sepsis in patients with chemotherapy-induced neutropenia. Patients with diarrhoea should be carefully monitored, given fluid and electrolyte replacement if they become dehydrated, and given antibiotic support if they develop ileus, fever, or severe neutropenia.

11.4.1.2 Neutropenia

Deaths due to sepsis following severe neutropenia have been reported in patients treated with irinotecan, 5-fluorouracil and nal-IRI. Neutropenic complications should be managed promptly with antibiotic support. Granulocyte colony stimulating factor (GCSF) may be used to manage neutropenia at the investigator's discretion, provided it is administered within parameters specified in Appendix 4 (Management of nal-IRI/5-FU/folinic acid toxicities).

11.4.1.3 Hypersensitivity

Hypersensitivity reactions including severe anaphylactic or anaphylactoid reactions have been observed with irinotecan, but have not been observed with nal-IRI at the time of writing. This could be due to the limited cumulative patient exposure at the time of writing of nal-IRI, or the use of appropriate premedication and early recognition and treatment of expected adverse events. There is insufficient evidence to know whether these known adverse reactions of irinotecan are also associated with nal-IRI. Suspected drugs should be withheld immediately and aggressive therapy should be given if hypersensitivity reactions occur as per local standard of care practice.

11.4.1.4 Colitis/Ileus

Cases of colitis complicated by ulceration, bleeding, ileus, and infection have been observed. Patients experiencing ileus should receive prompt antibiotic support.

11.4.1.5 Thromboembolism

Thromboembolic events have been observed in patients receiving irinotecan-containing regimens; the specific cause of these events has not been determined.

11.4.1.6 Pregnancy

Women of childbearing potential must be advised to avoid becoming pregnant while receiving treatment with chemotherapy. If a pregnancy is reported, treatment must be discontinued. The patient must be withdrawn from treatment, and the pregnancy must be followed until the outcome becomes known.

11.4.1.7 Care of intravenous site

Care should be taken to avoid extravasation, and the infusion site should be monitored for signs of inflammation. Should extravasation occur, flushing the site with sterile saline and applications of ice are recommended, or as per institutional standard of care.

11.4.1.8 Patients at particular risk

In clinical trials of the weekly schedule of irinotecan, it has been noted that patients with modestly elevated baseline serum total bilirubin levels (1.0 to 2.0 mg/dL [17.1umol/L to 34.2umol/L]) have had a significantly greater likelihood of experiencing first-cycle grade 3 or 4 neutropenia than those with bilirubin levels that were less than 1.0 mg/dL (17.1umol/L) (50.0% [19/38] versus 17.7% [47/226]; $p < 0.001$). Patients with abnormal glucuronidation of bilirubin, such as those with Gilbert's syndrome, may also be at greater risk of myelosuppression when receiving therapy with irinotecan. It is not known if this is translated to treatment with liposomal irinotecan. Complete blood counts must be conducted prior to treatment administration for every treatment visit.

11.4.1.9 Acute infusion-associated reactions

Acute infusion-associated reactions characterised by flushing, shortness of breath, facial swelling, headache, chills, back pain, tightness of chest or throat, and hypotension have been reported in a small number of patients treated with liposome drugs. In most patients, these reactions generally resolve within 24 hours after the infusion is terminated. In some patients, the reaction resolves by slowing the rate of infusion. Most patients who experienced acute infusion reactions to liposome drugs are able to tolerate further infusions without complications. Institutional policies or the treatment guidelines provided in Appendix 4 shall be used for the management of infusion reactions.

11.4.1.10 Other toxicity potential

Liposomal irinotecan, the liposomal formulation of irinotecan, is different from irinotecan in un-encapsulated formulation, so there is a potential for toxicities other than those caused by irinotecan. All patients should be monitored closely for signs and symptoms indicative of drug toxicity, particularly during the initial administration of treatment.

11.4.1.11 Special populations

Patients with hepatic impairment

No dedicated hepatic impairment study has been conducted with nal-IRI. Patients with hyperbilirubinaemia had higher concentrations of total SN-38, and therefore the risk of neutropenia is increased. Complete blood counts must be conducted at every treatment visit. It is advised the use of nal-IRI should be avoided in patients with bilirubin beyond 1.5 times the ULN during treatment and if the bilirubin rises above this level, appropriate imaging as per standard of care (e.g. ultrasound liver) should be carried out to outrule irreversible cause for bilirubin rise.

Patients with renal impairment

No dedicated renal impairment study has been conducted with nal-IRI. While a dose adjustment is not recommended, nal-IRI should be used with caution in patients with mild to moderate renal impairment.

Liposomal irinotecan is not recommended for use in patients with severe renal impairment (CrCl < 30 ml/min).

Elderly patients

Forty-six percent of patients treated with nal-IRI in clinical studies were 65 years and older. Overall, no major clinical differences in safety or efficacy were reported between patients 65 years and older and patients less than 65 years of age, although a higher frequency of discontinuation (14.8% vs 7.9%) was noted in the 65 years and older population compared to patients younger than 65 years treated with nal-IRI+5-FU/folinic acid in the pancreatic adenocarcinoma study, while the grade 3 or higher and serious treatment emergent adverse events were seen more frequently in patients younger than 65 years of age (84.1% and 50.8%) compared to patients 65 years and older (68.5% and 44.4%). Conversely, patients older than 75 years (n=12) experienced more frequent serious adverse reactions, dose delay, dose reduction and discontinuation compared to patients 75 years of age and younger (n=105) when treated with nal-IRI+5-FU/folinic acid in the pancreatic adenocarcinoma study.

11.4.2 Nal-IRI, 5-FU and folinic acid combination therapy – potential toxicity

Stomatitis and oesophagopharyngitis (which may lead to sloughing and ulceration), diarrhoea, anorexia, nausea, emesis and leukopenia are commonly seen with treatment; alopecia and dermatitis, in the form of pruritic rash usually appearing on the extremities, may also be seen (see package insert or SmPC).

ECG abnormalities and rarely, myocardial infarction have been reported following administration of 5-FU. Care should be therefore be exercised in treating patients who experience chest pain during courses of treatment and careful consideration should be given to re-administration of 5-FU after a documented cardiovascular reaction (arrhythmia, angina, ST segment changes) as there is a risk of sudden death.

Common adverse events (≥20%) that were observed with nal-IRI in combination with 5-FU/folinic acid in clinical trials considered to be related were: diarrhoea, nausea, vomiting, decreased appetite, neutropenia, fatigue, anaemia, stomatitis and pyrexia.

See Section 11.6 for dose reductions and Appendix 4 for recommendations for the management of toxicities.

11.4.3 Docetaxel toxicity

Listed below are the most common, serious toxicities that may develop in patients treated with docetaxel.

- Hypersensitivity reactions
- Risk of serious infection
- Neutropenia
- Thrombocytopenia
- Anaemia

- Neuropathy
- Hepatic dysfunction
- Cutaneous reaction
- Respiratory disorders
- Stomatitis

Other, common, usually less serious toxicities expected from the use of docetaxel include:

- Nausea and vomiting
- Diarrhoea and Constipation
- Alopecia (Hair loss)
- Muscle and joint pain
- Fluid retention
- Effects on fertility and menstruation

For other toxicities please refer to the current SmPC. For recommendations on the management of reactions see Appendix 5. For dose modifications refer to Section 11.6.3.

11.5 Concomitant Therapies

All concurrent medical conditions and complications of the underlying malignancy will be treated at the discretion of the treating physician according to acceptable local standards of medical care. Patients should receive analgesics, anti-emetics, antibiotics, anti-pyretics, and blood products as necessary. The use of warfarin-type anticoagulant therapies is not permitted.

Guidelines for treating certain medical conditions are discussed in Appendices 4, 5, 6 and 7; however, institutional guidelines for the treatment of these conditions may also be used. The concomitant therapies that warrant special attention are discussed below.

11.5.1 Interactions affecting the use of nal-IRI

Information about drug interactions with nal-IRI is referenced from the published scientific literature for non-liposomal irinotecan. No dedicated drug interaction studies were conducted with nal-IRI.

Please reference the nal-IRI SmPC for additional information.

Strong CYP3A4 Inducers: Patients receiving concomitant non-liposomal irinotecan and CYP3A4 enzyme-inducing anticonvulsants phenytoin, phenobarbital or carbamazepine have substantially reduced exposure to irinotecan (AUC reduction by 12% with St John's wort, 57%-79% with phenytoin, phenobarbital, or carbamazepine) and SN-38 (AUC reduction by 42% with St John's wort, 36%-92% with phenytoin phenobarbital, or carbamazepine).

Strong CYP3A4 inhibitors and UGT1A1 inhibitors: Patients receiving concomitant non-liposomal irinotecan and ketoconazole, a CYP3A4 and UGT1A1 inhibitor, have increased SN-38 exposure by 109%. Therefore, co-administration of nal-IRI with other inhibitors of CYP3A4 may increase systemic exposure of nal-IRI.

See Appendix 8 for a detailed list of inhibitors and inducers of CYP3A. Refer to the current nal-IRI SmPC for further information.

Based on the drug interaction of non-liposomal irinotecan and ketoconazole, co-administration of nal-IRI with other inhibitors of UGT1A1 (e.g. atazanavir, gemfibrozil, indinavir) may also increase systemic exposure of nal-IRI.

Treatment with these agents and any others that interact with irinotecan, should be avoided wherever possible and the planned use of these agents is not permitted at trial entry. However, should it become necessary for trial participants to be treated with a strong CYP3A4 inducer or inhibitor or an inhibitor of UGT1A1, this may be permissible and the CTRU should be notified so that the Chief Investigator can be consulted.

Co-administration of nal-IRI with 5-fluorouracil/folinic acid does not alter the pharmacokinetics of nal-IRI based on the population pharmacokinetic analysis.

11.5.2 Interactions of nal-IRI affecting the use of other drugs

No interaction of nal-IRI with other medicines is known.

11.5.3 Interactions affecting the use of 5-FU and/or folinic acid

5-FU interacts with warfarin, therefore concomitant use is not permitted. Refer to the SmPC of 5-FU and folinic acid for any other drug interactions.

Vaccinations with live viruses should be avoided by patients receiving 5-FU.

11.5.4 Interactions affecting the use of docetaxel

Caution should be exercised when administering docetaxel concomitantly with medicines known to inhibit (e.g. ketoconazole and other imidazole antifungals, erythromycin, fluoxetine, gemfibrozil, cimetidine, ritonavir, saquinavir, indinavir and nelfinavir) or induce (e.g. rifampicin, carbamazepine, phenytoin, efavirenz, nevirapine) either CYP3A4 or CYP3A5.

See Appendix 8 for a detailed list of inhibitors and inducers of CYP3A. The planned use of these agents is not permitted at trial entry. However, should it become necessary for trial participants to be treated with a CYP3A inducer or inhibitor, this may be permissible and the CTRU should be notified so that the Chief Investigator can be consulted.

Refer to the current docetaxel SmPC for further information.

Refer to the relevant, updated SmPC for any other drug interactions between docetaxel and other medications.

11.6 Dose modification requirements

Dosing may be held for up to 28 days from when it was due, to allow for (but not limited to) recovery from toxicity related to the study treatments, infection, following patient request.

In the event of a delay due to toxicity and depending on the toxicity experienced, a dose modification may be required at subsequent cycles following a dose delay, see below for full information relating to each IMP.

If an alteration to treatment schedule is clinically appropriate, this may be permitted at the discretion of the CI or CI delegate.

If a patient's dose is reduced during treatment due to toxicity, it must remain reduced for the duration of treatment; dose should not be re-escalated to an earlier dose.

Patients who have already received two dose reductions and experience further toxicities which would require dose reduction (as defined below) should discontinue study medication. In the event that the participant is deriving clinical benefit and the treating clinician would prefer to continue treatment, a further dose reduction may be permitted at discretion of the CI or CI delegate.

For those patients randomised to the nal-IRI/5-FU/folinic acid arm, all three treatments should be administered during each cycle. However, where it is not possible to administer nal-IRI due to toxicity, 5-FU/folinic acid could be administered as a monotherapy.

If the time required for recovery from toxicity (to ≤grade 2 CTCAE or baseline) is **more than 28 days**, the participant must discontinue trial treatment. The participant should continue to attend 8 weekly clinic visits for CT scans and collection of follow-up data and trial data will continue to be collected unless the participant withdraws consent for further trial interventions and/or data collection.

11.6.1 Nal-IRI dose modifications

For Grade 1 and 2 toxicities, no dose modifications are required.

In the event of Grade 3 or 4 toxicity, the doses of nal-IRI and 5-FU must be reduced as indicated in **Table 6.** Following a dose reduction, subsequent doses of nal-IRI and 5-FU must continue to be adjusted as indicated in **Table 6.** All dose modifications must be based on the worst preceding toxicity.

Table 6 | Dose modifications for nal-IRI and 5-FU and folinic acid, for Grade 3-4 toxicities

	Toxicity<br>CTCAE Grade (value)	Dose Adjustment
**Haematological toxicities**	**Neutropenia**<br>Grade 3 or Grade 4<br>(<1000/mm^3: <1x10^9/L)<br>Or neutropenic fever	**A new cycle of therapy must not begin until the absolute neutrophil count is ≥1.5x10^9/L (Dose modifications below are for subsequent treatments, if grade 3 or 4 neutropenia is recorded on day 1 of a cycle or neutropenic fever is experienced during a cycle) ***<br>First occurrence Reduce nal-IRI dose to 50 mg/m^2<br>Reduce 5-FU dose by 25% (1800 mg/m^2)<br>Second occurrence Reduce nal-IRI dose to 43 mg/m^2<br>Reduce 5-FU dose by 25% (1350 mg/m^2)<br>Third occurrence Discontinue treatment
	**Thrombocytopenia**<br>**Leukopenia**<br>Grade 3 or 4	**A new cycle of therapy must not begin until the platelet count is ≥100x10^9/L**<br>Dose modifications for grade 3 or 4 thrombocytopenia or leukopenia are the same as recommended for neutropenia above for first, second and third recurrence.
**Non-haematological toxicities**	**Diarrhoea**<br>Grade 3 or 4<br>(≥7 stools per day pre-treatment)	**A new cycle of therapy must not begin until diarrhoea resolves to ≤ Grade 1** (2-3 stools/day more than pre-treatment frequency) **(Dose modifications below are for subsequent treatments)**<br>First occurrence Reduce nal-IRI dose to 50 mg/m^2<br>Reduce 5-FU dose by 25% (1800 mg/m^2)<br>Second occurrence Reduce nal-IRI dose to 43 mg/m^2<br>Reduce 5-FU dose by 25% (1350 mg/m^2)<br>Third occurrence Discontinue treatment
	**Nausea/vomiting**<br>Grade 3 or 4 despite optimal antiemetic therapy	**A new cycle of therapy must not begin until nausea/vomiting resolves to ≤ Grade 1 or baseline (Dose modifications below are for subsequent treatments)**<br>First occurrence Optimise antiemetic therapy<br>Reduce nal-IRI dose to 50 mg/m^2<br>Second occurrence Optimise antiemetic therapy

		Reduce nal-IRI dose to 43 mg/m^2<br><br>Third occurrence Discontinue treatment
	**Hepatic, renal, respiratory or other toxicities**<br>Grade 3 or 4<br>(asthenia and grade 3 anorexia do not require dose adjustment and also excluding grade ≥3 ALT/AST which resolve to baseline within 7 days and grade ≥3 toxicities which following case causality assessment are not in the category of 'Certain', 'Probable' or 'Possible' and as such are not related to study treatment, or which are not considered a clinically-significant toxicity)	**A new cycle of therapy must not begin until the adverse reaction resolves to ≤ Grade 1 (Dose modifications below are for subsequent treatments)**<br><br>First occurrence Reduce nal-IRI dose to 50 mg/m^2<br>Reduce 5-FU dose by 25% (1800 mg/m^2)<br><br>Second occurrence Reduce nal-IRI dose to 43 mg/m^2<br>Reduce 5-FU dose by 25% (1350 mg/m^2)<br><br>Third occurrence Discontinue treatment

* Prophylactic use of GCSF can be considered prior to dose modification in those patients who have had at least one episode of grade 3 or 4 neutropenia or neutropenic fever while receiving therapy or have had documented grade 3 or 4 neutropenia or neutropenic fever while receiving prior anti-neoplastic therapy. For the doctaxel arm, prophylactic use of G-CSF is mandated.

11.6.2 5-FU and folinic acid dose modifications

Dose modifications for 5-FU are provided below. No dose adjustments for toxicity are required for folinic acid. Folinic acid must be given immediately prior to each 5-FU dose; hence, if the 5-FU dose is held, folinic acid dose should be held as well. If the dosing of nal-IRI needs to be withheld, then the 5-FU/folinic acid in the combination can be administered as monotherapy. In case a patient experiences an infusion reaction, either institutional guidelines or the guidelines provided for nal-IRI infusion reaction management should be used (see Appendix 7).

11.6.2.1 Haematological toxicities: 5-FU dose modifications

Absolute neutrophil count (ANC) and platelet count must be measured locally **no more than 3 days prior to day 1** of each treatment cycle. Treatment should only proceed if;

- ANC ≥ 1.5x10^9/L
- Platelet count ≥100x10^9/L

In the event of haematological toxicity, treatment must be delayed (up to 28 days) to allow sufficient time for recovery. On recovery, treatment should be administered according to the guidelines provided in **Table 6**.

11.6.2.2 Non-haematological toxicities: 5-FU dose modifications

Treatment must be delayed until all clinically-significant Grade 3 or 4 non-haematological toxicities resolve to Grade 1 or baseline. If delays are greater than 28 days for toxicity, the participant must be withdrawn from trial treatment.

Dose adjustments of other 5-FU-related toxicities are provided below in Table 7. The duration of the cycles is fixed at 2 weeks.

Table 7 | 5-FU dose modifications for other non-haematological toxicities (other than asthenia and grade 3 anorexia and also excluding grade ≥3 toxicities which following case causality assessment are not in the category of 'Certain', 'Probable' or 'Possible' and as such is not related to study treatment, or which are not considered a clinically-significant toxicity).

[a] All dose modifications must be based on the worst preceding toxicity.
[b] Participants who require more than 2 dose reductions should be withdrawn from trial treatment unless agreed with the CI or CI delegate.
[c] Asthenia and Grade 3 Anorexia do not require dose modification.

**Worst toxicity CTCAE Grade**	**5-FU dose for next cycle[a]**
Grade 1 or 2	100% of previous dose, except for Grade 2 hand and foot syndrome, Grade 2 cardiac toxicity, or any grade neurocerebellar toxicity
Grade 2 hand foot syndrome	Reduce dose by 25% [b]
Any grade neurocerebellar or ≥ Grade 2 cardiac toxicity	Discontinue therapy
Grade 3 or 4	Reduce dose by 25% [b], except for Grade 3 or 4 hand foot syndrome
Grade 3 or 4 hand foot syndrome	Discontinue therapy

11.6.2.3 Other toxicity requiring special attention

Corrected QT interval (QTc) prolongation that occurs in the setting of diarrhoea-induced electrolyte imbalance should be treated with appropriate electrolyte repletion. Once the underlying abnormality is corrected and the ECG abnormalities have reversed, treatment may continue under careful monitoring, and with appropriate dose modification for diarrhoea as per local standard of care practice.

11.6.3 Docetaxel dose modifications

Neutrophil and platelet count must be measured locally **no more than 3 days prior to day 1** of each treatment cycle. Treatment must only proceed if;

- ANC ≥ $1.5x10^9$/L
- Platelet count ≥$100x10^9$/L
- Bilirubin ≤1.5 x ULN
- ALT and/or AST ≤2.5 x ULN (in absence of liver metastasis) or ≤5 x ULN (in presence of liver metastasis)

In the event of haematological toxicity, treatment must be delayed (up to 28 days) to allow sufficient time for recovery. Guidelines for docetaxel dose modifications are provided below in Table 8.

Table 8 | Dose reductions for docetaxel toxicities

**Toxicity**	**Severity**	**Management**
**Hypersensitivity**	Grade 3/Grade 4	Administration of appropriate medication (see below).
**Neutropenia**	Day 1 neutrophil count <1500/mm^3: <$1.5x10^9$/L<br><br>Febrile neutropenia OR Prolonged Grade 4 neutropenia (Neutrophil count <500/mm^3: <$0.5x10^9$/L for 7 days or more)	Stop treatment until neutrophils recovers to at least $1.5x10^9$/L. If neutrophils <$1.5x10^9$/L for ≤7 days, restart docetaxel at full dose (75mg/m^2). If neutrophils <$1.5x10^9$/L for >7 days, restart docetaxel at 55mg/m^2 or next lowest dose level (40mg/m^2) if already reduced.*<br><br>Stop treatment until neutrophils ≥ $1.5x10^9$/L. Restart drug at 55mg/m^2 or next lowest dose level (40mg/m^2) if already reduced.*
**Neuropathy**	Grade 3/Grade 4	Stop docetaxel treatment.

**Thrombocytopenia**	Platelet count < 100 x 10^9/L<br><br>Platelet count < 50 x 10^9/L (Grade 3/Grade 4)	Stop treatment until platelets ≥ $100x^9$/L. Restart drug at full dose (75mg/m^2).<br><br>Stop treatment until platelets ≥ $100x^9$/L. Restart drug at 55mg/m^2 or next lowest dose level (40mg/m^2) if already reduced.
**Hepatic Dysfunction**	Bilirubin > 1.5 ULN<br>ALT/AST>2.5xULN (in absence of liver metastasis), > 5 x ULN (in presence of liver metastasis)	Stop docetaxel until parameters recover to baseline levels. Restart drug at 55mg/m^2 or next lowest dose level (40mg/m^2) if already reduced.
**Cutaneous reaction**	Grade 2<br><br>Severe or cumulative (Grade 3/Grade 4)	Stop treatment until recovery to Grade 1 or better. Restart drug at full dose (75mg/m^2).<br><br>Stop treatment until recovery (Grade 1 or better). Restart drug at 55 mg/m^2 or next lowest dose level (40mg/m2) if already reduced.
**Other non-haematological toxicity (excluding grade ≥3 toxicities which following case causality assessment are not in the category of 'Certain', 'Probable' or 'Possible' and as such is not related to study treatment, or which are not considered a clinically-significant toxicity)**	Grade 3/Grade 4	Stop docetaxel until parameters recover to baseline levels. Restart drug at 55mg/m^2 or next lowest dose level (40mg/m^2) if already reduced.

*For the docetaxel arm, prophylactic use of G-CSF is mandated.

Patients who have already received two dose reductions and experience further toxicities which would require dose reduction should discontinue study medication. In the event that the participant is deriving clinical benefit and the treating clinician would prefer to continue treatment, a further dose reduction may be permitted at discretion of the CI or CI delegate.

11.6.5 Management of infusion reactions

Infusion reactions will be defined according to the National Cancer Institute Common Terminology Criteria for Adverse Events (CTCAE) (Version 5.0) definitions of an allergic reaction or anaphylaxis. Institutional

policies or the treatment guidelines provided in Appendix 7 shall be used for the management of infusion reactions.

11.7 Withdrawals

In line with usual clinical care, cessation or alteration of regimens at any time will be at the discretion of attending clinicians or the participants themselves. Participants should be withdrawn from trial treatment in the event of a delay of more than 28 days for any reason. Participants may also be withdrawn from trial treatment in the event of development of any condition or occurrence of any event, which, in the opinion of the local investigator, justifies discontinuation of treatment, or patient request.

The Withdrawal CRF should be completed and faxed or sent via secure email to the CTRU **within 24 hours** of the research team becoming aware of a participant discontinuing trial treatment.

All participants withdrawn from trial treatment for reasons other than disease progression should continue to have 8-weekly CT scans until diagnosis of disease progression, if appropriate. The Follow-Up CRF should be completed at 8-weekly visits that coincide with the CT scans and should be collected until diagnosis of disease progression or 6 months after the last participant is randomised. Blood samples for serum enolase measurement should continue to be collected until disease progression.

Quality of life questionnaires should continue to be completed every 6 weeks by participants who have withdrawn from trial treatment but whose disease has not yet progressed radiologically. In the event that a QoL questionnaire is not administered in clinic (for those participants who have not progressed or withdrawn consent for this data collection), the local research team should send the questionnaire out by post after checking the participant's status and establishing it is appropriate to do so. Quality of life questionnaires will be requested from all participants until death, disease progression or 6 months after the last participant is randomised, whichever comes first.

The PI, or delegate should make every effort to ensure that the specific wishes of any participant who wishes to withdraw consent for further involvement in the trial are defined and documented using the Withdrawal CRF, in order that the correct processes are followed by the CTRU and site following the withdrawal of consent.

It should be made clear to any participant specifically withdrawing consent for further data collection that data pertaining to safety will continue to be collected for regulatory reporting purposes and will be included in any safety analysis. In addition, it is suggested that the participant is made aware of the fact that if any significant new information becomes available in regard to the treatment they have received in the trial, it may be necessary to contact them in the future.

12. Assessments, Samples and Data Collection

Data will be collected using paper CRFs, the electronic templates for which will be provided by the CTRU and upon completion should be returned to the CTRU at the University of Leeds. Participating hospitals will be expected to maintain a file of essential trial documentation (Investigator Site File), which will be provided by the CTRU, and keep copies of all completed CRFs for the trial in the Investigator Site File.

12.1 Schedule of Events

The timing of interventions and assessments required for NET-02 are summarised in Table 9 (docetaxel arm) and Table 10 (nal-IRI/5-FU/folinic acid arm).

Table 9 | Schedule of events for Docetaxel

**Week**				**0**	**3**	**6**	**8**	**9**	**12**	**15**	**16**	**18**	**21**	**24**		**28 days post ET[*]**	**PD**
**3 weekly on-study review**			**n/a**		**1st**	**2nd**		**3rd**	**4th**	**5th**		**6th**	**7th**	**8th**	***n***		
**Timepoint**		**Pre-rand**	**Baseline**	**C1D1**	**C2D1**	**C3D1**		**C4D1**	**C5D1**	**C6D1**		**C7D1**	**C8D1**	**C9D1**	**C*n***		
Clinical Procedures/ Assessments	ECG[†]	**x**															
	CT or MRI scan (RECIST 1.1)	**x**	**x**[3]				**x**				**x**			**x**	**x**[ø]		
	ECOG performance status	**x**		**x**	**x**	**x**		**x**	**x**	**x**		**x**	**x**	**x**	**x**		
	Medical history and baseline symptoms	**x**															
	Physical assessment		**x**	**x**	**x**	**x**		**x**	**x**	**x**		**x**	**x**	**x**	**x**		
	Height		**x**														
	Weight, vital signs		**x**	**x**	**x**	**x**		**x**	**x**	**x**		**x**	**x**	**x**	**x**		
	AE assessment				**x**	**x**		**x**	**x**	**x**		**x**	**x**	**x**	**x**	**x**	
	Concomitant medications	**x**	**x**	**x**	**x**	**x**		**x**	**x**	**x**		**x**	**x**	**x**	**x**		
Laboratory assessments	Pregnancy test	**x**															
	Blood samples for translational research [‡]		**x**			**x**											**x**
	Blood sample for neuron-specific enolase measurement		**x**			**x**			**x**			**x**		**x**	**x**[¥]		**x**
	FBC, biochemistry, estimation of renal function[§]	**x**		**x**	**x**	**x**		**x**	**x**	**x**		**x**	**x**	**x**	x		
Data collection	QoL questionnaires (EORTC QLQ-C30 & GI.NET21)	**x**				**x**			**x**			**x**		**x**	**x**[¥]		**x**
	CRF completion		**x**	**x**	**x**	**x**	**x**	**x**	**x**	**x**	**x**	**x**	**x**	**x**	**x**	**x**	**x**

---

[*] This may be completed in clinic or over the telephone 30 days (+/- 1 week) following permanent end of treatment (ET).
[†] Following informed consent and registration, and prior to randomisation (if not already completed as part of standard care).
[3] CT thorax/abdomen/pelvis within 28 days prior to starting treatment. If the pre-randomisation eligibility CT scan is within 28 days of starting treatment then a repeat scan is not required.
[‡] Blood samples for translational research will be drawn at baseline, at 6 weeks and at progression. Blood samples for development of mouse model of NEC may be taken at the Christie only (where consent is in place).
[§] Including full blood count (Hb, WBC, ANC, Platelet count), biochemistry (Sodium, potassium, magnesium, urea, creatinine, albumin, adjusted calcium, serum bilirubin, alkaline phosphatase, AST and/or ALT, LDH), estimation of renal function (using Cockcroft-Gault or Wright formula).
[ø] CT or MRI scans should continue at 8 weekly intervals (+/- 1 week) until disease progression or end of trial.
[¥] QoL questionnaires and blood samples for neuron-specific enolase measurement should continue at 6 weekly intervals (+/- 1 week) until disease progression or end of trial.

Table 10 | Schedule of events for nal-IRI/5-FU/folinic acid

Week				0	2	4	6	8	10	12	14	16	18	20	22	24		28 days post ET[*]	PD
2 weekly on-study review			n/a		1st	2nd	3rd	4th	5th	6th	7th	8th	9th	10th	11th	12th	*n*		
Timepoint		Pre-rand	Base line	C1D1	C2D1	C3D1	C4D1	C5D1	C6D1	C7D1	C8D1	C9D1	C10D1	C11D1	C12D1	C13D1	C*n*		
Clinical Procedures/ Assessments	ECG[†]	x																	
	CT or MRI scan (RECIST 1.1)	x	x[3]					x				x				x	x[ø]		
	ECOG performance status	x		x	x	x	x	x	x	x	x	x	x	x	x	x	x		
	Medical history and baseline symptoms	x																	
	Physical assessment		x	x	x	x	x	x	x	x	x	x	x	x	x	x	x		
	Height		x																
	Weight, vital signs		x	x	x	x	x	x	x	x	x	x	x	x	x	x	x		
	AE assessment				x	x	x	x	x	x	x	x	x	x	x	x	x	x	
	Concomitant medications	x	x	x	x	x	x	x	x	x	x	x	x	x	x	x	x		
Laboratory assessments	Pregnancy test	x																	
	Blood samples for translational research [‡]		x				x												x
	Blood sample for neuron-specific enolase measurement		x				x			x			x			x	x[¥]		x
	FBC, biochemistry, estimation of renal function[§]	x		x	x	x	x	x	x	x	x	x	x	x	x	x	x		
Data collection	QoL questionnaires (EORTC QLQ-C30 & GI.NET21)	x					x			x			x			x	x[¥]		x
	CRF completion		x	x	x	x	x	x	x	x	x	x	x	x	x	x	x	x	x

[*] This may be completed in clinic or over the telephone 30 days (+1 week) following permanent end of treatment (ET).
[†] Following informed consent and registration, and prior to randomisation (if not already completed as part of standard care).
[3] CT thorax/abdomen/pelvis within 28 days prior to starting treatment. If the pre-randomisation eligibility CT scan is within 28 days of starting treatment then a repeat scan is not required.
[‡] Blood samples for translational research will be drawn at baseline, at 6 weeks and at progression. Blood samples for development of mouse model of NEC may be taken at the Christie only (where consent is in place).
[§] Including full blood count (Hb, WBC, ANC, Platelet count), biochemistry (Sodium, potassium, magnesium, urea, creatinine, albumin, adjusted calcium, serum bilirubin, alkaline phosphatase, AST and/or ALT, LDH), estimation of renal function (using Cockcroft-Gault or Wright formula).
[ø] CT or MRI scans should continue at 8 weekly intervals (+/- 1 week) until disease progression or end of trial.
[¥] QoL questionnaires and blood samples for neuron-specific enolase measurement should continue at 6 weekly intervals (+/- 1 week) until disease progression or end of trial.

12.2 Eligibility and baseline assessments

Participants must be screened and have given written informed consent before any trial-specific procedures are performed and the participant is randomised.

For patients who do not go on to be registered, details should be added to the Screening Log (see Section 9.2). All patients who consent to trial participation must be registered.

For participants who are registered into the trial but do not go on to be randomisation, details should be recorded on Non-Randomisation Form.

The PI, or, where delegated by the PI, other medically qualified doctors who are trained in Good Clinical Practice (GCP), and are authorised on the trial delegation log should make the assessment and clearly document in the medical notes or CRF that patient is eligible for randomisation.

If, following randomisation, a participant is found to be in breach of the eligibility criteria, the Protocol Violations CRF should be completed and returned (by fax or secure email) to CTRU immediately.

The following assessments must be made to assess eligibility for trial participation:

- Histological confirmation of inoperable, poorly-differentiated, extra-pulmonary Grade 3 NEC.
- Radiological evidence of disease progression following first-line platinum-based chemotherapy and ≥4 weeks from discontinuation of previous chemotherapy regimen <u>or</u> discontinuation of first-line platinum-based chemotherapy due to platinum intolerance.
- Evidence of measurable disease by RECIST v1.1.
- Medical history including all pertinent prior medical conditions, treatments for neuroendocrine carcinoma, surgery or other medical procedures, ongoing significant symptoms relating to prior treatment.
- The Eastern Cooperative Oncology Group (ECOG) Performance Score should be obtained by the treating physician by questioning the patient about their functional capabilities (see **Appendix 2**).
- Details of concomitant medication.
- Screening for COVID-19 will be as per the local site standard operating policy. It is strongly recommended that patients are screened for current COVID-19 infection. Patients with active COVID-19 infection will not be eligible.

The following eligibility assessments should be carried out <u>following written informed consent and registration</u> into the trial:

- Pregnancy test in women of childbearing potential.
- A 12-lead electrocardiogram.

The following eligibility assessments should be carried out <u>no more than 14 days prior to randomisation</u>:

- Full blood count (Hb, WBC, ANC, Platelet count).
- Biochemistry; sodium, potassium, magnesium, urea, creatinine, albumin, adjusted calcium, serum bilirubin, alkaline phosphatase (ALP), AST and/or AST, lactate dehydrogenase (LDH).

- Estimation of renal function (using the Cockroft-Gault formula (as defined in Appendix 3, Wright formula may be used in place of Cockroft-Gault if this is usual local practice)). If the calculated creatinine clearance is less than 30 ml/min, glomerular filtration rate (GFR) may be assessed using either Cr51-EDTA or 99mTc-DTPA clearance method to confirm if GFR is ≥ 30 ml/min).
- Resting blood pressure measurement. It is recommended that subjects should have a systolic blood pressure of either less than 150 mmHG, and/or a diastolic blood pressure of less than 100 mmHg at rest (average of 3 consecutive readings 3-5 minutes apart). Anti-hypertensive drugs may be used to achieve these values.

Where more than 14 days have elapsed since the initial eligibility bloods, these must be repeated prior to randomisation. If these repeated assessments show that the participant is now ineligible, an F20 Non-Randomisation Form should be completed and sent to the CTRU.

Baseline quality of life questionnaires (EORTC QLQ-C30 and EORTC QLQ-GI.NET21) should be completed after registration, once all other screening investigations are successfully completed but **before** meeting with the clinician/randomisation.

Once randomised into the trial, the participant will be assessed by a member of the research team and the following baseline assessments will be carried out:

- Medical history, patient demographics and baseline symptoms.
- Physical examination including a careful assessment of all body systems, including the skin; central and peripheral nervous system; eyes; ears, nose and throat; respiratory, musculoskeletal and cardiovascular systems; abdomen and extremities. Particular attention should be paid to areas of possible neoplastic involvement.
- Vital signs include height, weight, pulse, respiratory rate and temperature.
- Computed tomography (CT) scan (or MRI scan, if appropriate) of thorax-abdomen-pelvis and staging **≤ 28 days of starting trial treatment**. If the pre-randomisation eligibility CT scan is within 28 days of starting treatment then a repeat scan is not required.
- One 10ml blood sample for local measurement of neuron-specific enolase.
- Two 10ml blood samples will be taken from all trial participants for central analysis translational research.
- For participants recruited at The Christie NHS Foundation Trust only, a 10ml blood sample may be taken for mouse model development. This sample may be taken at baseline or during trial participation, timing to be confirmed with CTRU.
- Confirmation of availability of archival paraffin-embedded tissue for translational research.

12.3 Treatment cycle assessments

Participants on the nal-IRI/5-FU/folinic acid arm will have 2-weekly treatment cycles. Participants on the docetaxel arm will have 3-weekly treatment cycles. Participants continue on trial treatment until diagnosis

of disease progression or intolerable toxicity or until 6 months after the last participant is randomised (see Section 11.9 for full list).

12.3.2 Assessments at cycle 1

The following assessments will be carried out **no more than 3 days prior to day 1** of the treatment cycle:

- Local laboratory assessments:
  - Full blood count (Hb, WBC, ANC, Platelet count)
  - Biochemistry: sodium, potassium, magnesium, urea, creatinine, albumin, adjusted calcium, serum bilirubin, alkaline phosphatase (ALP), AST and/or ALT, lactate dehydrogenase (LDH).
- Clinical evaluation, as per local standard practice.
- Weight in kilograms (in light indoor clothing and without shoes).
- Vital signs including resting blood pressure, pulse, respiratory rate and temperature.
- ECOG performance status.
- Physical examination including a careful assessment of all body systems, including the skin; central and peripheral nervous system; eyes; ears, nose and throat; respiratory, musculoskeletal and cardiovascular systems; abdomen and extremities. Particular attention should be paid to areas of possible neoplastic involvement.
- Details of concomitant medications.

12.3.2 Assessments at cycle 2 and onwards

The following assessments will be carried out **no more than 3 days prior to day 1** of all subsequent treatment cycles:

- Local laboratory assessments:
  - Full blood count (Hb, WBC, ANC, Platelet count)
  - Biochemistry: sodium, potassium, magnesium, urea, creatinine, albumin, adjusted calcium, serum bilirubin, alkaline phosphatase (ALP), AST and/or ALT, lactate dehydrogenase (LDH).
- Clinical evaluation, as per local standard practice.
- Weight in kilograms (in light indoor clothing and without shoes).
- Vital signs including resting blood pressure, pulse, respiratory rate and temperature.
- ECOG performance status.
- Physical examination including a careful assessment of all body systems, including the skin; central and peripheral nervous system; eyes; ears, nose and throat; respiratory, musculoskeletal and cardiovascular systems; abdomen and extremities. Particular attention should be paid to areas of possible neoplastic involvement.
- Details of concomitant medications.
- Toxicity assessment: collection of any adverse events that have occurred since the start of the previous treatment cycle.

12.3.3 Assessments at 6-weekly timepoints

- The following assessment should be carried out <u>at 6 weeks</u> (+/- 1 week) post treatment start (approximately cycle 4, day 1 only for nal-IRI/5-FU/folinic acid arm and cycle 3, day 1 only for

docetaxel arm):
- Two 10ml blood samples will be taken from all trial participants for central analysis translational research.

- The following assessments should be carried out at <u>6-weekly intervals</u> (+/- 1 week) post treatment start date (approximately on day 1 of cycles 4, 7, 10, etc. for nal-IRI/5-FU/folinic acid arm and day 1 cycles 3, 5, 7, etc. for docetaxel arm):
  - One 10ml blood sample for local measurement of neuron-specific enolase.
  - QoL questionnaires (EORTC QLQ-C30 and EORTC QLQ-GI.NET21) should be administered to the participant. Questionnaires should be completed by participants at the time of clinical assessment, but before discussion of the outcome of any medical assessments or blood tests, wherever possible.

12.3.4 Assessments at 8 weekly timepoints

- CT or MRI scans will be carried out every 8 weeks (±7 days) from treatment start until diagnosis of disease progression or until 6 months after the last participant is randomised, whichever comes first. Imaging should be reported according to RECIST guidelines V1.1 (see Appendix 1). All follow-up scans should use the same modality as the baseline scan (CT or MRI). Where CT scans are used, a contrast CT scan (chest abdomen pelvis) is the preferred modality of cross sectional imaging. If this is not possible (e.g. in the case of contrast allergy or renal insufficiency), then a non-contrast CT (chest abdomen pelvis) scan should be performed, assuming the disease is evaluable by this method. If the disease is not evaluable using a non-contrast CT scan, then a MRI scan of the abdomen and pelvis and a non-contrast CT scan of the chest should be performed. In addition, other radiographic or scintigraphic procedures, as deemed appropriate by the treating physician, may be performed to assess sites of neoplastic involvement. All subsequent follow-up scans should be the same modality and performed using the same technique and should be reported in accordance with RECIST V1.1. If a patient stops treatment due to intolerance and without progression, every effort should be made to continue CT evaluation every 8 weeks until diagnosis of disease progression or until 6 months after the last participant is randomised, whichever comes first.

12.4 Assessments at disease progression

- Two 10ml blood samples will be taken from all trial participants for central analysis translational research.
- One 10ml blood sample for local measurement of neuron-specific enolase.
- Quality of life questionnaires (EORTC QLQ-C30 and EORTC QLQ-GI.NET21) should be administered to the participant. Questionnaires should be completed by participants at the time of clinical assessment, but before discussion of the outcome of any medical assessments or blood tests, wherever possible.

12.5 Assessment following end of the trial interventional phase

- On the date 6 months after the last participant is randomised, the interventional phase of the trial is complete and all trial participants will cease receiving trial treatment for trial purposes. Participants who continue to receive the same IMPs as those included in the trial do so outside

the remit of the trial, under the auspices of compassionate use.

- Toxicity assessment: collection of any adverse events which may have occurred over the last 28 days following discontinuation of trial treatment (for those participants continuing IMPs under compassionate use, this is considered to be the end of the treatment cycle).

12.6 End of Trial Treatment

Participants should continue on trial treatment until diagnosis of disease progression, intolerable toxicity or until 6-months after the last participant is randomised, whichever comes first.

Participants should be withdrawn from trial treatment in the event of a delay of more than 28 days for any reason.

Participants may also be withdrawn from trial treatment in the event of development of any condition or occurrence of any event, which, in the opinion of the local investigator, justifies discontinuation of treatment, or patient request.

The Withdrawal CRF should be completed and faxed or sent via secure email to the CTRU **within 24 hours** of the research team becoming aware of a participant discontinuing trial treatment.

All participants withdrawn from trial treatment should continue to have 8-weekly CT scans until diagnosis of disease progression. The Follow-Up CRF should be completed at 8-weekly visits that coincide with the CT scans and should be collected until diagnosis of disease progression.

Quality of life questionnaires should continue to be completed every 6 weeks by participants who have withdrawn from trial treatment but have not yet progressed. In the event that a QoL questionnaire is not administered in clinic, the local research team should send the questionnaire out by post after checking the participant's status and establishing it is appropriate to do so. Quality of life questionnaires should continue to be administered until death, disease progression or 6 months after the last participant is randomised, whichever comes first.

Blood samples for neuron-specific serum enolase measurement should continue to be taken from those participants who have withdrawn from trial treatment but have not yet progressed.

12.7 Disease progression

The date of disease progression is defined as the date of the CT or MRI scan by which disease progression is identified. Imaging should be reported according to RECIST guidelines V1.1 (see Appendix 1).

A CT/magnetic resonance imaging (MRI) brain scan is only required if brain metastases are clinically suspected.

12.8 Quality of Life

Participant-reported QoL is assessed using the EORTC QLQ-C30 in combination with the QlQ-GINET-21. Quality of life questionnaires should be completed by all participants at the point of randomisation and then at 6-weekly intervals (day 1 of cycles 3, 5, 7, 9 etc. in the Docetaxel arm/cycle 4, 7, 10, 13 etc. in the nal-IRI arm) and on disease progression. Quality of life questionnaires should be completed from randomisation until diagnosis of disease progression, or until 6-months after the last participant is randomised, whichever comes first.

Quality of life questionnaires should be completed by participants at the time of clinical assessment, but before discussion of the outcome of any medical assessments or blood tests wherever possible.

If a treatment cycle becomes delayed, the QoL assessments should be delayed in line with the treatment cycle. If a participant stops trial treatment for reasons other than diagnosis of disease progression, QoL questionnaires should continue to be completed where ever possible, unless the participant has withdrawn consent for this data to be collected.

Participants will be asked to seal the questionnaires in pre-supplied envelopes prior to being given to research staff. Research staff will then send the sealed envelopes to the CTRU for entry into the database.

In the event that a QoL questionnaire is not administered in clinic, the local research team will send the questionnaire out by post after checking the participant's status and establishing it is appropriate to do so.

12.9 Blood sample analysis

One 10 ml blood sample for the local measurement of neuron-specific enolase will be collected at baseline, at 6-weekly intervals following the start of trial treatment and at diagnosis of disease progression.

Two 10 ml blood samples will be taken for central analysis for translational research at baseline, 6 weeks post-treatment start and at diagnosis of disease progression for all participants. These samples will be analysed for the purpose of quantification and molecular characterisation (through Next Generation Sequencing) of circulating tumour cells and circulating tumour DNA to identify any correlation with disease-related outcomes. One 10 ml blood sample should be collected in a CellSave Preservative tube and one 10 ml blood sample should be collected in a Streck Cell-Free DNA BCT tube. These blood samples should be stored at room temperature and dispatched to the central laboratory at the CRUK Manchester Institute. The samples should be dispatched within 24 hours of collection.

Please refer to trial-specific manual for full details on the handling and dispatch of blood samples.

For participants recruited at The Christie NHS Foundation Trust only, one 10ml blood sample may be taken for mouse model development. This sample may be taken at baseline or during trial participation, timing to be confirmed with CTRU.

It is the responsibility of the trial site to ensure that samples are appropriately labelled in accordance with the trial procedures to conform with the 2018 General Data Protection Regulation. Biological samples collected from participants as part of this study will be transported, stored, accessed and processed in

accordance with national legislation relating to the use and storage of human tissue for research purposes, and such activities shall at least meet the requirements as set out in the 2004 Human Tissue Act.

Participant-specific archival tumour tissue will also be requested and collected (where available) after the trial has closed to recruitment. This tissue will be used for potential future exploratory research into factors that may influence the development of agents to treat this diagnosis and/or response to treatment (where response is defined broadly to include efficacy, tolerability or safety).

Molecular imaging in a defined number of patients may also be considered, dependent on future funding and ethical approval.

12.10 Adverse Events and Serious Adverse Events

Adverse Events (AEs) will be collected at day 1 of each treatment cycle from cycle 2 onwards and will be collected from randomisation until 28 days post trial treatment end date (see Section 12.6 for the end of trial treatment definition). Adverse events will be collected on the Treatment CRF. These should be reported via the standard data management routes and expedited reporting is not required.

Serious Adverse Events (SAEs), Serious Adverse Reactions (SARs) and Suspected Unexpected Serious Adverse Reactions (SUSARs) will be collected from registration until 28 days after the last trial IMP was administered.

For all SAEs, occurring in the trial, an SAE Report CRF must be completed and faxed or sent via secure email to the CTRU **within 24 hours** of the site becoming aware of the event (see Pharmacovigilance Section 14).

For all Suspected Unexpected Serious Adverse Reactions (SUSARs), a SUSAR Report Form should be completed and faxed to the CTRU **within 24 hours** of becoming aware of the event (see section 14).

12.11 Pregnancies

If a patient becomes pregnant, the patient must be withdrawn from trial treatment immediately. All pregnancies and suspected pregnancies of a participant, or of a male participant's partner, occurring from the date of randomisation to 28 days following **permanent** cessation of trial treatment must be reported. Pregnancies should be reported to the CTRU using the 'Notification of Pregnancy' CRF **within 24 hours** of the participating site becoming aware of the pregnancy or suspected pregnancy.

The CTRU will report all pregnancies occurring during treatment to Servier along with any follow-up information. All information passed on to Servier will be anonymised.

All pregnancies will be followed-up until the outcome is known.

12.12 Deaths

All deaths occurring from randomisation until 6 months after the last participant is randomised will be collected. Deaths must be recorded on the Notification of Death Form CRF and sent to the CTRU within 5 days of the site research team becoming aware of the death.

At the end of the trial, sites will be contacted to provide data on any subsequent deaths and survival data.

12.12.1 Treatment-related deaths

In addition to completing a Notification of Death CRF, suspected treatment-related deaths must be notified to the CTRU via the Notification of Death Form (in accordance with section 14 Pharmacovigilance) **within 24 hours** of the site becoming aware.

12.13 End of trial

The end of trial is defined as the date of the collection of the last participant's last data item.

12.14 Trial data and documentation held at sites

Participating sites will be expected to maintain a file of essential trial documentation (Investigator Site File), which will be provided by the CTRU. Participating sites will also be expected to keep copies of all completed CRFs.

Each research site is responsible for maintaining source data for their trial patients in their medical notes, and for transcribing this data onto a trial-specific paper CRF, which will be provided by the CTRU in the form of an electronic booklet.

All entries on the CRF, including corrections, must be made by an authorised member of trial staff. Research site staff will also provide trial patients with copies of the relevant quality-of-life questionnaires for completion at each required time-point.

Research sites will submit original completed copies of the CRF and patient completed QoL questionnaires to the CTRU, within two weeks of the data being collected. A number of CRFs require expedited reporting to the CTRU:

- SAE Report
- SUSAR Report
- Withdrawal Notification
- Death Notification
- Pregnancy Notification
- End of Treatment

Only the participant's trial number, date of birth and initials will be added to the CRFs. Site staff are responsible for ensuring the CRFs returned to CTRU do not contain any other personal identifiable data.

It is the responsibility of the site to ensure that copies of clinical reports are anonymised prior to sending to CTRU: all personal identifiable data should be obliterated and trial number plus date of birth and initials should be used to identify the participant (as well as any other required identifiers, e.g. histopathology number).

Following receipt of the completed CRFs, the CTRU will contact sites on a regular basis to resolve any missing or discrepant data.

It is the responsibility of the site to ensure all photocopies of the completed CRFs are appropriately maintained at site during the trial (including any amendments) and archived according to the Sponsor's requirements at the end of the trial (see section 21.1 on archiving).

12.15 Protocol deviations and violations

The CTRU undertake to adopt all reasonable measures to record data in accordance with the protocol. Under practical working conditions, however, some minor variations may occur due to circumstances beyond the control of the CTRU. All such deviations will be documented on the study records, together with the reason for their occurrence. Where appropriate, deviations will be detailed in the published report.

13. Safety Monitoring Plan

See Appendix 9.

14. Pharmacovigilance

14.1 General Definitions

Adverse event (AE) | An adverse event is any untoward medical occurrence in a patient or clinical trial subject administered a medicinal product or trial-specific treatment and which does not necessarily have a causal relationship with this treatment. An AE can therefore be any unfavourable and unintended sign (including an abnormal laboratory finding for example), symptom or disease temporarily associated with the use of the medicinal product, whether or not considered related to the trial treatment.

Adverse reaction (AR) | An adverse reaction is any untoward and unintended responses to trial IMP related to any dose administered. This definition also covers adverse reactions resulting from medication errors and uses outside what is foreseen in the protocol, including misuse and abuse of the product. This definition implies a reasonable possibility of a causal relationship between the event and the trial treatment. This means there are facts (evidence) or arguments to suggest a causal relationship.

Serious adverse event (SAE)| An adverse event in a trial subject that;

- Results in death.
- Is life-threatening.

- Requires in-patient hospitalisation or prolongation of existing hospitalisation.
- Results in persistent or significant disability/incapacity.
- Is a congenital anomaly/birth defect.
- Is considered an important medical event.

These characteristics/consequences have to be considered at the time of the event. For example, regarding a life-threatening event, this refers to an event in which the subject was at risk of death at the time of event. It does not refer to an event which hypothetically might have caused death if it were more severe. Medical and scientific judgement must be exercised in deciding whether an event is 'serious' in accordance with these criteria.

Serious adverse reaction (SAR)| Reference is made to the above criterion of 'Seriousness' above in relation to SAE. When an SAE is deemed to have been related to the trial IMP and the nature and severity is consistent with the reference safety information (SmPC), the event is termed as a serious adverse reaction.

Suspected unexpected serious adverse reaction (SUSAR)| Any adverse reaction that is serious and the nature and severity is *not* consistent with the reference safety information (SmPC).

The term 'severity' is used here to describe the intensity of a specific event. This has to be distinguished from the word 'serious'. Reports which add significant information on the specificity, increase of occurrence or severity of a known, already documented serious adverse reaction constitute unexpected events.

14.2 Reporting Requirements

Adverse events will be collected for all participants and will be evaluated for intensity and causal relationship with the trial medication or other factors according to CTCAE Version5.0. A copy is provided in the NET-02 Investigator Site File and may also be obtained at:

http://ctep.cancer.gov/protocolDevelopment/electronic_applications/ctc.htm

Published date: May 28, 2009

14.2.1 Adverse Events (AEs)/Adverse Reactions (ARs)

The following specified adverse events (of any grade) that occur **between randomisation and 28 days post end of trial treatment** must be recorded in the patient notes and on the Treatment CRF:

- Anaemia
- Anorexia
- Constipation
- Diarrhoea
- Fatigue
- Nausea
- Neuropathy
- Neutropenia

- Pain
- Peripheral motor neuropathy
- Peripheral sensory neuropathy
- Pyrexia
- Thrombocytopenia
- Vomiting

Adverse events other than those specified above are only collected if the event is CTCAE grade 3 or above.

Adverse events will be recorded at every treatment cycle.Every effort should be made to maintain a complete record of AEs. However, it is recognised that full documentation of AEs may not be possible in the case of patients no longer attending follow-up visits because they are in the end stages of the disease and receiving the appropriate hospice or home care. For patients at this stage of their disease, the pattern of AEs is expected to be similar between the two arms.

Adverse Events will be followed up until 28 days post end of trial treatment.

14.2.2 Serious Adverse Events (SAEs)

All adverse events that meet the definition of 'serious' (as defined in section 14.1) and occur **between registration and 28 days post end of trial treatment** must be recorded in the patient notes and must be reported to the CTRU via email using SAE/SUSAR Report CRF **within 24** hours of the trial site team becoming aware of the event meeting the definition of serious.

The following events do **not** require reporting as an SAE/SAR, and will therefore not be subject to expedited reporting;

- Hospitalisation for disease progression.
- Disease-related deaths.
- Routine treatment or monitoring of the studied indication not associated with any deterioration in condition.
- Any admission to hospital or other institution for general care where there was no deterioration in condition.
- Hospital admissions for palliative care.
- Treatment on an emergency, outpatient basis for an event **not** fulfilling any of the definitions of serious, as provided above and not resulting in hospital admission.

An authorised medically qualified person at the trial research site should assign the causality and expectedness by referencing the reference safety information (Section 4.8 of the SmPC) and sign and date the report. If such a person is unavailable, initial reports without causality and expectedness assessment

should be submitted to the CTRU by a member of the site staff **within 24 hours**, but must be followed up by medical assessment as soon as possible thereafter.

Any changes to key information should be reported to the CTRU within 24 hours of becoming aware. Key information includes:

- Main diagnosis/symptom
- Seriousness criteria
- Outcome
- Causality and expectedness

All SAEs/SARs must be reported by emailing a completed SAE Report CRF<br>**within 24 hours** of becoming aware of the event to the CTRU<br>Email | net-02@leeds.ac.uk

On receipt of the SAE report form, the CTRU will send an acknowledgement of the SAE via email to the relevant members of the trial team at the participating site. This acknowledgement will include an SAE code which should be included on all future correspondence regarding the SAE.

The CI (or delegate) will review all SAEs including the site local investigators assessment of causality and expectedness. In the event that the CI's assessment of causality and expectedness does not match that of the local investigator, this information will be provided to the MHRA, but the event will not be downgraded.

Serious Adverse Events will be followed up until resolution.

14.2.3 Serious Adverse Reactions (SARs)

All adverse events that meet the definition of 'serious' (as defined in section 14.1) and 'related' to trial treatment and occur **between date of first trial treatment administration** and **end of trial** must be recorded in the patient notes and must be reported to the CTRU via email using SAE/SUSAR Report CRF **within 24** hours of the trial site team becoming aware of the event meeting the definition of serious.

14.2.4 Suspected Unexpected Serious Adverse Reactions (SUSARs)

A Suspected Unexpected Serious Adverse Reaction (SUSAR) is a serious adverse drug reaction which also demonstrates the following characteristic of being unexpected:
Unexpected – An adverse event, the nature **OR** severity of which is NOT consistent with the RSI in the pharmacovigilance copy of the SmPC (Section 4.8).

Suspected Unexpected Serious Adverse Reactions will be subject to expedited reporting to the Medicines and Healthcare and products Regulatory Authority (MHRA) and Research Ethics Committee (REC).

All SUSARs occurring from the **date of first trial treatment administration** must be recorded on the SUSAR Report Form and faxed to the CTRU **within 24 hours** of the trial site team becoming aware of the event (this includes participants who have withdrawn consent for data collection, see section 11.7) until end of trial.

All SAEs assigned by the local investigator, CI or CI's delegate as both suspected to be related to trial IMP, and unexpected, will be classified as SUSARs and will be subject to expedited reporting to the MHRA. The CTRU will inform the MHRA, REC and the Sponsor of SUSARs within the required expedited reporting timescales. In the event of disagreement between local assessment and the Chief Investigator (CI), local assessment will not be downgraded, but the CI may add comments prior to reporting to MHRA and REC.

Suspected Unexpected Serious Adverse Events will be followed up until resolution.

Please ensure that only one event is reported on each SAE Report CRF and SUSAR Report CRF (details of multiple symptoms should be listed if they relate to the same event). Once all resulting queries have been resolved, the CTRU will request the original CRF, and this should be posted to the CTRU and a copy retained on site.

14.3 Responsibilities

14.3.1 Principal Investigator:

- Checking for AEs and ARs when participants attend for treatment / follow-up.
- Using medical judgement in assigning seriousness and causality using the Reference Safety Information approved for the trial.
- Ensuring that all SAEs, SARs and SUSARs are recorded and reported to the CTRU within 24 hours of becoming aware of the event and provide further follow-up information as soon as available.
- Ensuring that SAEs, SARs and SUSARs are chased with CTRU, if a record of receipt is not received within 2 working days of initial reporting.
- Ensuring that AEs and ARs are recorded and reported to the CTRU in line with the requirements of the protocol.

14.3.2 Chief Investigator (CI) / delegate:

- Clinical oversight of the safety of patients participating in the trial, including an ongoing review of the risk / benefit.
- Using medical judgement in assigning seriousness, causality and expectedness of SAEs, where it has not been possible to obtain local medical assessment.
- Immediate review of all SUSARs.
- Review of specific SAEs and SARs in accordance with the trial risk assessment, and protocol, as detailed in the Trial Monitoring Plan.
- Assigning Medical Dictionary for Regulatory Activities (MedDRA) or Body System coding to all SAEs and SARs.

- Preparing the clinical sections and final sign off of the Development Safety Update Report (DSUR).

14.3.3 CTRU:

- Central data collection and verification of AEs, ARs, SAEs, SARs and SUSARs according to the trial protocol onto a MACRO database.
- Reporting safety information to the CI, delegate or independent clinical reviewer for the ongoing assessment of the risk / benefit according to the Trial Monitoring Plan.
- Reporting safety information to the independent oversight committee identified for the trial (Data Monitoring & Ethics Committee (DMEC) and Trial Steering Committee (TSC)) according to the Trial Monitoring Plan.
- Expedited reporting of SUSARs to the MHRA, REC and Sponsor within required timelines.
- Notifying Investigators of SUSARs that occur within the trial.
- Checking for (annually) and notifying Principal Investigators of updates to the Reference Safety Information for the trial.
- Preparing standard tables and other relevant information for the DSUR in collaboration with the CI and ensuring timely submission to the MHRA and REC.
- Notify Servier of any SUSARs occurring in trial participants using the CTRU SUSAR CRF. Initials and date of birth will be removed from the CRF before sending to Servier. Participants will be identified by trial number only.

14.3.4 Trial Steering Committee (TSC):

In accordance with the Trial Terms of Reference for the TSC, periodically reviewing safety data and liaising with the DMEC regarding safety issues.

14.3.5 Data Monitoring & Ethics Committee (DMEC):

In accordance with the Trial Terms of Reference for the DMEC, periodically reviewing unblinded overall safety data to determine patterns and trends of events, or to identify safety issues, which would not be apparent on an individual basis.

15. Participant Questionnaires

Quality of life will be assessed by participants' self-reported symptoms, and functioning, using validated instruments completed by participants at the point of randomisation and then at 6-weekly intervals and on disease progression. All instruments are self-administered to avoid interviewer bias.

The European Organisation for Research and Treatment of Cancer (EORTC) QLQ-C30 is a well-validated questionnaire developed to assess the quality of life of cancer patients by scoring patients on scales that assess global health status, social, physical and emotional functioning and common symptoms. Various disease-specific scales have been developed to work in combination with the QLQ-C30 and the GINET-21 is specific to neuroendocrine tumours. In combination with the QLQ-C30, the GINET-21 questionnaire

provides information on neuroendocrine symptoms including diarrhoea and flushing, treatment side-effects and disease-related worries[50, 51]. This information relates to the previous 1-week time period.

15. Samples for Research

Blood samples (collected pre-dose, at 6 weeks and at progression) and tissue including patient-specific archival tumour tissue will be collected and stored for potential future exploratory research into factors that may influence the development of agents to treat this diagnosis and/or response to treatment (where response is defined broadly to include efficacy, tolerability or safety). This may include the analysis of tumour-specific and circulating biomarkers, such as circulating tumour cells, circulating tumour DNA, mRNA, proteins or metabolites. Blood samples may also be used for the development of mouse models of NEC. In the event that additional tumour molecular profiling is required to understand further any response to therapy used, a sample of the most recent tumour biopsy for additional research may be requested.

Molecular imaging in a defined number of patients may also be considered. There will be measurement of neuron-specific enolase as a potential biomarker of response to treatment.

16. Endpoints

16.1 Primary endpoint

6 month progression-free survival rate is defined as a binary outcome (progression-free or not) within the timeframe of treatment start date until 6 months after randomisation.

Note that for the purposes of the trial, disease progression will usually be defined radiologically (RECIST v1.1). The date of progression will be taken as the date of the scan which concluded progressive disease. However, it is acknowledged that there will be circumstances where disease progression is determined clinically due to a global deterioration in clinical status attributable to disease progression in the view of the local investigator. If possible, an appropriate radiological assessment should be performed to document the disease status as per RECIST v1.1. In the rare circumstances that disease progression is determined clinically and it is not appropriate to confirm it radiologically, the date of progression is defined as the date of documented clinical disease progression.

16.2 Secondary endpoints

- Progression-free survival is defined as the time from randomisation to progression or death from any cause. Individuals will be censored if they are lost to follow-up or are still alive and progression-free at the time of analysis.
- Overall survival is defined as the time from randomisation to death from any cause. Individuals will be censored if they are lost to follow-up or still alive at the time of the analysis.
- Objective response rate is defined using RECIST v1.11
- Toxicity is defined as the AE and SAEs reported on the trial according to CTCAE v5.0.
- Quality of life will be assessed according to the patient reported outcome measures; EORTC QLQ-C30 and EORTC QLQ-GI.NET21.

- Concentration of neuron-specific enolase

16.3 Samples for research

- Circulating tumour cells and DNA; Blood samples will be analysed centrally within specific timeframes for the purpose of quantification and molecular characterisation (through Next Generation Sequencing) of circulating tumour cells and circulating tumour DNA.
- Tumour-specific and circulating biomarkers e.g. tumour DNA, messenger ribonucleic acid, proteins or metabolites will be investigated to look for factors which may aid future diagnosis or response to treatment.
- Patient-derived or cell line-derived NEC xenografts may be used to test the activity of novel drug combinations.

17. Statistical Considerations

17.1 Sample size calculation

With 80% power and significance level of 5% (one-sided), a maximum of 48 patients per arm are required to establish the efficacy of each treatment. To allow for a drop-out rate of 5%, a maximum of 102 patients are required to be randomised to receive either docetaxel or nal-IRI (in combination with 5-FU/folinic acid).

17.2 Efficacy

The primary efficacy objective of the trial is to determine the 6-month progression-free survival (PFS) rate in each treatment arm. The trial is designed to have an 80% chance of demonstrating that the one-sided 95% confidence interval of the 6 month PFS rate excludes 15%, if the true rate is at least 30%, where 30% is the required level of efficacy and a rate of less than 15% would give grounds for rejection, i.e. the relevant treatment would be considered not to have not reached an acceptable level of efficacy to warrant further evaluation. A rate of 30% is considered achievable and of clinical benefit. This rate would indicate further investigation of the corresponding treatment. The proportions of 15% and 30% were chosen by the TMG in line with existing literature and research. Of those who reported the proportion progression-free at 6 months, the lowest was approximately 15%[20] and the highest approximately 25%[17]. Therefore, for either of the treatments being considered to be taken forward for further research, they should provide estimates that are at least as good as the lower value and aim to improve on the higher value.

The design is an adaptation of a one-stage treatment design proposed by Simon, Wittes and Ellenberg[50], where the A'Hern design is first implemented to assess efficacy of each treatment separately to ensure a pre-specified minimum level of activity prior to selection. Selection criteria are then applied following the design of Simon, Wittes and Ellenberg, should both treatments be deemed sufficiently efficacious, to establish which treatment to take forward into a phase III trial. The A'Hern method is advantageous over other single-stage designs, since it uses the exact binomial distribution as opposed to a normal approximation to the binomial distribution which can give a substantial margin of error in small trial sizes.

Additionally, decision criteria are specified which, if reached, could enable earlier planning of a subsequent phase III trial.

With 80% power and a significance level of 5% (one-sided), a maximum of 48 patients per arm are required to establish the efficacy of each treatment independently. To allow for a drop-out rate of 5%, a maximum of 102 patients are required to be randomised to receive either docetaxel or nal-IRI (in combination with 5-FU). The intention of the trial is to show that the regimens are sufficiently active in this population of patients, but not to show that one regimen is superior. A treatment arm may be considered for further evaluation using the treatment selection approach described below, if at least 12 out of 48 evaluable patients are progression free at 6 months (equating to a success rate of 25% which would have a lower one-sided 95% confidence limit of 15.1%).

17.3 Treatment Selection

If both treatments successfully exceed the pre-defined criteria and have lower one-sided 95% confidence limits greater than or equal to 15.1%, Simon's design proposes that the treatment with the higher PFS rate at 6 months should be selected, regardless how small its advantage over the other treatment appears. The selection theory approach ensures that the better treatment will be selected with a high probability P. For instance, hypothesising a 25% PFS rate at 6 months in the docetaxel group, and a 30% PFS rate in the nal-IRI group, with 48 patients per arm, the study would correctly select nal-IRI with 69% probability (assuming that in 50% of ambiguous cases, the correct treatment would be selected), based on PFS alone. If, however, the difference in PFS rates at 6 months is 10% (i.e. 35% of patients progression-free at 6 months in the nal-IRI group), the study would correctly select nal-IRI with 85% probability, based on PFS alone. Therefore, if the difference in 6-month PFS rates is less than 5%, alternative selection criteria such as toxicity rates or quality of life may be considered in addition to PFS rate. If only one of the treatments successfully passes the pre-defined criteria, this treatment will be selected for further investigation.

17.4 The recruitment rate

The UK has an established network of investigators for neuroendocrine studies under the auspices of the National Cancer Research Network (NCRN), who have been actively engaged in the design of this study; this establishes a group of people who are likely to be co-investigators at the various recruiting hospitals. This allows an accurate estimate of recruitment rates.

The trial aims to recruit 102 participants within a 37 month recruitment period across 16 sites. Recruitment rates are based on 1.5 patients per month for 6 months (as centres are opened) and 3 patients per month for the remaining 31 months.

18. Statistical Analysis

18.1 General considerations

Statistical analysis is the responsibility of the CTRU Trial Statistician under the supervision of the Supervising Statistician. A full statistical analysis plan (SAP) will be written before any analysis is undertaken. The analysis plan will be written in accordance with current CTRU standard operating

procedures and will be finalised and agreed by the trial and supervising statisticians, the Chief Investigator and the CTRU Delivery and Scientific Leads, prior to any analysis taking place. Any changes to the finalised analysis plan, and reasons for changes, will be documented.

The number of patients registered/randomised to the study who do not go on to receive any study treatment will be monitored. There will be an assessment of the number of patients who are randomised, but do not start treatment within 28 days of their baseline scan.

18.2 Frequency of analyses

A DMEC will be set up to independently review data on safety, protocol adherence and recruitment. Interim reports will be presented to the DMEC in strict confidence at, at least, yearly intervals. This committee, in light of the interim data, and of any advice or evidence they wish to request, will advise the Trial Steering Committee if there is proof beyond reasonable doubt that for example one treatment is better or if there are concerns regarding the safety of the trial. No formal interim analyses are planned, hence no statistical testing will take place until final analysis.

Final analysis will take place after all those randomised have reached the primary endpoint.

18.3 Primary endpoint analysis

The primary analysis population will be defined as those who have received at least one dose of either docetaxel or nal-IRI.
Treatment efficacy will be assessed as defined in Section 17.2. To evaluate the efficacy of either treatment, the proportion of those progression-free at 6 months will be estimated with a one-sided 95% confidence interval using exact methods.

18.4 Secondary endpoint analysis

Unless stated otherwise within the SAP, the analysis population will be the same as the primary analysis population.

For each treatment group, the analysis of the time to event outcomes, progression-free and overall survival, will be descriptive. The proportion of patients who have not experienced an event at 3 monthly intervals will be presented with Kaplan-Meier estimates of the survival functions.

For each treatment group, the number and proportion of patients in each response group (complete response, partial response, stable disease and progressive disease etc.) at 6 months post randomisation will be summarised along with 95% confidence intervals.

Toxicities will be summarised within each treatment group using the categories associated with CTCAE V5.0.

Quality of Life will be summarised using the mean subscale scores of each questionnaire at each time point along with 95% confidence intervals. These summaries will be adjusted for the baseline values of each subscale.

Neuron-specific enolase will be analysed to assess whether it is associated with response to treatment via an ordinal/binary logistic regression model adjusting for the stratification factors of the trial along with any appropriate interaction variables.

The analysis of the research samples described in section 16.3 will be fully documented in the SAP.

19. Trial Monitoring

A Trial Monitoring Plan will be developed and agreed by the Trial Management Group (TMG) and Trial Steering Committee (TSC) based on the trial risk assessment; this may include on site monitoring.

19.1 Data Monitoring and Ethics Committee (DMEC)

The independent DMEC will review the safety and ethics of the study. Detailed unblinded reports will be prepared by the CTRU for the DMEC at approximately 6 monthly intervals. The DMEC will be provided with detailed unblinded reports containing the information agreed in the data monitoring analysis plan.

19.2 Data monitoring

Data will be monitored for quality and completeness by the CTRU. Missing data will be chased until it is received, confirmed as not available or the trial is at analysis. However, missing data items will not be chased from participants (although missing questionnaires sometimes are). The CTRU will reserve the right to intermittently conduct source data verification exercises on a sample of participants, which will be carried out by staff from the CTRU. Source data verification will involve direct access to patient notes at the participating hospital sites and the ongoing central collection of copies of consent forms and other relevant investigation reports.

On-site monitoring will be centred on a risk-based strategy and a thorough risk assessment will be completed by the CTRU as part of the trial set-up process to ascertain the frequency and intensity of monitoring visits required (although additional monitoring may be conducted if necessary). This risk assessment and associated monitoring plan will be stored at the CTRU.

19.3 Clinical governance issues

To ensure responsibility and accountability for the overall quality of care received by participants during the study period, clinical governance issues pertaining to all aspects of routine management will be brought to the attention of the TSC and, where applicable, to individual NHS Trusts.

20. Quality Assurance and ethical considerations

20.1 Quality Assurance

The trial will be conducted in accordance with the principles of Good Clinical Practice (GCP) in clinical trials, as applicable under UK regulations, the NHS Research Governance Framework (RGF) and Scottish

Executive Health Department Research Governance Framework for Health and Social Care 2006, and through adherence to CTRU Standard Operating Procedures (SOPs).

20.2 Serious breaches

CTRU and Sponsor have systems in place to ensure that serious breaches of GCP or the trial protocol are picked up and reported. Investigators are required to promptly notify the CTRU of a serious breach (as defined in Regulation 29A of the Medicines for Human Use (Clinical Trials) Regulations 2004 and amendments) that they become aware of. A 'serious breach' is a breach which is likely to effect to a significant degree –

(a) the safety or physical or mental integrity of the subjects of the trial; or
(b) the scientific value of the trial.

In the event of doubt or for further information, the Investigator should contact the CTRU.

20.3 Ethical and regulatory considerations

The trial will be performed in accordance with the recommendations guiding physicians in biomedical research involving human subjects adopted by the 18th World Medical Assembly, Helsinki, Finland, 1964, amended at the 52nd World Medical Association General Assembly, Edinburgh, Scotland, 1996. Informed written consent will be obtained from the patients prior to registration into the study. The right of a participant to refuse participation without giving reasons must be respected. The participant must remain free to withdraw at any time from the study without giving reasons and without prejudicing his/her further treatment. The study will be submitted to and approved by a main REC and the appropriate Site Specific Assessor for each participating centre prior to entering patients into the study. The CTRU will provide the main REC with a copy of the final protocol, patient information sheets, consent forms and all other relevant study documentation.

21. Confidentiality

All information collected during the course of the trial will be kept strictly confidential. Information will be held securely on paper and electronically at the CTRU. The CTRU will comply with all aspects of the 2018 General Data Protection Regulation and operationally this will include:

- consent from participants to record personal details including name, date of birth and hospital ID
- appropriate storage, restricted access and disposal arrangements for participant personal and clinical details
- consent from participants for access to their medical records by responsible individuals from the research staff or from regulatory authorities, where it is relevant to trial participation
- consent from participants for the data collected for the trial to be used to evaluate safety and develop new research.
- data collection forms that are transferred to or from the CTRU will be coded with a trial number and will include two participant identifiers, usually the participant's initials and date of birth.

- where central monitoring of source documents by CTRU (or copies of source documents) is required (such as scans or local blood results), the participant's name must be obliterated by site before sending.
- where anonymisation of documentation is required, sites are responsible for ensuring only the instructed identifiers are present before sending to CTRU.

If a participant withdraws consent from further trial treatment or from further collection of data, their data and samples will remain on file and will be included in the final trial analysis.

The trial staff at the participating sites will be responsible for ensuring that any data / documentation sent to the CTRU is appropriately anonymised as per instructions given by CTRU in accordance with the trial procedures to conform with the 2018 General Data Protection Regulation.

Published results will not contain any personal data that could allow identification of individual patients.

21.1 Trial record retention and archiving

Essential documents will be maintained at the CTRU and at the Investigator Sites in a way that will facilitate the management of the trial, audit and inspection. Documents should be securely stored and access restricted to authorised personnel. At the end of the trial, data will be securely archived in line with the Sponsor's procedures for a minimum of 15 years. Data held by the CTRU will be archived in the Sponsor archive facility and site data and documents will be archived at the participating sites. Following authorisation from the Sponsor, arrangements for confidential destruction will then be made.

22. Sponsorship and indemnity

The Christie NHS Foundation Trust will act as the sponsor for this study. Delegated responsibilities will be assigned to the CTRU to manage the trial on behalf of the sponsor and to the participating sites recruiting patients into this trial.

As the sponsor is an NHS organisation, the NHS indemnity scheme will apply.

Participating sites will be liable for clinical negligence and other negligent harm to participants taking part in the study and covered by the duty of care owed to them by the sites concerned. For participating sites which are part of the NHS, the NHS indemnity scheme will also apply.

The manufacturer supplying IMP has accepted limited liability related to the manufacturing and original packaging of the study drug and to the losses, damages, claims or liabilities incurred by study participants based on known or unknown Adverse Events which arise out of the manufacturing and original packaging of the study drug, but not where there is any modification to the study drug (including without limitation re-packaging and blinding).

23. Study Organisational Structure

23.1 Individuals and Individual Organisations

Chief Investigator (CI) – The CI is involved in the design, conduct, co-ordination and management of the trial. The CI will have overall responsibility for the design and set-up of the trial, the investigational drug supply and pharmacovigilance within the trial.

Trial Sponsor – The Sponsor is responsible for trial initiation management and financing of the trial as defined by Directive 2001/20/EC. These responsibilities are delegated to the CTRU as detailed in the trial contract.

Clinical Trials Research Unit – The CTRU will have responsibility for conduct of the trial as delegated by the Sponsor in accordance with relevant GCP standards and CTRU SOPs. The CTRU will provide set-up and monitoring of trial conduct to CTRU SOPs, and the GCP Conditions and Principles as detailed in the UK Medicines for Human Use (Clinical Trials) Regulations 2006 including, randomisation design and service, database development and provision, protocol development, CRF design, trial design, source data verification, monitoring schedule and statistical analysis for the trial. In addition the CTRU will support main REC, Site Specific Assessment and NHS Permissions submissions and clinical set-up, ongoing management including training, monitoring reports and promotion of the trial. The CTRU will be responsible for the day-to-day running of the trial including trial administration, database administrative functions, data management, safety reporting and all statistical analyses.

Central pharmacy – Clinigen Clinical Trial Services is responsible for the labelling, packaging and distribution of trial IMP and management of the IRT system.

Central laboratory for translational research - CRUK Manchester Institute (or designated substitute) is responsible for the analysis of the translational blood samples and archival paraffin-embedded tissue.

23.2 Oversight and Trial Monitoring Groups

Trial Management Group (TMG) – The TMG, comprising the CI, CTRU team, other key external members of staff involved in the trial and a nursing representative will be assigned responsibility for the clinical set-up, on-going management, promotion of the trial, and for the interpretation and publishing of the results. Specifically the TMG will be responsible for (i) protocol completion, (ii) CRF development, (iii) obtaining approval from the main REC and supporting applications for Site Specific Assessments, (iv) submitting a CTA, (v) completing cost estimates and project initiation, (vi) nominating members and facilitating the TSC and DMEC, (vii) reporting of serious adverse events, (viii) monitoring of screening, recruitment, treatment and follow-up procedures, (ix) auditing consent procedures, data collection, trial end-point validation and database development.

Trial Steering Committee (TSC) – The TSC will provide overall supervision of the trial, in particular trial progress, adherence to protocol, participant safety and consideration of new information. It will include an Independent Chair, not less than two other independent members and a consumer representative. The CI and other members of the TMG may attend the TSC meetings and present and report progress. The Committee will meet annually as a minimum.

Data Monitoring and Ethics Committee (DMEC) – The DMEC will include independent membership and will review the safety and ethics of the trial by reviewing interim data during recruitment and follow-up and will report to the TSC. The Committee will meet annually as a minimum.

24. Publication Policy

The trial will be registered with an authorised registry, according to the International Committee of Medical Journal Editors (ICMJE) Guidelines, prior to the start of recruitment.

The main trial results will be published in the name of the trial in a peer-reviewed journal, on behalf of all collaborators. The manuscript will be prepared by a writing group, appointed from amongst the Trial Management Group, and high accruing clinicians. All participating centres and clinicians will be acknowledged in this publication, together with staff from the CTRU.

The success of the trial depends upon the collaboration of all participants. For this reason, credit for the main results will be given to all those who have collaborated in the trial, through authorship and contributorship. Uniform requirements for authorship for manuscripts submitted to medical journals will guide authorship decisions. These state that authorship credit should be based only on substantial contribution to:

- conception and design, or acquisition of data, or analysis and interpretation of data,
- drafting the article or revising it critically for important intellectual content,
- and final approval of the version to be published,
- and that all these conditions must be met (www.icmje.org).

In light of this, the Chief Investigator, and relevant senior CTRU staff will be named as authors in any publication. In addition, all collaborators will be listed as contributors for the main trial publication, giving details of roles in planning, conducting and reporting the trial. The manuscript will be prepared by a writing group, appointed from amongst the Trial Management Group, and high accruing clinicians. All participating centres and clinicians will be acknowledged in this publication together with staff from the CTRU.

To maintain the scientific integrity of the trial, data will not be released prior to the first publication of the analysis of the primary endpoint, either for trial publication or oral presentation purposes, without the permission of the Trial Steering Committee and Sponsor. In addition, individual collaborators must not publish data concerning their participants which is directly relevant to the questions posed in the trial until the first publication of the analysis of the primary endpoint. No investigator may present or attempt to publish data relating to this trial without prior permission from the TMG and sponsor.

Authorship of any secondary publications e.g. relating to the various biological studies will reflect the intellectual and time input into these studies, and will not be the same as on the primary publication.

25. References

1. Eisenhauer EA, Therasse P, Bogaerts J, et al. New response evaluation criteria in solid tumours: Revised RECIST guideline (version 1.1). *European Journal of Cancer* 2009;45(2):228-47. doi: 10.1016/j.ejca.2008.10.026
2. Taal BG, Visser O. Epidemiology of Neuroendocrine Tumours. *Neuroendocrinology* 2004;80(1):3-7. doi: 10.1159/000080731
3. Korse CM, Taal BG, van Velthuysen M-LF, et al. Incidence and survival of neuroendocrine tumours in the Netherlands according to histological grade: Experience of two decades of cancer registry. *European Journal of Cancer* 2013;49(8):1975-83. doi: 10.1016/j.ejca.2012.12.022
4. Bettini R, Boninsegna L, Mantovani W, et al. Prognostic factors at diagnosis and value of WHO classification in a mono-institutional series of 180 non-functioning pancreatic endocrine tumours. *Annals of Oncology* 2008;19(5):903-08. doi: 10.1093/annonc/mdm552
5. Ahlman H, Nilsson O, McNicol AM, et al. Poorly-Differentiated Endocrine Carcinomas of Midgut and Hindgut Origin. *Neuroendocrinology* 2007;87(1):40-46. doi: 10.1159/000109976
6. Brenner B, Tang LH, Shia J, et al. Small Cell Carcinomas of the Gastrointestinal Tract: Clinicopathological Features and Treatment Approach. *Seminars in Oncology* 2007;34(1):43-50. doi: 10.1053/j.seminoncol.2006.10.022
7. Rindi G. KDS, Arnold R. WHO classification of Tumours of the Digestive System, 4th edition. *WHO Press* 2010
8. WHO Classification of Tumours of Endocrine Organs2017.
9. Nagtegaal ID OR, Klimstra D, Paradis V, Rugge M, Shirmacher P, Washington KM, Carneiro F, Cree IA. The 2019 WHO classification of tumours of the digestive system. *Histopathology* 2020;76:182-88.
10. Brenner B, Tang LH, Klimstra DS, et al. Small-Cell Carcinomas of the Gastrointestinal Tract: A Review. *Journal of Clinical Oncology* 2004;22(13):2730-39. doi: 10.1200/jco.2004.09.075
11. Galanis E, Frytak S, Lloyd RV. Extrapulmonary small cell carcinoma. *Cancer* 1997;79(9):1729-36. doi: 10.1002/(sici)1097-0142(19970501)79:9<1729::aid-cncr14>3.0.co;2-#
12. Lee S.S. LJL, Ryu M.H., Chang H.M., Kim T.W., Kim W.K., Lee J.S., Jang S.J., Khang S.K., Kang Y.K. Extrapulmonary small cell carcinoma: single center experience with 61 patients. *Acta Oncologica* 2007;46(6):846-51.
13. Bosman F.T. CF, Hruban R.H., Theise N.D. WHO classification of tumours of the digestive system, 4th edition. *WHO Press* 2010
14. Shia J, Tang LH, Weiser MR, et al. Is Nonsmall Cell Type High-grade Neuroendocrine Carcinoma of the Tubular Gastrointestinal Tract a Distinct Disease Entity? *The American Journal of Surgical Pathology* 2008;32(5):719-31. doi: 10.1097/pas.0b013e318159371c
15. Moertel CG, Kvols LK, O'Connell MJ, et al. Treatment of neuroendocrine carcinomas with combined etoposide and cisplatin. Evidence of major therapeutic activity in the anaplastic variants of these neoplasms. *Cancer* 1991;68(2):227-32. doi: 10.1002/1097-0142(19910715)68:2<227::aid-cncr2820680202>3.0.co;2-i
16. Sorbye H, Welin S, Langer SW, et al. Predictive and prognostic factors for treatment and survival in 305 patients with advanced gastrointestinal neuroendocrine carcinoma (WHO G3): The NORDIC NEC study. *Annals of Oncology* 2012;24(1):152-60. doi: 10.1093/annonc/mds276
17. Hentic O, Hammel P, Couvelard A, et al. FOLFIRI regimen: an effective second-line chemotherapy after failure of etoposide-platinum combination in patients with neuroendocrine carcinomas grade 3. *Endocrine Related Cancer* 2012;19(6):751-57. doi: 10.1530/erc-12-0002
18. Hadoux J. PD, Guigay J., et al. Oxaliplatin-based chemotherapy for grade 3 neuroendocrine carcinoma after failure of platinum-based chemotherapy. *European Neuro Endocrine Tumour Society* 2013;Abstract J2
19. Welin S, Sorbye H, Sebjornsen S, et al. Clinical effect of temozolomide-based chemotherapy in poorly differentiated endocrine carcinoma after progression on first-line chemotherapy. *Cancer* 2011;117(20):4617-22. doi: 10.1002/cncr.26124
20. Olsen IH, Knigge U, Federspiel B, et al. Topotecan Monotherapy in Heavily Pretreated Patients with Progressive Advanced Stage Neuroendocrine Carcinomas. *Journal of Cancer* 2014;5(8):628-32. doi: 10.7150/jca.9409

21. Heetfeld M, Chougnet CN, Olsen IH, et al. Characteristics and treatment of patients with G3 gastroenteropancreatic neuroendocrine neoplasms. *Endocrine-Related Cancer* 2015;22(4):657-64. doi: 10.1530/erc-15-0119
22. Olsen IH, Sørensen JB, Federspiel B, et al. Temozolomide as Second or Third Line Treatment of Patients with Neuroendocrine Carcinomas. *The Scientific World Journal* 2012;2012:1-4. doi: 10.1100/2012/170496
23. Chen L-T, Von Hoff DD, Li C-P, et al. Expanded analyses of napoli-1: Phase 3 study of MM-398 (nal-IRI), with or without 5-fluorouracil and leucovorin, versus 5-fluorouracil and leucovorin, in metastatic pancreatic cancer (mPAC) previously treated with gemcitabine-based therapy. *Journal of Clinical Oncology* 2015;33(3_suppl):234-34. doi: 10.1200/jco.2015.33.3_suppl.234
24. Smyth JF, Smith IE, Sessa C, et al. Activity of docetaxel (Taxotere) in small cell lung cancer. *European Journal of Cancer* 1994;30(8):1058-60. doi: 10.1016/0959-8049(94)90455-3
25. F.V. F. Docetaxel for previously treated non-small-cell lung cancer. *Oncology (Williston Park)* 2002;16(6 Suppl 6):45-51.
26. Innocenti F, Kroetz DL, Schuetz E, et al. Comprehensive Pharmacogenetic Analysis of Irinotecan Neutropenia and Pharmacokinetics. *Journal of Clinical Oncology* 2009;27(16):2604-14. doi: 10.1200/jco.2008.20.6300
27. Bellanti F, Kågedal B, Della Pasqua O. Do pharmacokinetic polymorphisms explain treatment failure in high-risk patients with neuroblastoma? *European Journal of Clinical Pharmacology* 2011;67(S1):87-107. doi: 10.1007/s00228-010-0966-3
28. Mathijssen R.H. vARJ, Verweii J., Loos W.J., Nooter K., Stoter G., Sparreboom A. Clinical pharmacokinetics and metabolism of irinotecan (CPT-11). *Clinical Cancer Research* 2001;7(8):2182-94.
29. Kawato Y. AM, Hirota Y., Kuga H., Sato K. Intracellular roles of SN-38, a metabolite of the camptothecin derivative CPT-11, in the antitumor effect of CPT-11. *Cancer Research* 1991;51(16):4187-91.
30. Garcia-Carbonero R. SJG. Current perspectives on the clinical experience, pharmacology, and continued development of the camptothecins. *Clinical Cancer Research* 2002;8(3):641-61.
31. Vanhoefer U, Harstrick A, Achterrath W, et al. Irinotecan in the Treatment of Colorectal Cancer: Clinical Overview. *Journal of Clinical Oncology* 2001;19(5):1501-18. doi: 10.1200/jco.2001.19.5.1501
32. Drummond DC, Noble CO, Guo Z, et al. Development of a Highly Active Nanoliposomal Irinotecan Using a Novel Intraliposomal Stabilization Strategy. *Cancer Research* 2006;66(6):3271-77. doi: 10.1158/0008-5472.can-05-4007
33. Kang MH, Wang J, Makena MR, et al. Activity of MM-398, Nanoliposomal Irinotecan (nal-IRI), in Ewing's Family Tumor Xenografts Is Associated with High Exposure of Tumor to Drug and High SLFN11 Expression. *Clinical Cancer Research* 2015;21(5):1139-50. doi: 10.1158/1078-0432.ccr-14-1882
34. Kalra AV, Kim J, Klinz SG, et al. Preclinical Activity of Nanoliposomal Irinotecan Is Governed by Tumor Deposition and Intratumor Prodrug Conversion. *Cancer Research* 2014;74(23):7003-13. doi: 10.1158/0008-5472.can-14-0572
35. Tsai C.S. PJW, Chen L.T. Nanovector-based therapies in advanced pancreatic cancer. *J Gastrointest Oncol* 2011;2(3):185-94.
36. Koshkaryev A, Fitzgerald J, Kim J, et al. Abstract 2075: Differential tissue clearance results in improved therapeutic index for irinotecan liposome injection (ONIVYDE) when combined with the PARP inhibitor veliparib in preclinical cervical tumors. *Cancer Research* 2016;76(14 Supplement):2075-75. doi: 10.1158/1538-7445.am2016-2075
37. Wang-Gillam A, Li C-P, Bodoky G, et al. Nanoliposomal irinotecan with fluorouracil and folinic acid in metastatic pancreatic cancer after previous gemcitabine-based therapy (NAPOLI-1): a global, randomised, open-label, phase 3 trial. *The Lancet* 2016;387(10018):545-57. doi: 10.1016/s0140-6736(15)00986-1
38. Camptosar (irinotecan) U.S. Package Insert. http://wwwaccessdatafdagov/drugsatfda_docs/label/2014/020571s048lblpdf Accessed 23 March 2016
39. Irinotecan hydrochloride Summary of Product Characteristics. https://wwwmedicinesorguk/emc/medicine/21586 Accessed 23 March 2016
40. Baker JHE, Lam J, Kyle AH, et al. Irinophore C, a Novel Nanoformulation of Irinotecan, Alters Tumor Vascular Function and Enhances the Distribution of 5-Fluorouracil and Doxorubicin. *Clinical Cancer Research* 2008;14(22):7260-71. doi: 10.1158/1078-0432.ccr-08-0736

41. Verreault M, Strutt D, Masin D, et al. Vascular normalization in orthotopic glioblastoma following intravenous treatment with lipid-based nanoparticulate formulations of irinotecan (Irinophore C™), doxorubicin (Caelyx®) or vincristine. *BMC Cancer* 2011;11(1) doi: 10.1186/1471-2407-11-124
42. Tournigand C, André T, Achille E, et al. FOLFIRI Followed by FOLFOX6 or the Reverse Sequence in Advanced Colorectal Cancer: A Randomized GERCOR Study. *Journal of Clinical Oncology* 2004;22(2):229-37. doi: 10.1200/jco.2004.05.113
43. Fuchs CS, Marshall J, Mitchell E, et al. Randomized, Controlled Trial of Irinotecan Plus Infusional, Bolus, or Oral Fluoropyrimidines in First-Line Treatment of Metastatic Colorectal Cancer: Results From the BICC-C Study. *Journal of Clinical Oncology* 2007;25(30):4779-86. doi: 10.1200/jco.2007.11.3357
44. Mullany S, Svingen PA, Kaufmann SH, et al. Effect of adding the topoisomerase I poison 7-ethyl-10-hydroxycamptothecin (SN-38) to 5-fluorouracil and folinic acid in HCT-8 cells: elevated dTTP pools and enhanced cytotoxicity. *Cancer Chemotherapy and Pharmacology* 1998;42(5):391-99. doi: 10.1007/s002800050835
45. Guichard S, Cussac D, Hennebelle I, et al. Sequence-dependent activity of the irinotecan-5FU combination in human colon-cancer model HT-29in vitro andin vivo. *International Journal of Cancer* 1997;73(5):729-34. doi: 10.1002/(sici)1097-0215(19971127)73:5<729::aid-ijc20>3.0.co;2-#
46. Taieb J, Lecomte T, Aparicio T, et al. FOLFIRI.3, a new regimen combining 5-fluorouracil, folinic acid and irinotecan, for advanced pancreatic cancer: results of an Association des Gastro-Enterologues Oncologues (Gastroenterologist Oncologist Association) multicenter phase II study. *Annals of Oncology* 2006;18(3):498-503. doi: 10.1093/annonc/mdl427
47. Yoo C, Hwang JY, Kim JE, et al. A randomised phase II study of modified FOLFIRI.3 vs modified FOLFOX as second-line therapy in patients with gemcitabine-refractory advanced pancreatic cancer. *British Journal of Cancer* 2009;101(10):1658-63. doi: 10.1038/sj.bjc.6605374
48. Gebbia V, Maiello E, Giuliani F, et al. Irinotecan Plus Bolus/Infusional 5-Fluorouracil and Leucovorin in Patients With Pretreated Advanced Pancreatic Carcinoma. *American Journal of Clinical Oncology* 2010;33(5):461-64. doi: 10.1097/coc.0b013e3181b4e3b0
49. Neuzillet C, Hentic O, Rousseau B, et al. FOLFIRI regimen as second-/third-line chemotherapy in patients with advanced pancreatic adenocarcinoma refractory to gemcitabine and platinum salts: A retrospective series of 70 patients. *Journal of Clinical Oncology* 2011;29(4_suppl):272-72. doi: 10.1200/jco.2011.29.4_suppl.272
50. Simon R. WRE, Ellenberg S.S. Randomized phase II clinical trials. *Cancer Treat Rep* 1985;69(12):1375-81.

26. Appendices

Appendix 1 - Response Evaluation Criteria In Solid Tumours (RECIST)

Response to treatment will be assessed based on RECIST v1.1. A copy of the revised RECIST may be obtained at:

http://www.eortc.be/recist/

Published date: January 2009.

Appendix 2 - Eastern cooperative oncology group (ECOG) performance status (PS)

ECOG PS	Description
0	Able to carry out all normal activity without restriction
1	Restricted in physically strenuous activity but ambulatory and able to carry out light work
2	Ambulatory and capable of all self-care but unable to carry out any work: up and about more than 50% of waking hours
3	Capable only of limited self-care; confined to bed or chair more than 50% of waking hours
4	Completely disabled; cannot carry out any self-care; totally confined to bed or chair

Appendix 3 - Cockcroft-Gault Equation

Cockcroft-Gault for creatinine clearance estimation

$$\text{Male} = \frac{1.23 \times (140\text{-age}) \times \text{weight (kg)}}{\text{Serum creatinine } (\mu\text{mol/l})}$$

$$\text{Female} = \frac{1.04 \times (140\text{-age}) \times \text{weight (kg)}}{\text{Serum creatinine } (\mu\text{mol/l})}$$

If the calculated creatinine clearance is less than 30 ml/min at baseline, GFR may be assessed using either Cr51-EDTA or 99mTc-DTPA clearance method to confirm if GFR is ≥ 30 ml/min. If it is not possible to complete Cr51-EDTA or 99mTc-DTPA clearance method or where this confirms baseline GFR is < 30 the patient will be determined ineligible.

The Wright formula is considered an acceptable alternative to Cockroft-Gault, for sites where use of the Wright formula is usual local practice.

Appendix 4 – Management of nal-IRI/5-FU/folinic acid toxicities (as per local standard practice)

Suggested management could include:

Antiemetics
Dexamethasone and a 5-HT3 blocker (eg ondansetron or granisetron) should be administered to all patients as pre-medications unless contraindicated for the individual patient. Anti-emetics should also be prescribed as clinically indicated during the trial period.

Colony stimulating factors
Use of granulocyte colony-stimulating factors is permitted to treat patients with neutropenia or neutropenic fever as per American Society of Oncology guidelines; prophylactic use of GCSF can be considered in those patients who have had at least one episode of grade 3 or 4 neutropenia or neutropenic fever while receiving therapy or have had documented grade 3 or 4 neutropenia or neutropenic fever while receiving prior anti-neoplastic therapy.

Therapy for diarrhoea
Diarrhoea can occur early (onset in less than 24 hours after starting nal-IRI) or late (more than 24 hours). Early onset diarrhoea may be accompanied by cholinergic symptoms: sweating abdominal cramping, myosis and salivation. Patients should be made aware of the risk of delayed diarrhoea which can be debilitating and, on rare occasions, life-threatening since persistent loose or watery stools can result in dehydration, electrolyte imbalance, colitis, GI ulceration, infection or sepsis.
As soon as the first liquid stool occurs, the patient should start drinking large volumes of beverages containing electrolytes and an appropriate anti-diarrhoeal therapy should be initiated immediately.
Prophylactic or therapeutic treatment with atropine in patients experiencing early onset diarrhoea with cholinergic symptoms (0.25 mg to 1 mg, administered intravenously or subcutaneously), should be considered unless contraindicated.

Chemotherapy-induced diarrhoea should be treated as per local standard practice.

Suggested management could include (but is not limited to) the following: Patients should have loperamide (or equivalent) readily available to begin treatment for late diarrhoea. Loperamide should be initiated at first occurrence of poorly formed or loose stools or at the earliest onset of bowel movements becoming more frequent than normal. Loperamide should be given until patient is without diarrhoea for at least 12 hours. Loperamide should not be used for more than 48 consecutive hours due to risk of paralytic ileus. If diarrhoea persists for more than 48 hours, stop loperamide, monitor and replace fluid electrolytes. Nal-IRI treatment should be delayed until diarrhoea resolves to ≤ Grade 1 (2-3 stools/day more than pre-treatment frequency). Nal-IRI must not be administered to patients with bowel obstruction, until it is resolved. Following Grade 3 or 4 diarrhoea, the subsequent dose of nal-IRI should be reduced (see section 11.6.1).

The synthetic octapeptide octreotide has been shown to be effective in the control of diarrhoea induced by fluoropyrimidine-based chemotherapy regimens when administered as an escalating dose by continuous infusion or subcutaneous injection. Octreotide can be administered at doses ranging from 100 micrograms twice daily to 500 micrograms three times daily, with a maximum tolerated dose of 2000

micrograms three times daily in a 5-day regimen. Patients should be advised to drink water copiously throughout treatment.

Acute Cholinergic Syndrome
Atropine may be prescribed prophylactically for patients who experienced acute cholinergic symptoms in the previous cycles.

Other treatments
Symptomatic treatment for other toxicities should be per institutional guidelines.

Appendix 5 – Management of docetaxel toxicities (as per local standard practice)

Suggested management could include:

11.4.3.1 Hypersensitivity Reactions
Patients should be observed closely for hypersensitivity reactions, especially during the first and second infusions. In order to reduce the incidence of hypersensitivity all patients should be premedicated with oral steroids (dexamethasone 8mg p.o. b.i.d. x 3 doses). If a significant reaction (Grade 3/Grade 4) occurs despite this, local protocols for the management of hypersensitivity reactions should be followed but would normally include the administration of chlorpheniramine 10-20 mg IV and hydrocortisone 100-500 mg IV.
In extreme cases adrenaline (1:1000 solution 0.5 mL IM, repeated if necessary) may be required.

11.4.3.2 Neutropenia
This is the most frequent adverse reaction of docetaxel. For dose modifications following neutropenia refer to Section 11.6.3.

11.4.3.3 Thrombocytopenia
Patients who develop thrombocytopenia (Plt <100 x 10^9/L) should stop treatment until their platelets count recovers to ≥ 100x10^9/L. Docetaxel can then be restarted as per Section 11.6.3.

11.4.3.4 Neuropathy
Mild sensory neuropathy (Grade 1-2) may develop and persist for 3-4 months following completion of treatment. Patients who develop Grade 3 or 4 peripheral neuropathy should discontinue docetaxel.

11.4.3.5 Hepatic dysfunction
If Bilirubin >1.5 ULN, ALT/AST > 2.5 x ULN (in absence of liver metastasis) or > 5 x ULN (in presence of liver metastasis) treatment with docetaxel should be interrupted until these parameters recover to Grade 1 or baseline levels. Docetaxel can then be restarted as per Section 11.6.3.

11.4.3.6 Cutaneous reaction
If patients develop Grade 2 cutaneous reaction, docetaxel treatment should stop until this reaction resolves to Grade 1 or better. Patients who develop severe or cumulative (Grade 3-Grade 4) cutaneous reaction should stop treatment until resolution of skin changes. In both cases treatment can then be restarted at doses as recommended in Section 11.6.3.

11.4.3.7 Any other Grade 3 or 4 non-haematological toxicity
Withhold docetaxel until parameters recover to baseline levels then restart at reduced dose as per Section 11.6.3.

Patients who have already received two dose reductions and experience further toxicities which would require dose reduction (as defined in Section 11.6.3) should discontinue study medication.

11.4.3.8 Other, common, usually less serious toxicities expected from the use of docetaxel:
Nausea and vomiting: This is usually mild but may occur in the first few days after chemotherapy. Treat with anti-emetics before each cycle of treatment at the discretion of the investigator.

Diarrhoea and Constipation: May occur in the first week of treatment and can be controlled by appropriate medication at the discretion of the investigator.

Alopecia (Hair loss): Usually starts to occur 3 weeks after the first injection of docetaxel and is temporary.

Muscle and joint pain: some patients may start experiencing these after 2-3 days of starting treatment.

Fluid retention: Weight gain and swelling in ankles and legs may occur during treatment but should decrease slowly following completion of treatment.

Effects on fertility and menstruation: Pre-menopausal female patients may notice disruption to their monthly periods and the drug may alter the function of the ovaries therefore affecting fertility. However, all female patients of child bearing potential must take adequate contraception during the course of treatment and for 3 months following completion of treatment. If treatment is interrupted for toxicity the patient must be evaluated until resolution to Grade 0/ Grade 1 or baseline, then the patient can be re-treated provided there is no other reason for stopping treatment (as outlined in Section 11.6.3).

Appendix 6 - Proposed algorithm for diarrhoea management, but local standard practice may be used as an alternative.

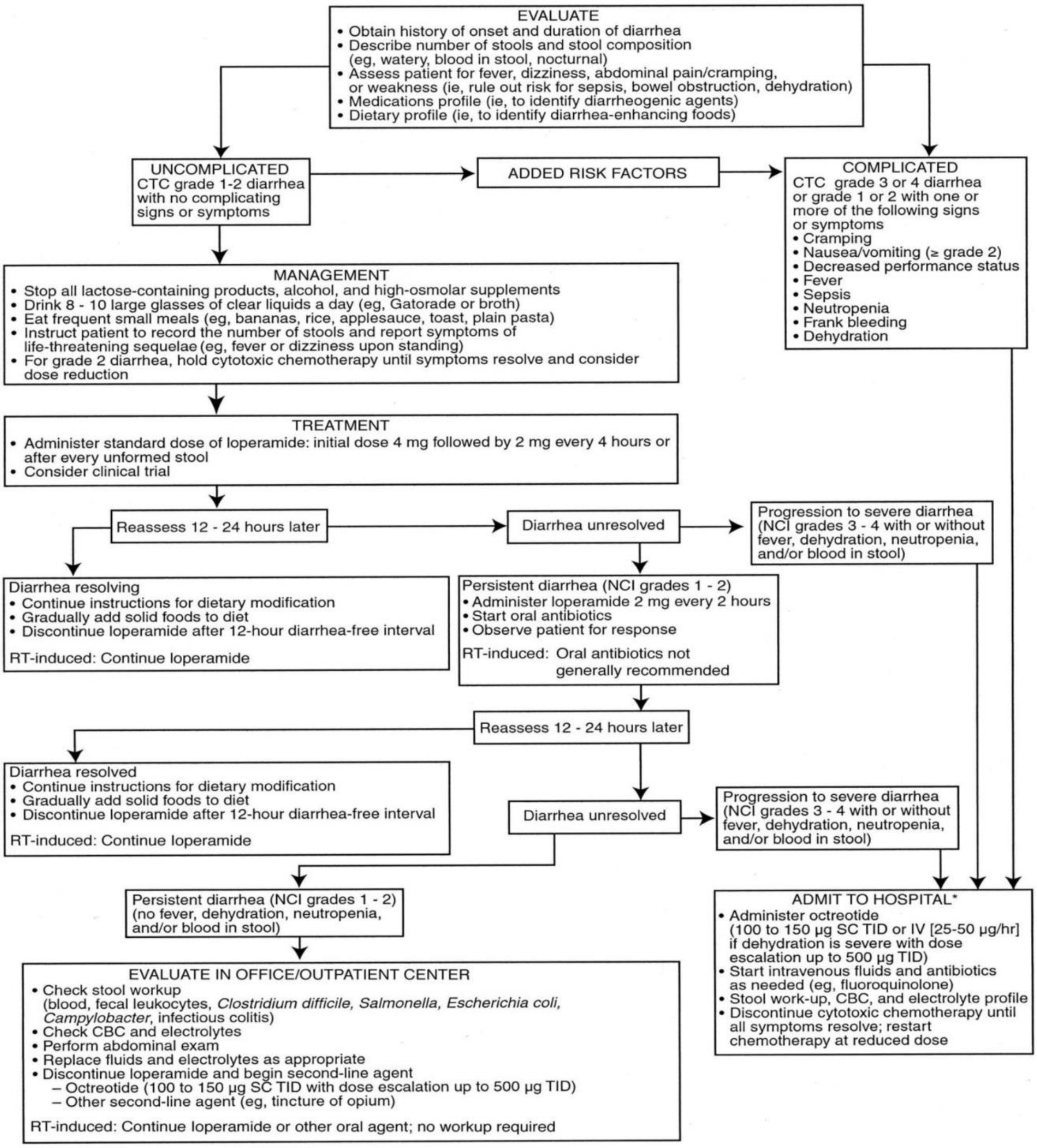

*For radiation-induced cases and select patients with chemotherapy-induced diarrhoea (CID), consider intensive outpatient management, unless the patient has sepsis, fever, or neutropenia.
CTC common toxicity criteria, NCI national cancer institute, RT radiotherapy, SC subcutaneous, tid three times per day, IV intravenous, CBC complete blood count. [49]

Appendix 7 - Management of infusion reactions

The guidelines described in this section can be followed in case of infusion reactions. Infusion reactions will be defined according to the National Cancer Institute Common Terminology Criteria for Adverse Events (CTCAE) (Version 5.0) definitions of an allergic reaction or anaphylaxis as defined below:

Allergic reaction (i.e., a disorder characterised by an adverse local or general response from exposure to an allergen);
Grade 1 Transient flushing or rash, drug fever <38°C; intervention not indicated.
Grade 2 Intervention or infusion interruption indicated; responds promptly to symptomatic treatment (e.g., antihistamines, non-steroidal anti-inflammatory drugs (NSAIDS), narcotics); prophylactic medications indicated for ≤ 24 hrs.
Grade 3 Prolonged (eg not rapidly responsive to symptomatic medication and/or brief interruption of infusion); recurrence of symptoms following initial improvement; hospitalization indicated for clinical sequelae (eg renal impairment, pulmonary infiltrates).
Grade 4 Life-threatening consequences; urgent intervention indicated.

Anaphylaxis (ie a disorder characterised by an acute inflammatory reaction resulting from the release of histamine and histamine-like substances from mast cells, causing hypersensitivity immune response. Clinically, it presents with breathing difficulty, dizziness, hypotension, cyanosis and loss of consciousness and may lead to death);
Grade 1 Not applicable.
Grade 2 Not applicable.
Grade 3 Symptomatic bronchospasm, with or without urticaria; parenteral intervention indicated; allergy-related oedema/angioedema; hypotension.
Grade 4 Life-threatening consequences; urgent intervention indicated.

Institutional policies or the following treatment guidelines shall be used for the management of infusion reactions.

Grade 1

- Slow infusion rate by 50%.
- Monitor patient every 15 minutes for worsening of condition.
- Future infusions may be administered at a reduced rate (e.g. over 120 minutes for nal-IRI), at the discretion of the Investigator.

Grade 2

- Stop infusion.
- Administer diphenhydramine hydrochloride 50 mg IV (or similar), acetaminophen 650 mg (or similar) orally, and oxygen.
- Resume infusion at 50% of the prior rate once infusion reaction has resolved.
- Monitor patient every 15 minutes for worsening of condition.
- For all subsequent infusions, pre-medicate with diphenhydramine hydrochloride 50 mg IV (or similar), dexamethasone 10 mg IV, and acetaminophen 650 mg (or similar) orally.
- Future infusions may be administered at a reduced rate (e.g. over 120 minutes for nal-IRI), at the discretion of the Investigator.

Grade 3

- Stop infusion and disconnect infusion tubing from patient.

- Administer diphenhydramine hydrochloride 50 mg IV (or similar), dexamethasone 10 mg IV, bronchodilators for bronchospasm, and other medications or oxygen as medically necessary.
- No further treatment will be permitted.

Grade 4

- Stop the infusion and disconnect infusion tubing from patient.
- Administer epinephrine (adrenaline), bronchodilators or oxygen as indicated for bronchospasm.
- Administer diphenhydramine hydrochloride 50 mg IV (or similar), dexamethasone 10 mg IV and other medications as medically necessary.
- Consider hospital admission for observation.
- No further treatment will be permitted.

For patients who experience a Grade 1 or Grade 2 infusion reaction, future infusions may be administered at a reduced rate (over 120 minutes), at the discretion of the treating physician.
For patients who experience a second grade 1 or 2 infusion reaction, administer dexamethasone 10 mg IV. All subsequent infusions should be pre-medicated with diphenhydramine hydrochloride 50 mg IV (or similar), dexamethasone 10 mg IV, and acetaminophen 650 mg orally (or similar).

Appendix 8 – Inhibitors and Inducers of CYP3A

Inhibitors and inducers of CYP3A enzymes are defined as follows. Further information can be found in the Onivyde SPC.

**Inhibitors of CYP3A**	**Inducers of CYP3A**
Strong inhibitors	carbamazepine
indinavir	efavirenz
nelfinavir	Nevirapine
ritonavir	barbiturates
clarithromycin	glucocorticoids
itraconazole	modafinil
ketoconazole	oxcarbarzepine
nefazodone	Phenobarbital
saquinavir	Phenytoin
suboxone	Pioglitazone
telithromycin	Rifabutin
cobicistat	Rifampin
boceprevir	St. John's Wort
mibefradil	troglitazone
telaprevir	
troleandomycin	
posaconazole	
Moderate inhibitors	
aprepitant	
amprenavir	
amiodarone	
atazanavir	
Ciprofloxacin	
crizotinib	
darunavir	
dronedarone	
erythromycin	
dilitiazem	
fluconazole	

fosamprenavir	
grapefruit juice	
Seville orange juice	
verapamil	
voriconazole	
imatinib	
Weak inhibitors	
cimetidine	
fluvoxamine	
All other inhibitors	
Chloramphenicol	
Delaviridine	
Diethyl-dithiocarbamate	
Gestodene	
Mifepristone	
Norfloxacin	
Norfluoxetine	
Star fruit	

Appendix 9 – Safety Monitoring Plan

Study Title: **NET-02**				
Risks associated with trial interventions<br>☒ LOW ≡ Comparable to the risk of standard medical care<br>☐ MODERATE ≡ Somewhat higher than the risk of standard medical care<br>☐ HIGH ≡ Markedly higher than the risk of standard medical care				
Justification: *Briefly justify the risk category selected and your conclusions below (where the table is completed in detail the detail need not be repeated, however a summary should be given):*<br><br>This trial involves the use of standard drugs (docetaxel, 5-fluorouracil (5-FU), folinic acid) which have been studied extensively. Irinotecan has been studied extensively in many tumour types and also used in patients with neuroendocrine carcinoma, but the trial uses liposomal encapsulated irinotecan (Nal-IRI), which has a more limited patient exposure and has not been used in patients with neuroendocrine carcinoma. Data from Nal-IRI studies do not show any unexpected toxicity when compared to the active ingredient, irinotecan. Certain known adverse reactions of irinotecan have not been observed with nal-IRI at the time of writing. This could be due to the limited cumulative patient exposure to date of nal-IRI, or the use of appropriate premedication and early recognition and treatment of expected adverse events.				
What are the key risks related to therapeutic interventions you plan to monitor in this trial?		How will these risks be minimised?		
IMP/Intervention	Body system/Hazard	Activity	Frequency	Comments
Liposomal irinotecan	Diarrhoea	Dose adjustments given in section 11.6.1 and management actions recommended in Appendix 4	AE assessment carried out every two weeks.	
	Neutropenia	Dose adjustments given in section 11.6.1 and management actions recommended in Appendix 4	AE assessment carried out every two weeks.	
	Hypersensitivity	Dose adjustments given in section 11.6.1	AE assessment carried out every two weeks.	
	Colitis/Ileus	Dose adjustments given in section 11.6.1	AE assessment carried out every two weeks.	

	Thromboembolism	Dose adjustments given in section 11.6.1	AE assessment carried out every two weeks.	
	Pregnancy	Women of childbearing potential and men of reproductive potential are excluded from trial participation unless using a highly effective form of contraception. A pregnancy test is required prior to randomisation for women of childbearing potential.	AE assessment carried out every two weeks.	
	Intravenous site	Recommendations given in Section 11.4.1	AE assessment carried out every two weeks.	
	Patients with modestly elevated baseline serum total bilirubin levels ((1.0 to 2.0 mg/dL [17.1umol/L to 34.2umol/L]) may have a significantly greater likelihood of experiencing first-cycle grade 3 or 4 neutropenia	Complete blood counts will be monitored at every treatment visit in this patient population. Dose adjustments given in section 11.6.1.	AE assessment carried out every two weeks.	
	Patients with hyperbilirubinaemia are at a greater risk of neutropenia	Complete blood counts will be monitored at every treatment visit in this patient population. Dose adjustments given in section 11.6.1.	AE assessment carried out every two weeks.	
	ECG abnormalities	ECG required prior to randomisation. Management of toxicity recommended in Section 11.6.2.3. Dose adjustments given in section 11.6.2.2.	AE assessment carried out every two weeks.	

Docetaxel	Hypersensitivity reactions	Dose adjustments given in section 11.6.3.	AE assessment carried out every three weeks.	
	Neutropenia	Dose adjustments given in section 11.6.3 and management actions recommended in Appendix 5	AE assessment carried out every three weeks.	
	Thrombocytopenia	Dose adjustments given in section 11.6.3 and management actions recommended in Appendix 5	AE assessment carried out every three weeks.	
	Hepatic dysfunction	Dose adjustments given in section 11.6.3 and management actions recommended in Appendix 5	AE assessment carried out every three weeks.	
	Cutaneous reaction	Dose adjustments given in section 11.6.3	AE assessment carried out every three weeks.	
A data monitoring and ethics committee (DMEC) will be convened who will periodically (at least annually) review unblinded safety information. The DMEC will, in light of these reports, have the authority to recommend trial closure to the Trial Steering Committee (TSC), should they have concerns over the safety or ethics of the trial. The TSC have the authority to recommend closure of the trial to the sponsor at any time.<br>Participant data will be entered into a validated database and monitored for completeness and quality by the Leeds Clinical Trials Unit. Missing data will be chased until it is received, confirmed as not available, or the trial is at analysis stage. A validation check programme will be incorporated into the database to verify the data, and discrepancy reports will be generated for resolution by the local investigator. Priority validations will be incorporated into the validation programme to ensure that any discrepancies related to participant rights, or the safety of participants, are expedited to participating centres for resolution.				
