# Supplementary material for: NET-02: a randomised, non-comparative, phase II trial of nal-IRI/5-FU or docetaxel as second-line therapy in patients with progressive poorly differentiated extra-pulmonary neuroendocrine carcinoma
Source: eClinicalMedicine. 2023 Jun 2;60:102015. doi: 10.1016/j.eclinm.2023.102015 (PMC10242623; doi:10.1016/j.eclinm.2023.102015)
Supplement: Captions for supplementary material [file mmc3.docx]

**Captions for supplementary material**

**Figure S1:** Change from baseline for C30 functional scales by treatment (Nal-IRI (liposomal irinotecan) (left); Docetaxel (right)).

The central vertical line represents the baseline, with the change from baseline for the C30 functional scales for liposomal irinotecan on the left of the central baseline line and for docetaxel on the right.

**Table S1**: Patterns of change in C30 symptom items and GINET21 items from baseline
